# Auditory brainstem responses in the nine-banded armadillo (*Dasypus novemcinctus*)

**DOI:** 10.7717/peerj.16602

**Published:** 2023-12-13

**Authors:** Thomas Brad Moffitt, Samuel Atcherson, Jeffrey Padberg

**Affiliations:** 1 Biology, University of Central Arkansas, Conway, AR, USA; 2 Department of Audiology and Speech Pathology, University of Arkansas for Medical Sciences, Little Rock, AR, United States

**Keywords:** Auditory brainstem response, Evolutionary neurobiology, Xenarthra, Auditory system, Comparative neurobiology

## Abstract

The auditory brainstem response (ABR) to tone burst stimuli of thirteen frequencies ranging from 0.5 to 48 kHz was recorded in the nine-banded armadillo *(Dasypus novemcinctus*), the only extant member of the placental mammal superorder Xenarthra in North America. The armadillo ABR consisted of five main peaks that were visible within the first 10 ms when stimuli were presented at high intensities. The latency of peak I of the armadillo ABR increased as stimulus intensity decreased by an average of 20 μs/dB. Estimated frequency-specific thresholds identified by the ABR were used to construct an estimate of the armadillo audiogram describing the mean thresholds of the eight animals tested. The majority of animals tested (six out of eight) exhibited clear responses to stimuli from 0.5 to 38 kHz, and two animals exhibited responses to stimuli of 48 kHz. Across all cases, the lowest thresholds were observed for frequencies from 8 to 12 kHz. Overall, we observed that the armadillo estimated audiogram bears a similar pattern as those observed using ABR in members of other mammalian clades, including marsupials and later-derived placental mammals.

## Introduction

The nine-banded armadillo (*Dasypus novemcinctus*) is the only extant North American member of superclade Xenarthra, a relatively early-derived clade of placental mammals that includes the extant sloths, anteaters, and armadillos (*e.g*., Delsuc et al., 2002; Gaudin, 2003; Morgan et al., 2013). Xenarthrans are thought to have originated in the South American continent approximately 58 million years ago and spread into North America along the Isthmus of Panama, becoming widespread and diverse during the Neogene period (Scillato-Yané, 1976; Oliveira & Bergqvist, 1998; Berqgvist, Abrantes & Avilla, 2004; Moller-Krull et al., 2007; Webb, 1976; Stehli & Webb, 1985; Vizcaino, Bargo & Farina, 2008; also reviewed in Padberg, 2017). During the Pleistocene, North American xenarthrans went extinct, and diversity in the remaining members of the clade decreased (Delsuc, Vizcaíno & Douzery, 2004; for review, see Vizcaino & Bargo, 2014; Moraes-Barros & Arteaga, 2015). Of the thirty-nine extant xenathran species, all are endemic to South and/or Central America with the exception of nine-banded armadillos, whose range includes a large portion of South, Central, and North America and continues to expand in the United States (*e.g*., Loughry et al., 2013; Superina & Loughry, 2015).

Historically, nine-banded armadillos have been used as a model for Hansen’s disease research (*e.g*., Kirchheimer & Storrs, 1971; Truman et al., 2011; Sharma et al., 2015; for review, see Sharma et al., 2013) or studies of reproductive physiology and development due to their polyembryonic reproduction in which every litter is composed of four identical quadruplets resulting from a single fertilized egg (*e.g*., Loughry et al., 1998, 2015). Less is known about the sensory systems of this species aside from relatively coarse characterizations of the sensory and motor cortical areas (Dom et al., 1971; Royce & Martin, 1975). Recently, renewed interest in characterizing the neural systems of this species has emerged, with a focus on examining the evolution of sensory and motor specializations in xenarthrans, including features such as photoreceptor arrays, orientation selectivity in neurons in primary visual cortex, and transcriptomic organization of motor cortices (Emerling & Springer, 2015; Scholl et al., 2017; Yanny et al., 2021; Wirthlin et al., 2023).

With regard to the auditory system, a relatively large extent of electrophysiologically—defined auditory cortex compared to other sensory regions has been described in the brain of the nine-banded armadillo (Royce & Martin, 1975). The inferior colliculi are large, protruding above the midbrain in dasypoid armadillos (*e.g*., Ferrari, 1998), and a proportionally large subcortical auditory system is observed in armadillos compared to those of many other mammals (*e.g*., Glendenning & Masterton, 1998). Nine-banded armadillos also have large, highly mobile pinnae.

Some aspects of auditory function (frequency range, mean maximum sensitivity, and spectral region of maximum sensitivity) in a few xenarthran species including *Dasypus novemcinctus* have been measured using cochlear microphonic (CM) techniques (Suga, 1967; Peterson & Heaton, 1968). The functional organization of sensory areas including auditory cortex in armadillos has also been examined using evoked potentials from recordings on the cortical surface (Royce & Martin, 1975). The tonotopic organization of the auditory cortex was not examined, although its general caudolateral location on the hemisphere was determined based on evoked responses to a broadband auditory stimulus (the clicking sound of a camera shutter; Royce & Martin, 1975).

As part of a larger effort aimed at determining in detail the functional and anatomical organization of the auditory system in the armadillo, the goal of this study was to determine the frequency range and auditory sensitivity of *Dasypus novemcinctus* using estimated thresholds provided by ABR techniques. The noninvasive ABR has been used for decades to provide basic characterization of functions in the peripheral and central auditory system in a range of species (*e.g*., Jewett & Williston, 1971; Ridgway et al., 1981; Gorga et al., 1988; Reimer, 1995, 1996; Cone-Wesson, Hill & Liu, 1997; Mills & Shepherd, 2001; Wilson & Mills, 2005; Atcherson & Stoody, 2012; Garrett et al., 2019; Tuebenbacher, Doss & Guevar, 2020). Here we describe the morphology, amplitude, and thresholds of the ABR and present an audiogram for anesthetized *D. novemcinctus* based on estimated threshold levels for thirteen frequencies between 0.5 and 48 kHz.

## Materials and Methods

Eight wild-caught armadillos (three female, five male, weight 3.8–6 kg; Table 1) were used for this descriptive study. Ages were unknown, but based upon body size and weight all animals were estimated to be adults. The animals were housed in individual kennels containing corn cob or wood pellet substrate and soft paper bedding. Fresh water and food (Blue brand feline cat food mixed with Blue brand dog food and Vitalize Raw Max powder, supplemented by live earthworms) were provided daily. The quarters were maintained at 22 °C with a 12 h day/night lighting cycle.

**Table 1:** Mass and sex of armadillos in this study.

Case #	Sex	Mass (kg)
15–01	M	5.3
15–02	F	3.4
15–04	F	4.7
15–05	F	3.8
15–06	M	5.2
15–07	M	4.1
15–08	M	5.3
15–08	M	4.4

**Note:**

Three female and five male armadillos were examined in this study, and weights and sex are shown.

**Figure 1:**
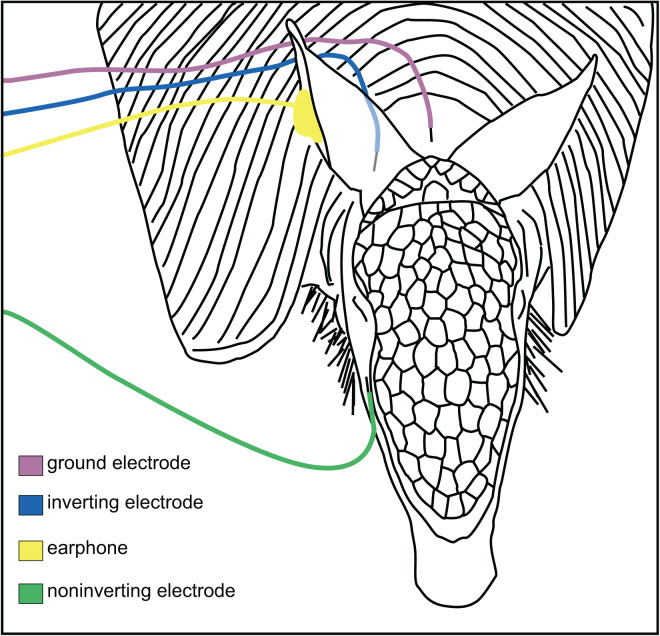
Placement of electrodes on the armadillo head. Placement of subdermal needle electrodes on anesthetized armadillo during ABR experiments using right ear. Noninverting electrode (green) was placed on the snout just lateral to cephalic shield, inverting electrode (blue) was placed immediately caudal to pinna, and ground electrode (rose) was placed along the midline of the dorsal neck. A hollow tube from the transducer was tipped with a form-fitting earplug to fit into the ear canal (yellow). Positions of electrodes and earphone were mirrored for testing the left ear. Adapted from Moffitt (2015).

Animals were anesthetized by isoflurane (1.75–2.5%) delivered *via* nose mask. Once the animals were unresponsive, the skin of the head and dorsal neck was disinfected with 70% ethanol, 4% chlorhexidine gluconate surgical scrub, and sterile stainless steel needle electrodes (Natus Neurology Inc, Warwick, RI, USA) were placed subdermally at the back of the neck (ground), behind the ear (inverting), and cheek (non-inverting; Fig. 1). These positions for the electrodes were chosen to avoid the cephalic shield, the bony plate on the dorsal portion of the armadillo head. The pinna and the ear canal were visually examined to determine they were clear of any obstructions, and a hollow tube with a form-fitting foam tip was placed into the ear canal for stimulus delivery. At the conclusion of each recording session, the anesthesia was withdrawn and once alert, the animals were returned to their home cages. All procedures were approved by the Institutional Animal Care and Use Committee at the University of Central Arkansas (Approval #13-003; UCA PHS (Public Health Service) Animal Welfare Assurance #A4179-01) and followed USDA and NIH guidelines.

To first identify and characterize the physiological ABR response, 50 µs broadband clicks were presented at a rate of 27.7/s and at peak-to-peak equivalent sound pressure levels (peSPL) of 100 dB and lower using a clinical Bio-Logic Navigator Pro system (Natus Neurology Inc. Warwick, RI). Next, tone burst stimuli with 2 ms rise-plateau-fall times (6 ms total duration) were presented at a stimulation rate of 27.7/s from 0.5 to 48 kHz with 13 frequencies of 0.5, 1, 2, 4, 8, 12, 16, 20, 25, 32, 38, 44, and 48 kHz. Stimuli between 0.5 and 2 kHz were generated by a Bio-Logic Navigator Pro system (Natus Neurology Inc., Warwick, RI, USA), which also served as the evoked potential recording system for all responses in this study.

All remaining stimuli (4 kHz and higher) were generated by Spike2 software *via* a linked CED 3505 programmable attenuator, ED-1 speaker driver (Cambridge Electronic Design, Cambridge, UK), and EC-1 transducer (Tucker Davis Technologies, Alachua, FL, USA) with a time-locked trigger sent to the connected Navigator Pro evoked potential system. Peak-to-peak equivalent sound pressure levels (pe SPL) of each tone burst was measured using a Type 2250 sound level meter (Brüel & Kjær, Duluth, GA, USA) coupled to an oscilloscope and checked against the peak-to-peak value of a 94 dB SPL reference tone at 1 kHz using a Type 4231 piston phone (Brüel & Kjær, Duluth, GA, USA; Table 2).

**Table 2:** Minimum and mean ABR thresholds and maximum stimulus levels by frequency.

Frequency	Number of responsive animals/tested	Minimum threshold peSPL (dB)	Mean threshold peSPL (dB)	Standard deviation	Maximum stimulus level peSPL (dB)
0.5 kHZ	6/6	79.9	87.0	5.45	95.0
1 kHz	7/7	69.6	77.6	6.41	94.6
2 kHz	7/7	51.8	66.9	9.62	106.8
4 kHz	8/8	27.7	37.4	7.96	72.7
8 kHz	8/8	1.8	26.1	13.14	69.3
12 kHz	8/8	4.7	23.5	15.40	67.2
16 kHz	8/8	13.1	33.3	14.12	68.1
20 kHz	8/8	26.8	40.2	11.35	71.8
25 kHz	7/7	37	52.4	14.29	77.0
32 kHz	6/8	28.5	43.9	10.31	63.5
38 kHz	4/6	34.7	49.1	12.81	67.2
44 kHz	1/6	38.7	38.7	–	61.2
48 kHz	2/5	30.3	39.1	12.01	52.8

**Note:**

Numbers of animals tested and responsive for the frequencies between 0.5 and 48 kHz, with minimum, mean thresholds, standard deviations, and maximum stimulus level for each frequency.

All recordings were bandpass filtered 30 to 1,500 Hz and collected within a 16 ms time window. Recordings were collected and averaged online until the peakform had stabilized (typically 1,000–1,200 sweeps). ABRs were collected starting with maximum output levels, and intensities were then decreased by 10 dB in successive trials until no visually identifiable response could be measured (Table 2). Thresholds were defined by the lowest stimulus level as follows: (1) at least two repeated responses, (2) a minimum peak I amplitude that is equal to twice the level of the maximum background response, and (3) confirmed by two trained observers. The threshold values were then compiled into Excel (Microsoft, Redmond, WA) to generate descriptive statistics. To assess any potential effects of sex on thresholds across the tested frequencies, we conducted a two-way mixed model ANOVA with frequency as a within-cases variable and sex as a between-groups variable (IBM SPSS Statistics for Windows, version 27 (IBM Corp., Armonk, N.Y., USA)).

## Results

### ABR morphology

**Figure 2:**
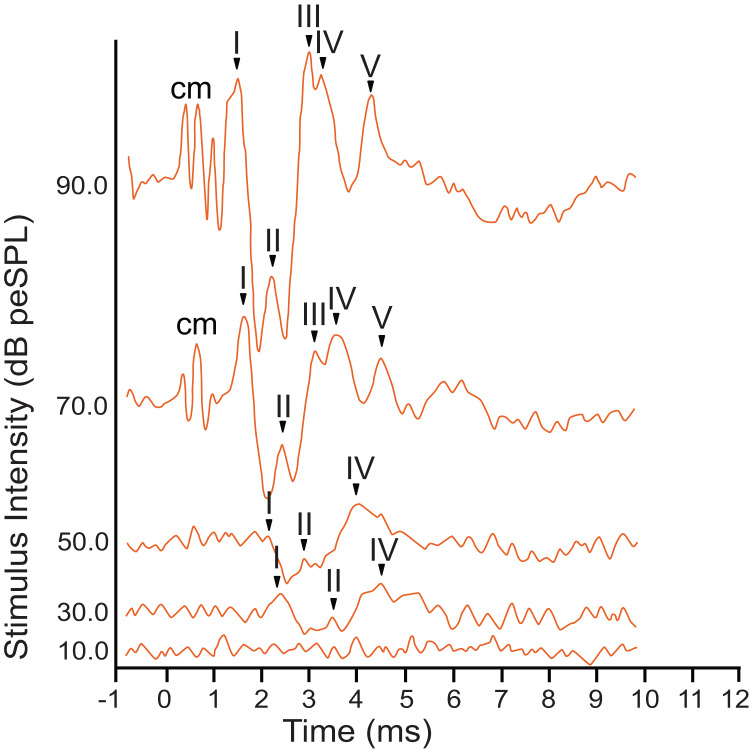
Representative ABR peakform in response to broadband clicks. Representative ABR peakform morphology in response to broadband clicks of alternating polarity stimuli at intensities between 10–90 dB peSPL. At high stimulus intensities (>65 dB peSPL), cochlear microphonic response was evident within 1 ms after stimulation. Peaks I–V were observed in the first 5 ms after stimulation. In all cases, peak I was consistently the peak with highest amplitude and was the most reliably observed peak across all stimulus levels.

The armadillo ABR to broadband clicks at high stimulus intensities (>65 dB) consisted of five peaks between the first 1–5 ms after stimulus onset (Fig. 2). Within the first millisecond after stimulation at intensities greater than 60 dB, and prior to the first peak, evidence of the cochlear microphonic was also observed in the waveform. Peak I was present within the first two milliseconds at high stimulus intensity. The second peak was visible following a deep trough after peak I. The third peak had a relatively high amplitude, and was followed closely by peak IV. The smaller fifth peak was evident within approximately four milliseconds post stimulus and was only observed at relatively high stimulus intensities.

**Figure 3:**
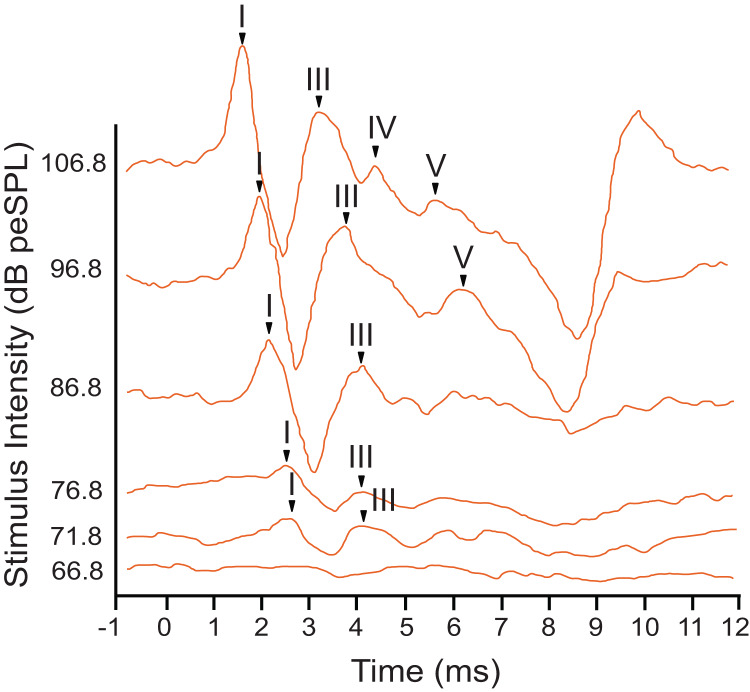
Representative ABR peakform in response to toneburst stimuli. ABR peakform morphology in response to 2 kHz tone burst alternating polarity stimuli at intensities between 65–107 dB peSPL. Stimuli were presented to the left ear at a rate of 27.7 repetitions per second. At highest stimulus intensities, four peaks (I–V) were present, and a negative trend following peak V was evident approximately 7 ms after stimulation. The overall morphology of the peakform changed as stimulus intensity was lowered. As observed with click stimuli, peak 1 consistently had the highest amplitude and was the most reliably observed peak across all tone burst stimulus levels.

The armadillo ABR to tone bursts consisted of up to four peaks within the first 6 ms, followed by a large negative deflection from approximately 6–9 ms (Fig. 3). This negative deflection was most obvious at higher stimulus intensities, and as the intensity of the stimulus was lowered the slope of the negative trend also decreased. Within approximately 20 dB of threshold intensity for each frequency the negative deflection became indistinguishable from background. In contrast to the ABR observed with broadband clicks, peak II was typically absent in responses to tone burst stimuli.

In response to tone bursts, peak I, the neural signal generated by activity in the cochlear nucleus, consistently had the highest amplitude with a maximum of 2.46 µV, and this peak was present at all stimulus levels where a response was observed for each frequency. Response amplitudes decreased as stimuli were attenuated, until later peaks became indistinguishable from the background while peak I and III remained visible. This pattern was consistent for all animals tested, and for all frequencies presented. Due to the reliability of peak I, all amplitude and latency measurements were made using this peak. Peak III was frequently the last peak to remain visible above background, but was not reliably observed across all frequencies or intensities.

### ABR amplitude and latency

**Figure 4:**
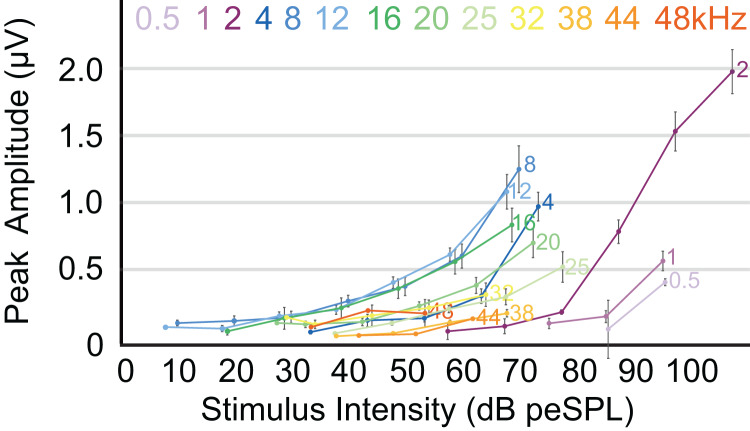
ABR amplitude across selected frequencies. Input-output function of the peak 1 amplitudes for all frequencies tested. Peak amplitudes for all frequencies increased with stimulus intensities. Frequencies are indicated at the right side of the corresponding line. Error bars at each data point indicate mean ± SEM values (*n* = 6 for 0.5 Hz, *n* = 7 for 1 and 2 kHz; *n* = 8 for 4, 8, 12, 16, and 20 kHz; *n* = 7 for 25 kHz, *n* = 6 for 32 kHz; *n* = 4 for 38 kHz, *n* = 1 for 44 kHz, and *n* = 2 for 48 kHz).

**Figure 5:**
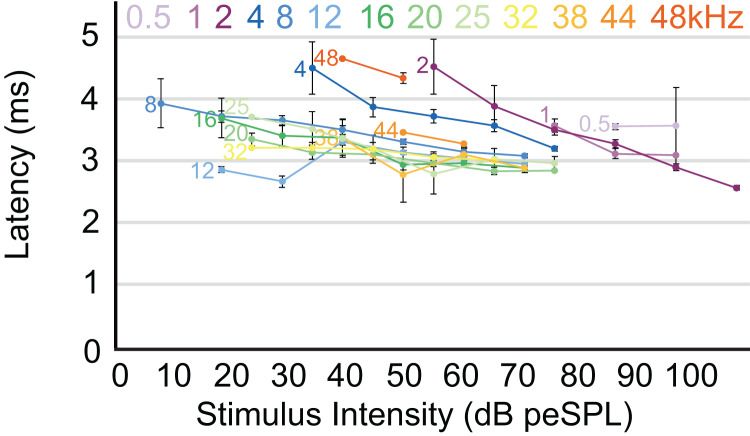
Latency/intensity function for armadillo ABR. Mean latency of wave I as a function of stimulus intensity. Latency/intensity relationships show an average of 0.018 ms increase per dB of attenuation. Stimuli from 0.5–4 kHz had an average latency increase of 0.034 ms per dB of attenuation, while frequencies above 4 kHz had an average latency increase of 0.013 ms per dB of attenuation. Frequencies are indicated at the start of each corresponding line. Error bars indicate SEM values.

The highest amplitude of peak I during unattenuated stimuli varied depending on frequency (Fig. 4). Amplitude of peak I during the lowest frequency stimulus (0.5 kHz) averaged 0.44 µV, and as the frequencies increased the peak amplitude of peak I also increased until 2 kHz, at which the maximum amplitude of 1.97 µV was observed. From 4 to 12 kHz, peak amplitudes were relatively stable, but declined from 12 through 48 kHz. The latency of peak I was similar between the animals in this study and between recording sessions for unattenuated stimuli of the same frequency. Most frequencies tested showed similar peak I latencies, indeed, latencies for nine of the 13 frequencies were within 10 percent of each other (Fig. 5). Unattenuated latencies were longest at the highest frequency tested (48 kHz) and at the lowest frequency tested (0.5 kHz), with mean values of 4.64 and 3.56 ms, respectively. For nearly all frequencies, as stimulus intensities were reduced, latencies increased.

### ABR threshold

**Figure 6:**
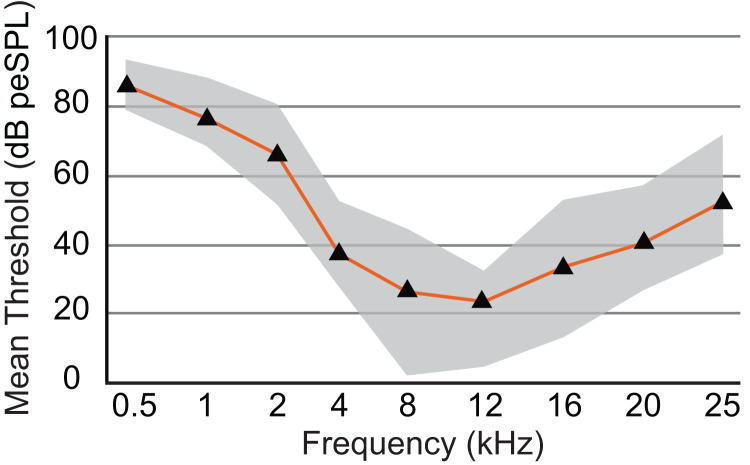
Audiogram of ABR-derived thresholds for *Dasypus novemcinctus*. Audiogram of *D. novemcinctus* estimated from mean ABR thresholds in the current study. Area of greatest sensitivity in armadillos is from 8 to 12 kHz. Maximum and minimum threshold values observed for each frequency are indicated in grey shading. Six to eight animals were tested for each frequency, and all animals were responsive at all frequencies between 0.5 and 25 kHz. At frequencies of 32, 38, 44, and 48 kHz, not all animals were responsive and data are not shown (see Table 2). The lowest mean and minimum thresholds were observed at 8 kHz (26.1 and 1.8 dB peSPL respectively) and 12 kHz (23.5 and 4.7 dB peSPL respectively).

The audiogram was constructed using the mean threshold level at each frequency from all animals tested (Table 2). The 0.5 kHz stimulus was tested on six animals, and five of those exhibited clear responses. ABR responses were reliably obtained from stimuli ranging from 0.5 to 25 kHz in all animals tested, and the lowest thresholds were observed from 8 to 12 kHz (1.8 and 4.7 dB, respectively; Fig. 6). As stimuli from 32 to 48 kHz were less reliable in invoking a response with six of eight animals showing responses to 32 kHz, four of six animals responding to 38 kHz, one of six responding to 44 kHz, and two of five animals responding to 48 kHz, these thresholds are not included in Fig. 6. The audiogram obtained from mean threshold levels is similar in morphology to that obtained from many other mammals which show a region of highest sensitivity towards the middle of the audiogram, and steep decreases in sensitivity in the lower and upper frequencies. The evoked responses in armadillos show a decrease from the region of highest sensitivity to the higher frequency region of the audiogram and appear to plateau between 32 and 48 kHz. Potential effects of sex on auditory thresholds across the frequencies we tested was examined by conducting a two-way mixed model ANOVA with frequency as a within-cases variable and sex as a between-groups variable. There was no effect of sex (*F*(1,6) = 1.385, *p* = 0.284, η_p_^2^ = 0.188) nor any interaction (*F*(4,24) = 1.71, *p* = 0.180, η_p_^2^ = 0.222) between sex and the significant effect of frequency ((*F*(4,24) = 3.89, *p* = 0.014, η_p_^2^ = 0.394); see Supplementary Materials; IBM SPSS Statistics for Windows, version 27 (IBM Corp., Armonk, N.Y., USA)).

## Discussion

**Figure 7:**
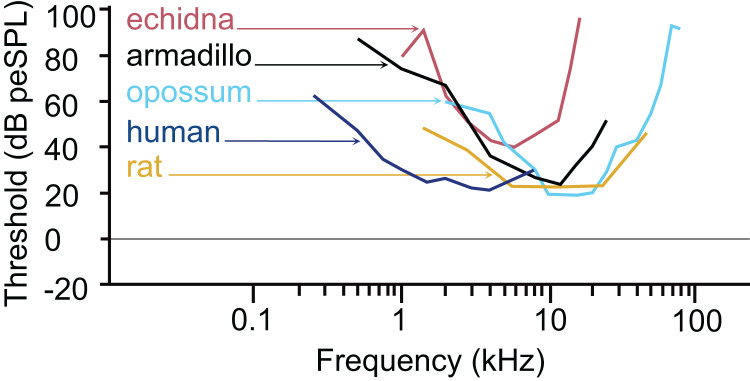
Comparative audiogram. Comparison of ABR-derived audiograms of several mammalian models on the log scale. The estimated threshold range of nine-banded armadillos is relatively wide, with lowest thresholds evident between 8 and 12 kHz. The armadillo audiogram based on the ABR is similar to that of the monotreme short beaked echidna, although the range of highest sensitivity is shifted slightly towards the higher frequencies. Note that different anesthetics were used in some species (human: awake, Gorga et al., 1988; echidna: ketamine/xylazine, Mills & Shepherd, 2001; armadillo: isoflurane, current study; rat: ketamine/xylazine, Powers et al., 2006; opossum: fluanisone/fentanyl citrate, Reimer, 1995). Thin lines with arrowheads point from species to the trace for that species (echidna–red, armadillo–black, opossum–light blue, human–dark blue, rat–goldenrod).

The armadillo ABR to tone bursts consists of up to five peaks at higher stimulus intensities, and often only a single peak as the threshold level is approached. The auditory sensitivity increased sharply from 0.5 to 4 kHz, with the least sensitivity recorded at 0.5 kHz. The results of the present study suggest an auditory frequency range in nine-banded armadillos of 0.5 to 38 kHz. In all subjects, the highest sensitivity was observed between 8 to 12 kHz, with the lowest threshold observed at 8 kHz. The sensitivity to the higher frequencies (from 25 to 48 kHz) was comparatively reduced, with the majority of thresholds in this frequency range near 40 dB. Auditory brainstem responses to frequencies above 38 kHz were not reliably obtained in the current study, with only one case out of the eight tested responding to a 44 kHz stimulus, and two cases responding to 48 kHz. The armadillo audiogram based on ABR is consistent with what is observed in similar studies of representative species from other mammalian clades, including monotremes, marsupials, and eutherians (Fig. 7).

The pattern we observed in peak I latencies for frequencies between 8–12kHz in armadillos was generally flat, between 2.6 and 4 milliseconds, and was consistent with those described in other species. In humans, the lowest ABR thresholds were between 2–5 kHz, with latencies ranging between 7 and 13 ms for 2 kHz for peak V (Gorga et al., 1988). The ABR elicited from pure tone bursts recorded from monotreme echidnas (*Tachyglossus aculeatus*) indicates lowest thresholds between 3 and 8 kHz, with relatively flat latencies between 9–18 ms (Mills & Shepherd, 2001). In marsupial opossums (*Monodelphis domestica*), the lowest thresholds were observed between 8–20 kHz with latencies centered around 2 ms for peak I (Reimer, 1995, 1996). In rats, the lowest ABR thresholds were observed between 6–24kHz, with peak II latencies at approximately 3 ms (Powers et al., 2006). The marsupial tammar wallaby *(Macropus eugenii*) ABR revealed the lowest threshold between 8 and 16 kHz, with latencies between 3.2 and 4.2 ms (Cone-Wesson, Hill & Liu, 1997). In a recent study using ABR in fat-tailed dunnarts to determine estimate thresholds, the thresholds and latencies both decreased with increasing frequency, but frequencies above 47.5 kHz have not been described (Garrett et al., 2019).

The thresholds observed in our preparation in armadillos were similar to thresholds reported from ABR studies in other mammals (Fig. 7). It is important to note that ABR thresholds are generally higher than those derived from behavioral methods and may be more variable between subjects. Examination of studies of human subjects comparing directly comparing threshold measures between ABR and behavioral (psychophysical) methods have shown that ABR thresholds are typically 10–20 dB higher than those observed behaviorally (*e.g*., Gorga et al., 1988; Stapells, 2000). Regardless, the ABR provides a relatively rapid way to estimate auditory function in animals without an extensive behavioral training regimen. Heffner and colleagues have used behavioral methods to generate audiograms for an extensive array of wild and domesticated mammals (*e.g*., Heffner, Heffner & Masterton, 1970; Heffner & Masterton, 1980; Heffner & Heffner, 1984, 1990, 2007; Heffner et al., 1994; Heffner, Koay & Heffner, 2020; for review see Heffner & Heffner, 2014). Future behavioral studies in this species may further describe the characteristics of auditory functions of armadillos.

An earlier study using the cochlear microphonics method reported an upper frequency limit of 60 kHz in *Dasypus novemcinctus* (Peterson & Heaton, 1968), however, in the current study we were unable to test frequencies higher than 48 kHz due to limitations of our equipment. Our results indicate a region of maximum sensitivity between 8 and 12 kHz, which is a narrower band than that reported by Peterson & Heaton (1968), who reported maximum sensitivity for this species between 3 and 25 kHz.

In many species, hearing sensitivity changes with age, with auditory thresholds tending to increase while the frequency range that the animal can detect decreases (*e.g*., Engle, Tinling & Recanzone, 2013; for review, see: Recanzone, 2018; and Keithley, 2019). While we are unable to confirm whether this is true for armadillos because the animals used in this study were wild caught and thus their actual ages were unknown, we did examine any potential effects of sex on thresholds across the frequencies we tested. A two-way mixed model ANOVA with frequency as a within-cases variable and sex as a between-groups variable was performed. There was no significant difference in thresholds between male and female armadillos. We tested only three female animals and five males, and if there are subtle differences, it may not be evident in our study. A follow up study using captive-born animals with a known age and/or using larger numbers of animals of both sexes may be helpful to determine the effects of age and sex on threshold levels and ability to detect frequencies at the more extreme ends of the audiogram in nine-banded armadillos.

It is established that high frequency hearing is important to localize sound sources (*e.g*., Masterton, Heffner & Ravizza, 1969; Heffner & Heffner, 2014; Heffner & Heffner, 2018) and that animals with smaller heads typically rely more on higher frequencies to localize sound (for review see Heffner & Heffner, 2008. Factors such as functional interaural distance (*e.g*., time between arrival of sound at each ear) and interaural intensity differences play a role (Masterton, Heffner & Ravizza, 1969; for review, see Heffner & Heffner, 2018). The armadillo has an interaural separation of approximately 2.5 cm, which should provide a functional interaural time difference of about 75 µs. In addition, the relatively large pinnae compared to their head size may help amplify sounds of interest as the pinnae are moved. It is unclear whether armadillos use both time and intensity cues, as these animals have not been tested in a behavioral paradigm. Interestingly, some fossorial species have lost the ability to detect high frequencies, as azimuthal orientation may be less relevant in an underground environment like a burrow (mole rat: Bruns et al., 1988; Muller & Burda, 1989; pocket gopher: Heffner & Heffner, 1990). The stem xenarthran may also have been fossorial (*e.g*., Simpson, 1931; also see: Emerling & Springer, 2015 for discussion); however, the data in the current study support that nine-banded armadillos retain the ability to detect high frequencies.

An important role of higher frequency hearing is to help direct the visual system to those sound sources (*e.g*., Heffner & Heffner, 1984, 2018). Although to date, the visual fields in this species are not described, armadillos have laterally placed eyes, are not known to have a visual streak in the retina (St. Jules, 1984), and do not have cone photoreceptors (Emerling & Springer, 2015). Thus, localization of the sound source to direct the eyes may be relatively limited in this species.

While ABR and behavioral studies can help determine the functional characteristics of mammalian auditory systems, recent advanced in molecular genetics are rapidly increasing our ability to examine gene expression in the brain. Molecular techniques including single nucleus transcriptomics have recently been used to compare neural structures across species (Tosches & Laurent, 2019; Hodge et al., 2019; Kozlenkov et al., 2020; Bakken et al., 2021). Future transcriptomic examination of the armadillo auditory cortex and thalamus may reveal whether specific genes thought to be critical for high frequency hearing (*e.g*., *Shh* and *SK2*) are present in xenarthrans as they are in echolocators and other mammalian species (*e.g*., Teeling, Jones & Rossiter, 2016; Wang et al., 2021; Concas et al., 2021).

## Conclusions

Here we report for the first time the audiogram of the nine-banded armadillo using ABR methods. We observed that the nine-banded armadillos (*Dasypus novemcinctus*) in this study have a hearing range from 0.5 to 48 kHz, with best sensitivity in the range from 8 to 12 kHz. The audiogram for this species is the typical pattern observed in all mammalian species that have been examined using ABR. The current findings will be used for studies examining the tonotopic organization within the auditory cortex in armadillos. It would be of further interest to gather behavioral thresholds for auditory function in this species, as well as examine how the hearing-related genes in this species may compare to members of other mammalian clades.

## Supplemental Information

10.7717/peerj.16602/supp-1 Supplemental Information 1 ARRIVE checklist. Section and line numbers are indicated for each section. In some cases, n/a indicates not applicable because this was not a treatment study.

10.7717/peerj.16602/supp-2 Supplemental Information 2 ABR waveform data for the armadillos in this study. Each raw data file shows ABR amplitude (blue line) across various stimulus intensities (indicated on y-axis) over time in milliseconds (indicated on x-axis) for a particular experiment.

10.7717/peerj.16602/supp-3 Supplemental Information 3 Raw threshold data and latency data for all armadillo cases.

10.7717/peerj.16602/supp-4 Supplemental Information 4 ANOVA for sex differences for auditory thresholds in armadillos. A 2-way mixed model ANOVA with frequency as a within-cases variable and sex as a between-groups variable was conducted (IBM SPSS Statistics for Windows, version 27 (IBM Corp., Armonk, N.Y., USA)). No effect of sex (*F*(1,6) = 1.385, *p* = 0.284, η_p_^2^ = 0.188) nor any interaction (*F*(4,24) = 1.71, *p* = 0.180, η_p_^2^ = 0.222) between sex and the significant effect of frequency ((*F*(4,24) = 3.89, *p* = 0.014, η_p_^2^ = 0.394) was observed.

## Additional Information and Declarations

### Data Availability

The following information was supplied regarding data availability: The raw ABR data files from the clinical Biologic Navigator Pro ABR system are available in the Supplemental File.
