# Supplementary material for: Auditory brainstem responses in the nine-banded armadillo (Dasypus novemcinctus)
Source: PeerJ. 2023 Dec 13;11:e16602. doi: 10.7717/peerj.16602 (PMC10725177; doi:10.7717/peerj.16602)
Supplement: Supplemental Information 2 — Each raw data file shows ABR amplitude (blue line) across various stimulus intensities (indicated on y-axis) over time in milliseconds (indicated on x-axis) for a particular experiment. [file peerj-11-16602-s002.zip › Armadillo 2021/#1 Animal F14-06 Case 15-01/All other frequencies by record number.pdf]

EVOKED POTENTIAL REPORT

UAMS CHP Speech and Hearing Clinic
Department of Audiology and Speech Pathology
4021 W. 8th Street
Little Rock, AR 72204
(501) 320-7300

Patient: **armadillo1501, armadillo1501**

ID#: **armadillo1501**

Gender: **Male**

Birth date: **02/09/15**

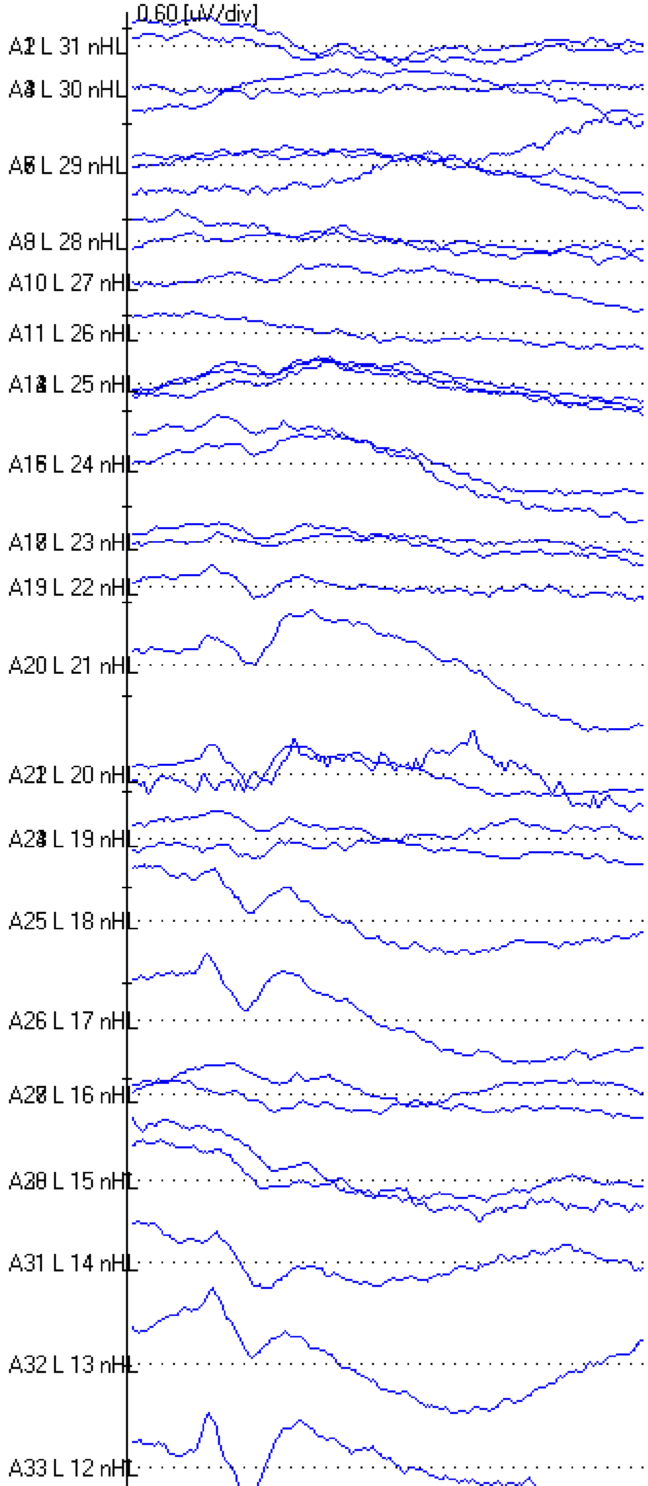

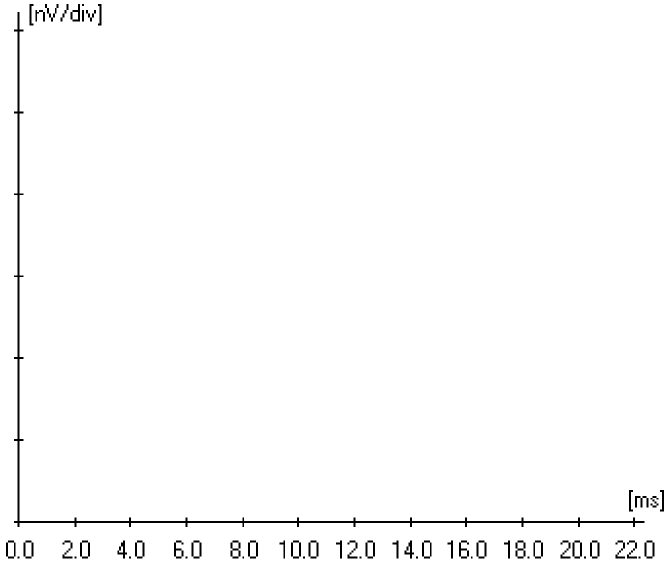

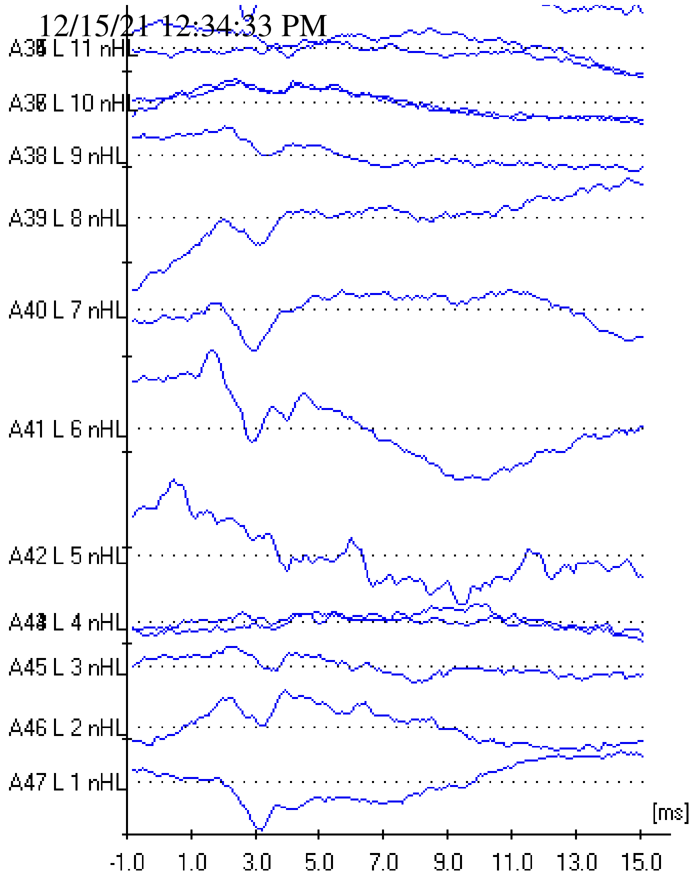

Latencies (ms)

Label Index	*I*	*II*	*III*	*IV*	*V*

Interlatencies (ms)

Label Index	*I-III*	*III-V*	*I-V*

Interaural Latency Differences

Label Index	*L1*	*L2*	*L3*	*L4*	*L5*	*L6*	*L7*	*L8*	*L9*	*L10*

Stimulus Parameters

Label Index	*Intensity*	*Ear*	*Transducer*	*Insert Delay*	*Type*	*Frequency*	*Polarity*	*Ramp*	*Rise/Fall*	*Plateau*	*Rate*
**A1**	**31dB nHL**	**Left**	**Insert Earphones**	**0.80**	**Tone Burst**	**2000**	**Alternating**	**Blackman**	**2.00**	**2.00**	**27.70**
**A2**	**31dB nHL**	**Left**	**Insert Earphones**	**0.80**	**Tone Burst**	**2000**	**Alternating**	**Blackman**	**2.00**	**2.00**	**27.70**
**A3**	**30dB nHL**	**Left**	**Insert Earphones**	**0.80**	**Tone Burst**	**2000**	**Alternating**	**Blackman**	**2.00**	**2.00**	**27.70**
**A4**	**30dB nHL**	**Left**	**Insert Earphones**	**0.80**	**Tone Burst**	**2000**	**Alternating**	**Blackman**	**2.00**	**2.00**	**27.70**
**A5**	**29dB nHL**	**Left**	**Insert Earphones**	**0.80**	**Tone Burst**	**2000**	**Alternating**	**Blackman**	**2.00**	**2.00**	**27.70**
**A6**	**29dB nHL**	**Left**	**Insert Earphones**	**0.80**	**Tone Burst**	**2000**	**Alternating**	**Blackman**	**2.00**	**2.00**	**27.70**
**A7**	**29dB nHL**	**Left**	**Insert Earphones**	**0.80**	**Tone Burst**	**2000**	**Alternating**	**Blackman**	**2.00**	**2.00**	**27.70**
**A8**	**28dB nHL**	**Left**	**Insert Earphones**	**0.80**	**Tone Burst**	**2000**	**Alternating**	**Blackman**	**2.00**	**2.00**	**27.70**
**A9**	**28dB nHL**	**Left**	**Insert Earphones**	**0.80**	**Tone Burst**	**2000**	**Alternating**	**Blackman**	**2.00**	**2.00**	**27.70**
**A10**	**27dB nHL**	**Left**	**Insert Earphones**	**0.80**	**Tone Burst**	**2000**	**Alternating**	**Blackman**	**2.00**	**2.00**	**27.70**
**A11**	**26dB nHL**	**Left**	**Insert Earphones**	**0.80**	**Tone Burst**	**2000**	**Alternating**	**Blackman**	**2.00**	**2.00**	**27.70**
**A12**	**25dB nHL**	**Left**	**Insert Earphones**	**0.80**	**Tone Burst**	**2000**	**Alternating**	**Blackman**	**2.00**	**2.00**	**27.70**

A13	25dB nHL	Left	Insert Earphones	0.80	Tone Burst	2000	Alternating	Blackman	2.00	2.00	27.70
A14	25dB nHL	Left	Insert Earphones	0.80	Tone Burst	2000	Alternating	Blackman	2.00	2.00	27.70
A15	24dB nHL	Left	Insert Earphones	0.80	Tone Burst	2000	Alternating	Blackman	2.00	2.00	27.70
A16	24dB nHL	Left	Insert Earphones	0.80	Tone Burst	2000	Alternating	Blackman	2.00	2.00	27.70
A17	23dB nHL	Left	Insert Earphones	0.80	Tone Burst	2000	Alternating	Blackman	2.00	2.00	27.70
A18	23dB nHL	Left	Insert Earphones	0.80	Tone Burst	2000	Alternating	Blackman	2.00	2.00	27.70
A19	22dB nHL	Left	Insert Earphones	0.80	Tone Burst	2000	Alternating	Blackman	2.00	2.00	27.70
A20	21dB nHL	Left	Insert Earphones	0.80	Tone Burst	2000	Alternating	Blackman	2.00	2.00	27.70
A21	20dB nHL	Left	Insert Earphones	0.80	Tone Burst	2000	Alternating	Blackman	2.00	2.00	27.70
A22	20dB nHL	Left	Insert Earphones	0.80	Tone Burst	2000	Alternating	Blackman	2.00	2.00	27.70
A23	19dB nHL	Left	Insert Earphones	0.80	Tone Burst	2000	Alternating	Blackman	2.00	2.00	27.70
A24	19dB nHL	Left	Insert Earphones	0.80	Tone Burst	2000	Alternating	Blackman	2.00	2.00	27.70
A25	18dB nHL	Left	Insert Earphones	0.80	Tone Burst	2000	Alternating	Blackman	2.00	2.00	27.70
A26	17dB nHL	Left	Insert Earphones	0.80	Tone Burst	2000	Alternating	Blackman	2.00	2.00	27.70
A27	16dB nHL	Left	Insert Earphones	0.80	Tone Burst	2000	Alternating	Blackman	2.00	2.00	27.70
A28	16dB nHL	Left	Insert Earphones	0.80	Tone Burst	2000	Alternating	Blackman	2.00	2.00	27.70
A29	15dB nHL	Left	Insert Earphones	0.80	Tone Burst	2000	Alternating	Blackman	2.00	2.00	27.70
A30	15dB nHL	Left	Insert Earphones	0.80	Tone Burst	2000	Alternating	Blackman	2.00	2.00	27.70
A31	14dB nHL	Left	Insert Earphones	0.80	Tone Burst	2000	Alternating	Blackman	2.00	2.00	27.70
A32	13dB nHL	Left	Insert Earphones	0.80	Tone Burst	2000	Alternating	Blackman	2.00	2.00	27.70
A33	12dB nHL	Left	Insert Earphones	0.80	Tone Burst	2000	Alternating	Blackman	2.00	2.00	27.70
A34	11dB nHL	Left	Insert Earphones	0.80	Tone Burst	2000	Alternating	Blackman	2.00	2.00	27.70
A35	11dB nHL	Left	Insert Earphones	0.80	Tone Burst	2000	Alternating	Blackman	2.00	2.00	27.70
A36	10dB nHL	Left	Insert Earphones	0.80	Tone Burst	2000	Alternating	Blackman	2.00	2.00	27.70
A37	10dB nHL	Left	Insert Earphones	0.80	Tone Burst	2000	Alternating	Blackman	2.00	2.00	27.70
A38	9dB nHL	Left	Insert Earphones	0.80	Tone Burst	2000	Alternating	Blackman	2.00	2.00	27.70
A39	8dB nHL	Left	Insert Earphones	0.80	Tone Burst	2000	Alternating	Blackman	2.00	2.00	27.70
A40	7dB nHL	Left	Insert Earphones	0.80	Tone Burst	2000	Alternating	Blackman	2.00	2.00	27.70
A41	6dB nHL	Left	Insert Earphones	0.80	Tone Burst	2000	Alternating	Blackman	2.00	2.00	27.70
A42	5dB nHL	Left	Insert Earphones	0.80	Tone Burst	2000	Alternating	Blackman	2.00	2.00	27.70
A43	4dB nHL	Left	Insert Earphones	0.80	Tone Burst	2000	Alternating	Blackman	2.00	2.00	27.70
A44	4dB nHL	Left	Insert Earphones	0.80	Tone Burst	2000	Alternating	Blackman	2.00	2.00	27.70
A45	3dB nHL	Left	Insert Earphones	0.80	Tone Burst	2000	Alternating	Blackman	2.00	2.00	27.70
A46	2dB nHL	Left	Insert Earphones	0.80	Tone Burst	2000	Alternating	Blackman	2.00	2.00	27.70
A47	1dB nHL	Left	Insert Earphones	0.80	Tone Burst	2000	Alternating	Blackman	2.00	2.00	27.70

Recording Parameters

Label Index	*Epoch*	*Points*	*Pre/Post*	*Averages*	*Artifacts*
A1	16.00	256	0.00	1576	33
A2	16.00	256	0.00	1274	26
A3	16.00	256	0.00	1684	31
A4	16.00	256	0.00	1939	42
A5	16.00	256	0.00	695	23

A6	16.00	256	0.00	1994	34
A7	16.00	256	0.00	1923	43
A8	16.00	256	0.00	2036	44
A9	16.00	256	0.00	1761	34
A10	16.00	256	0.00	3102	53
A11	16.00	256	0.00	2113	30
A12	16.00	256	0.00	2080	29
A13	16.00	256	0.00	2007	30
A14	16.00	256	0.00	2313	31
A15	16.00	256	0.00	1729	25
A16	16.00	256	0.00	927	14
A17	16.00	256	0.00	1505	27
A18	16.00	256	0.00	2675	33
A19	16.00	256	0.00	1216	13
A20	16.00	256	0.00	1424	22
A21	16.00	256	0.00	2061	50
A22	16.00	256	0.00	3208	45
A23	16.00	256	0.00	1452	36
A24	16.00	256	0.00	2688	67
A25	16.00	256	0.00	889	22
A26	16.00	256	0.00	1826	37
A27	16.00	256	0.00	2159	38
A28	16.00	256	0.00	1828	38
A29	16.00	256	0.00	1487	33
A30	16.00	256	0.00	641	19
A31	16.00	256	0.00	989	25
A32	16.00	256	0.00	891	25
A33	16.00	256	0.00	681	24
A34	16.00	256	0.00	2467	58
A35	16.00	256	0.00	2007	55
A36	16.00	256	0.00	2145	48
A37	16.00	256	0.00	2108	48
A38	16.00	256	0.00	1232	35
A39	16.00	256	0.00	1040	27
A40	16.00	256	0.00	905	23
A41	16.00	256	0.00	811	24
A42	16.00	256	0.00	2116	54
A43	16.00	256	0.00	1465	34
A44	16.00	256	0.00	2142	39
A45	16.00	256	0.00	1304	32
A46	16.00	256	0.00	704	12
A47	16.00	256	0.00	2327	724

Amplifier Parameters

Label Index	*Channel*	*Gain*	*Low Filter*	*High Filter*	*Notch Filter*	*Artifact Rejection*	*Input 1*	*Input 2*
**A1**	**1**	**100000**	**30**	**1500**	**No**	**50.00**	**FZ**	**A1A2**
**A2**	**1**	**100000**	**30**	**1500**	**No**	**50.00**	**FZ**	**A1A2**
**A3**	**1**	**100000**	**30**	**1500**	**No**	**50.00**	**FZ**	**A1A2**
**A4**	**1**	**100000**	**30**	**1500**	**No**	**50.00**	**FZ**	**A1A2**
**A5**	**1**	**100000**	**30**	**1500**	**No**	**50.00**	**FZ**	**A1A2**
**A6**	**1**	**100000**	**30**	**1500**	**No**	**50.00**	**FZ**	**A1A2**
**A7**	**1**	**100000**	**30**	**1500**	**No**	**50.00**	**FZ**	**A1A2**
**A8**	**1**	**100000**	**30**	**1500**	**No**	**50.00**	**FZ**	**A1A2**
**A9**	**1**	**100000**	**30**	**1500**	**No**	**50.00**	**FZ**	**A1A2**
**A10**	**1**	**100000**	**30**	**1500**	**No**	**50.00**	**FZ**	**A1A2**
**A11**	**1**	**100000**	**30**	**1500**	**No**	**50.00**	**FZ**	**A1A2**
**A12**	**1**	**100000**	**30**	**1500**	**No**	**50.00**	**FZ**	**A1A2**
**A13**	**1**	**100000**	**30**	**1500**	**No**	**50.00**	**FZ**	**A1A2**
**A14**	**1**	**100000**	**30**	**1500**	**No**	**50.00**	**FZ**	**A1A2**
**A15**	**1**	**100000**	**30**	**1500**	**No**	**50.00**	**FZ**	**A1A2**
**A16**	**1**	**100000**	**30**	**1500**	**No**	**50.00**	**FZ**	**A1A2**
**A17**	**1**	**100000**	**30**	**1500**	**No**	**50.00**	**FZ**	**A1A2**
**A18**	**1**	**100000**	**30**	**1500**	**No**	**50.00**	**FZ**	**A1A2**
**A19**	**1**	**100000**	**30**	**1500**	**No**	**50.00**	**FZ**	**A1A2**
**A20**	**1**	**100000**	**30**	**1500**	**No**	**50.00**	**FZ**	**A1A2**
**A21**	**1**	**100000**	**30**	**1500**	**No**	**50.00**	**FZ**	**A1A2**
**A22**	**1**	**100000**	**30**	**1500**	**No**	**50.00**	**FZ**	**A1A2**
**A23**	**1**	**100000**	**30**	**1500**	**No**	**50.00**	**FZ**	**A1A2**
**A24**	**1**	**100000**	**30**	**1500**	**No**	**50.00**	**FZ**	**A1A2**
**A25**	**1**	**100000**	**30**	**1500**	**No**	**50.00**	**FZ**	**A1A2**
**A26**	**1**	**100000**	**30**	**1500**	**No**	**50.00**	**FZ**	**A1A2**
**A27**	**1**	**100000**	**30**	**1500**	**No**	**50.00**	**FZ**	**A1A2**
**A28**	**1**	**100000**	**30**	**1500**	**No**	**50.00**	**FZ**	**A1A2**
**A29**	**1**	**100000**	**30**	**1500**	**No**	**50.00**	**FZ**	**A1A2**
**A30**	**1**	**100000**	**30**	**1500**	**No**	**50.00**	**FZ**	**A1A2**
**A31**	**1**	**100000**	**30**	**1500**	**No**	**50.00**	**FZ**	**A1A2**
**A32**	**1**	**100000**	**30**	**1500**	**No**	**50.00**	**FZ**	**A1A2**
**A33**	**1**	**100000**	**30**	**1500**	**No**	**50.00**	**FZ**	**A1A2**
**A34**	**1**	**100000**	**30**	**1500**	**No**	**50.00**	**FZ**	**A1A2**
**A35**	**1**	**100000**	**30**	**1500**	**No**	**50.00**	**FZ**	**A1A2**
**A36**	**1**	**100000**	**30**	**1500**	**No**	**50.00**	**FZ**	**A1A2**
**A37**	**1**	**100000**	**30**	**1500**	**No**	**50.00**	**FZ**	**A1A2**
**A38**	**1**	**100000**	**30**	**1500**	**No**	**50.00**	**FZ**	**A1A2**
**A39**	**1**	**100000**	**30**	**1500**	**No**	**50.00**	**FZ**	**A1A2**
**A40**	**1**	**100000**	**30**	**1500**	**No**	**50.00**	**FZ**	**A1A2**

A41	1	100000	30	1500	No	50.00	FZ	A1A2
A42	1	100000	30	1500	No	50.00	FZ	A1A2
A43	1	100000	30	1500	No	50.00	FZ	A1A2
A44	1	100000	30	1500	No	50.00	FZ	A1A2
A45	1	100000	30	1500	No	50.00	FZ	A1A2
A46	1	100000	30	1500	No	50.00	FZ	A1A2
A47	1	100000	30	1500	No	50.00	FZ	A1A2
