# Supplementary material for: Auditory brainstem responses in the nine-banded armadillo (Dasypus novemcinctus)
Source: PeerJ. 2023 Dec 13;11:e16602. doi: 10.7717/peerj.16602 (PMC10725177; doi:10.7717/peerj.16602)
Supplement: Supplemental Information 2 — Each raw data file shows ABR amplitude (blue line) across various stimulus intensities (indicated on y-axis) over time in milliseconds (indicated on x-axis) for a particular experiment. [file peerj-11-16602-s002.zip › Armadillo 2021/#2 Animal 15-02 Case 15-09/All other frequencies by record number.pdf]

EVOKED POTENTIAL REPORT

UAMS CHP Speech and Hearing Clinic
Department of Audiology and Speech Pathology
4021 W. 8th Street
Little Rock, AR 72204
(501) 320-7300

Patient:	**Armadillo 1509, Armadillo 1509**
ID#:	**Armadillo 1509**
Gender:	
Birth date:	**03/02/15**

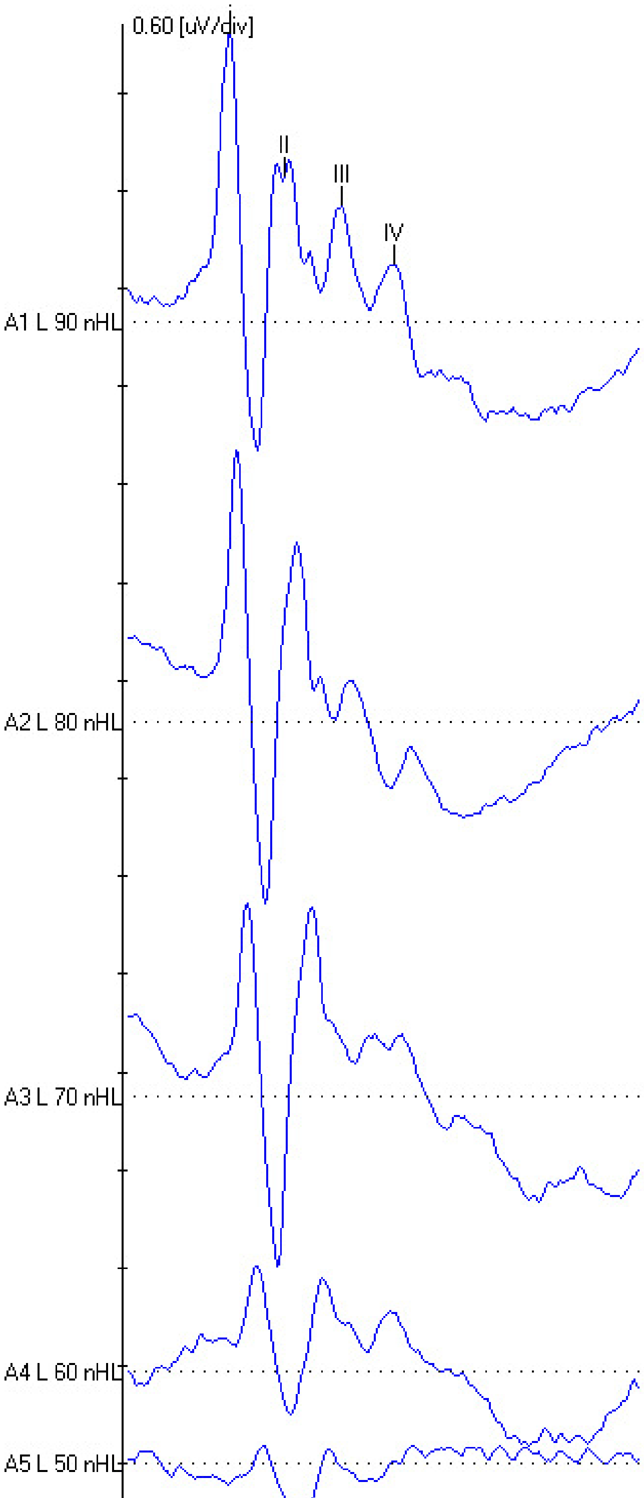

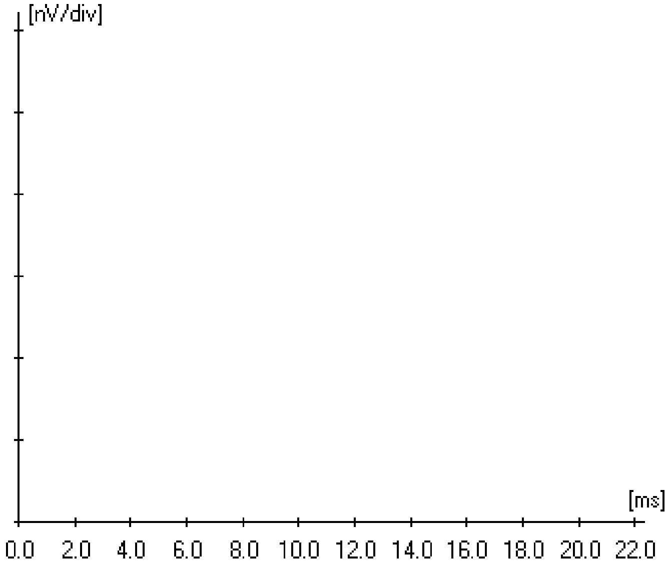

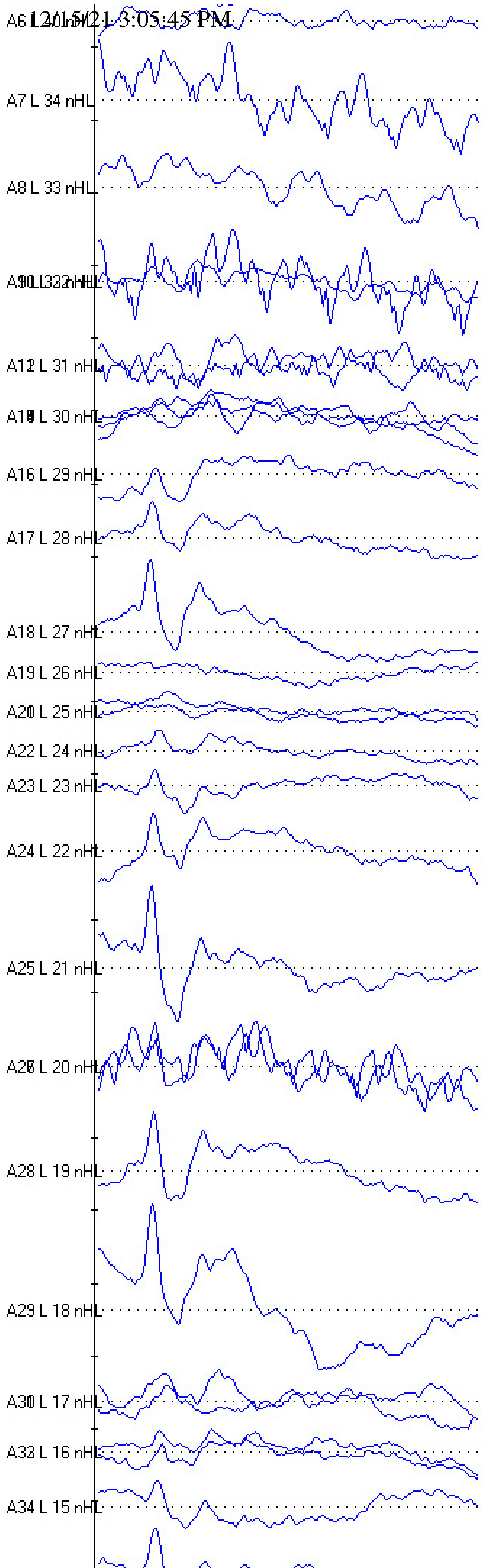
A7 L 34 nHL
A8 L 33 nHL
A16 L 29 nHL
A17 L 28 nHL
A18 L 27 nHL
A19 L 26 nHL
A22 L 24 nHL
A23 L 23 nHL
A24 L 22 nHL
A25 L 21 nHL
A28 L 19 nHL
A29 L 18 nHL
A34 L 15 nHL

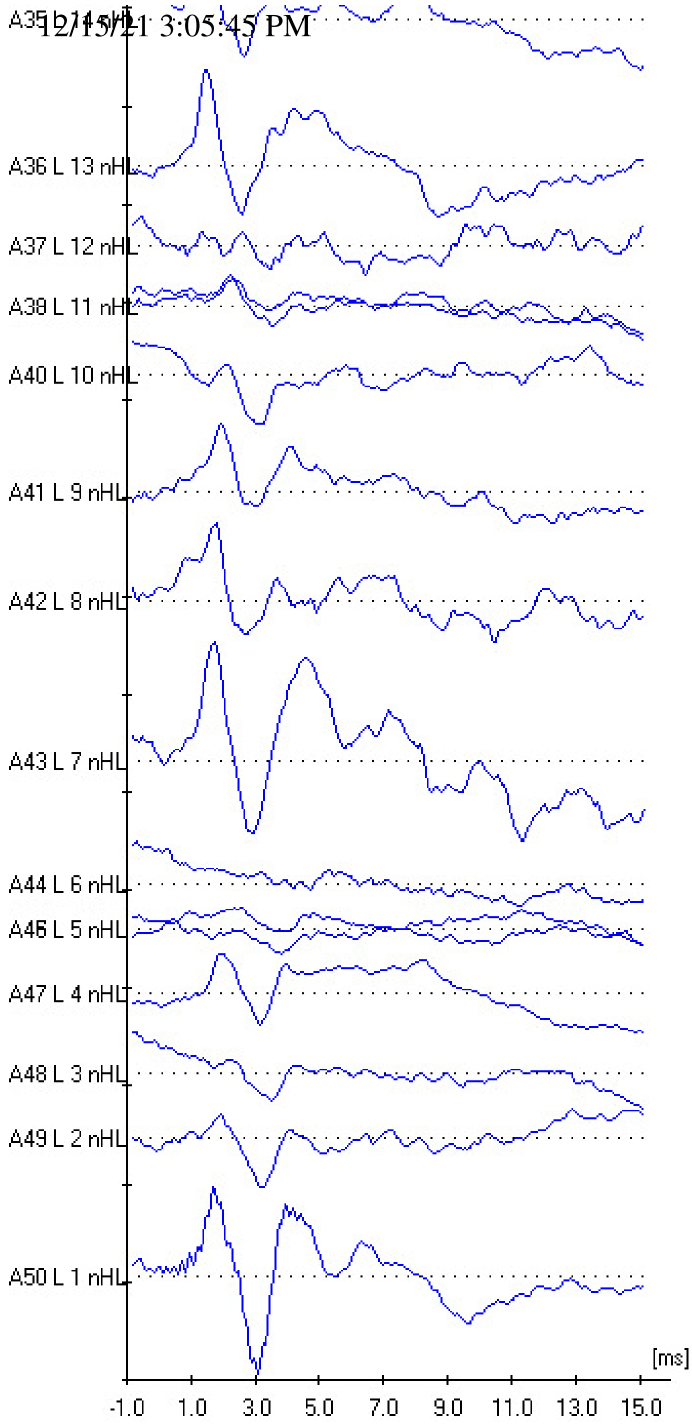

Latencies (ms)

Label Index	*I*	*II*	*III*	*IV*	*V*
**A1**	**2.33**	**4.07**	**5.82**	**7.45**	

Interlatencies (ms)

Label Index	*I-III*	*III-V*	*I-V*
**A1**	**3.50**		

Interaural Latency Differences

Label Index	*L1*	*L2*	*L3*	*L4*	*L5*	*L6*	*L7*	*L8*	*L9*	*L10*

Stimulus Parameters

Label Index	*Intensity*	*Ear*	*Transducer*	*Insert Delay*	*Type*	*Frequency*	*Polarity*	*Ramp*	*Rise/Fall*	*Plateau*	*Rate*
**A1**	**90dB nHL**	**Left**	**Insert Earphones**	**0.80**	**Tone Burst**	**4000**	**Alternating**	**Blackman**	**2.00**	**2.00**	**27.70**
**A2**	**80dB nHL**	**Left**	**Insert Earphones**	**0.80**	**Tone Burst**	**4000**	**Alternating**	**Blackman**	**2.00**	**2.00**	**27.70**
**A3**	**70dB nHL**	**Left**	**Insert Earphones**	**0.80**	**Tone Burst**	**4000**	**Alternating**	**Blackman**	**2.00**	**2.00**	**27.70**
**A4**	**60dB nHL**	**Left**	**Insert Earphones**	**0.80**	**Tone Burst**	**4000**	**Alternating**	**Blackman**	**2.00**	**2.00**	**27.70**
**A5**	**50dB nHL**	**Left**	**Insert Earphones**	**0.80**	**Tone Burst**	**4000**	**Alternating**	**Blackman**	**2.00**	**2.00**	**27.70**
**A6**	**40dB nHL**	**Left**	**Insert Earphones**	**0.80**	**Tone Burst**	**4000**	**Alternating**	**Blackman**	**2.00**	**2.00**	**27.70**
**A7**	**34dB nHL**	**Left**	**Insert Earphones**	**0.80**	**Tone Burst**	**4000**	**Alternating**	**Blackman**	**2.00**	**2.00**	**27.70**
**A8**	**33dB nHL**	**Left**	**Insert Earphones**	**0.80**	**Tone Burst**	**4000**	**Alternating**	**Blackman**	**2.00**	**2.00**	**27.70**
**A9**	**32dB nHL**	**Left**	**Insert Earphones**	**0.80**	**Tone Burst**	**4000**	**Alternating**	**Blackman**	**2.00**	**2.00**	**27.70**
**A10**	**32dB nHL**	**Left**	**Insert Earphones**	**0.80**	**Tone Burst**	**4000**	**Alternating**	**Blackman**	**2.00**	**2.00**	**27.70**
**A11**	**31dB nHL**	**Left**	**Insert Earphones**	**0.80**	**Tone Burst**	**4000**	**Alternating**	**Blackman**	**2.00**	**2.00**	**27.70**
**A12**	**31dB nHL**	**Left**	**Insert Earphones**	**0.80**	**Tone Burst**	**4000**	**Alternating**	**Blackman**	**2.00**	**2.00**	**27.70**
**A13**	**30dB nHL**	**Left**	**Insert Earphones**	**0.80**	**Tone Burst**	**4000**	**Alternating**	**Blackman**	**2.00**	**2.00**	**27.70**
**A14**	**30dB nHL**	**Left**	**Insert Earphones**	**0.80**	**Tone Burst**	**4000**	**Alternating**	**Blackman**	**2.00**	**2.00**	**27.70**
**A15**	**30dB nHL**	**Left**	**Insert Earphones**	**0.80**	**Tone Burst**	**4000**	**Alternating**	**Blackman**	**2.00**	**2.00**	**27.70**
**A16**	**29dB nHL**	**Left**	**Insert Earphones**	**0.80**	**Tone Burst**	**4000**	**Alternating**	**Blackman**	**2.00**	**2.00**	**27.70**
**A17**	**28dB nHL**	**Left**	**Insert Earphones**	**0.80**	**Tone Burst**	**4000**	**Alternating**	**Blackman**	**2.00**	**2.00**	**27.70**
**A18**	**27dB nHL**	**Left**	**Insert Earphones**	**0.80**	**Tone Burst**	**4000**	**Alternating**	**Blackman**	**2.00**	**2.00**	**27.70**
**A19**	**26dB nHL**	**Left**	**Insert Earphones**	**0.80**	**Tone Burst**	**4000**	**Alternating**	**Blackman**	**2.00**	**2.00**	**27.70**
**A20**	**25dB nHL**	**Left**	**Insert Earphones**	**0.80**	**Tone Burst**	**4000**	**Alternating**	**Blackman**	**2.00**	**2.00**	**27.70**
**A21**	**25dB nHL**	**Left**	**Insert Earphones**	**0.80**	**Tone Burst**	**4000**	**Alternating**	**Blackman**	**2.00**	**2.00**	**27.70**
**A22**	**24dB nHL**	**Left**	**Insert Earphones**	**0.80**	**Tone Burst**	**4000**	**Alternating**	**Blackman**	**2.00**	**2.00**	**27.70**
**A23**	**23dB nHL**	**Left**	**Insert Earphones**	**0.80**	**Tone Burst**	**4000**	**Alternating**	**Blackman**	**2.00**	**2.00**	**27.70**
**A24**	**22dB nHL**	**Left**	**Insert Earphones**	**0.80**	**Tone Burst**	**4000**	**Alternating**	**Blackman**	**2.00**	**2.00**	**27.70**
**A25**	**21dB nHL**	**Left**	**Insert Earphones**	**0.80**	**Tone Burst**	**4000**	**Alternating**	**Blackman**	**2.00**	**2.00**	**27.70**
**A26**	**20dB nHL**	**Left**	**Insert Earphones**	**0.80**	**Tone Burst**	**4000**	**Alternating**	**Blackman**	**2.00**	**2.00**	**27.70**
**A27**	**20dB nHL**	**Left**	**Insert Earphones**	**0.80**	**Tone Burst**	**4000**	**Alternating**	**Blackman**	**2.00**	**2.00**	**27.70**
**A28**	**19dB nHL**	**Left**	**Insert Earphones**	**0.80**	**Tone Burst**	**4000**	**Alternating**	**Blackman**	**2.00**	**2.00**	**27.70**
**A29**	**18dB nHL**	**Left**	**Insert Earphones**	**0.80**	**Tone Burst**	**4000**	**Alternating**	**Blackman**	**2.00**	**2.00**	**27.70**
**A30**	**17dB nHL**	**Left**	**Insert Earphones**	**0.80**	**Tone Burst**	**4000**	**Alternating**	**Blackman**	**2.00**	**2.00**	**27.70**
**A31**	**17dB nHL**	**Left**	**Insert Earphones**	**0.80**	**Tone Burst**	**4000**	**Alternating**	**Blackman**	**2.00**	**2.00**	**27.70**
**A32**	**16dB nHL**	**Left**	**Insert Earphones**	**0.80**	**Tone Burst**	**4000**	**Alternating**	**Blackman**	**2.00**	**2.00**	**27.70**
**A33**	**16dB nHL**	**Left**	**Insert Earphones**	**0.80**	**Tone Burst**	**4000**	**Alternating**	**Blackman**	**2.00**	**2.00**	**27.70**
**A34**	**15dB nHL**	**Left**	**Insert Earphones**	**0.80**	**Tone Burst**	**4000**	**Alternating**	**Blackman**	**2.00**	**2.00**	**27.70**
**A35**	**14dB nHL**	**Left**	**Insert Earphones**	**0.80**	**Tone Burst**	**4000**	**Alternating**	**Blackman**	**2.00**	**2.00**	**27.70**
**A36**	**13dB nHL**	**Left**	**Insert Earphones**	**0.80**	**Tone Burst**	**4000**	**Alternating**	**Blackman**	**2.00**	**2.00**	**27.70**
**A37**	**12dB nHL**	**Left**	**Insert Earphones**	**0.80**	**Tone Burst**	**4000**	**Alternating**	**Blackman**	**2.00**	**2.00**	**27.70**
**A38**	**11dB nHL**	**Left**	**Insert Earphones**	**0.80**	**Tone Burst**	**4000**	**Alternating**	**Blackman**	**2.00**	**2.00**	**27.70**
**A39**	**11dB nHL**	**Left**	**Insert Earphones**	**0.80**	**Tone Burst**	**4000**	**Alternating**	**Blackman**	**2.00**	**2.00**	**27.70**
**A40**	**10dB nHL**	**Left**	**Insert Earphones**	**0.80**	**Tone Burst**	**4000**	**Alternating**	**Blackman**	**2.00**	**2.00**	**27.70**

A41	9dB nHL	Left	Insert Earphones	0.80	Tone Burst	4000	Alternating	Blackman	2.00	2.00	27.70
A42	8dB nHL	Left	Insert Earphones	0.80	Tone Burst	4000	Alternating	Blackman	2.00	2.00	27.70
A43	7dB nHL	Left	Insert Earphones	0.80	Tone Burst	4000	Alternating	Blackman	2.00	2.00	27.70
A44	6dB nHL	Left	Insert Earphones	0.80	Tone Burst	4000	Alternating	Blackman	2.00	2.00	27.70
A45	5dB nHL	Left	Insert Earphones	0.80	Tone Burst	4000	Alternating	Blackman	2.00	2.00	27.70
A46	5dB nHL	Left	Insert Earphones	0.80	Tone Burst	4000	Alternating	Blackman	2.00	2.00	27.70
A47	4dB nHL	Left	Insert Earphones	0.80	Tone Burst	4000	Alternating	Blackman	2.00	2.00	27.70
A48	3dB nHL	Left	Insert Earphones	0.80	Tone Burst	4000	Alternating	Blackman	2.00	2.00	27.70
A49	2dB nHL	Left	Insert Earphones	0.80	Tone Burst	4000	Alternating	Blackman	2.00	2.00	27.70
A50	1dB nHL	Left	Insert Earphones	0.80	Tone Burst	4000	Alternating	Blackman	2.00	2.00	27.70

Recording Parameters

Label Index	*Epoch*	*Points*	*Pre/Post*	*Averages*	*Artifacts*
A1	16.00	256	0.00	336	13
A2	16.00	256	0.00	542	23
A3	16.00	256	0.00	487	14
A4	16.00	256	0.00	422	13
A5	16.00	256	0.00	682	28
A6	16.00	256	0.00	758	27
A7	16.00	256	0.00	568	12
A8	16.00	256	0.00	635	18
A9	16.00	256	0.00	1018	32
A10	16.00	256	0.00	967	32
A11	16.00	256	0.00	526	23
A12	16.00	256	0.00	820	33
A13	16.00	256	0.00	1058	34
A14	16.00	256	0.00	949	35
A15	16.00	256	0.00	1060	36
A16	16.00	256	0.00	612	18
A17	16.00	256	0.00	612	20
A18	16.00	256	0.00	1189	37
A19	16.00	256	0.00	965	33
A20	16.00	256	0.00	1144	31
A21	16.00	256	0.00	1364	38
A22	16.00	256	0.00	1045	37
A23	16.00	256	0.00	733	24
A24	16.00	256	0.00	539	15
A25	16.00	256	0.00	334	9
A26	16.00	256	0.00	939	26
A27	16.00	256	0.00	848	29
A28	16.00	256	0.00	627	19
A29	16.00	256	0.00	604	20
A30	16.00	256	0.00	485	14

A31	16.00	256	0.00	570	18
A32	16.00	256	0.00	1070	32
A33	16.00	256	0.00	761	28
A34	16.00	256	0.00	697	23
A35	16.00	256	0.00	684	21
A36	16.00	256	0.00	733	24
A37	16.00	256	0.00	618	21
A38	16.00	256	0.00	1428	47
A39	16.00	256	0.00	1237	37
A40	16.00	256	0.00	740	29
A41	16.00	256	0.00	857	28
A42	16.00	256	0.00	983	33
A43	16.00	256	0.00	479	18
A44	16.00	256	0.00	675	23
A45	16.00	256	0.00	1223	40
A46	16.00	256	0.00	1759	46
A47	16.00	256	0.00	1407	47
A48	16.00	256	0.00	1176	32
A49	16.00	256	0.00	1245	45
A50	16.00	256	0.00	802	27

Amplifier Parameters

Label Index	*Channel*	*Gain*	*Low Filter*	*High Filter*	*Notch Filter*	*Artifact Rejection*	*Input 1*	*Input 2*
A1	1	100000	30	1500	No	50.00	FZ	A1A2
A2	1	100000	30	1500	No	50.00	FZ	A1A2
A3	1	100000	30	1500	No	50.00	FZ	A1A2
A4	1	100000	30	1500	No	50.00	FZ	A1A2
A5	1	100000	30	1500	No	50.00	FZ	A1A2
A6	1	100000	30	1500	No	50.00	FZ	A1A2
A7	1	100000	30	1500	No	50.00	FZ	A1A2
A8	1	100000	30	1500	No	50.00	FZ	A1A2
A9	1	100000	30	1500	No	50.00	FZ	A1A2
A10	1	100000	30	1500	No	50.00	FZ	A1A2
A11	1	100000	30	1500	No	50.00	FZ	A1A2
A12	1	100000	30	1500	No	50.00	FZ	A1A2
A13	1	100000	30	1500	No	50.00	FZ	A1A2
A14	1	100000	30	1500	No	50.00	FZ	A1A2
A15	1	100000	30	1500	No	50.00	FZ	A1A2
A16	1	100000	30	1500	No	50.00	FZ	A1A2
A17	1	100000	30	1500	No	50.00	FZ	A1A2
A18	1	100000	30	1500	No	50.00	FZ	A1A2
A19	1	100000	30	1500	No	50.00	FZ	A1A2
A20	1	100000	30	1500	No	50.00	FZ	A1A2

A21	1	100000	30	1500	No	50.00	FZ	A1A2
A22	1	100000	30	1500	No	50.00	FZ	A1A2
A23	1	100000	30	1500	No	50.00	FZ	A1A2
A24	1	100000	30	1500	No	50.00	FZ	A1A2
A25	1	100000	30	1500	No	50.00	FZ	A1A2
A26	1	100000	30	1500	No	50.00	FZ	A1A2
A27	1	100000	30	1500	No	50.00	FZ	A1A2
A28	1	100000	30	1500	No	50.00	FZ	A1A2
A29	1	100000	30	1500	No	50.00	FZ	A1A2
A30	1	100000	30	1500	No	50.00	FZ	A1A2
A31	1	100000	30	1500	No	50.00	FZ	A1A2
A32	1	100000	30	1500	No	50.00	FZ	A1A2
A33	1	100000	30	1500	No	50.00	FZ	A1A2
A34	1	100000	30	1500	No	50.00	FZ	A1A2
A35	1	100000	30	1500	No	50.00	FZ	A1A2
A36	1	100000	30	1500	No	50.00	FZ	A1A2
A37	1	100000	30	1500	No	50.00	FZ	A1A2
A38	1	100000	30	1500	No	50.00	FZ	A1A2
A39	1	100000	30	1500	No	50.00	FZ	A1A2
A40	1	100000	30	1500	No	50.00	FZ	A1A2
A41	1	100000	30	1500	No	50.00	FZ	A1A2
A42	1	100000	30	1500	No	50.00	FZ	A1A2
A43	1	100000	30	1500	No	50.00	FZ	A1A2
A44	1	100000	30	1500	No	50.00	FZ	A1A2
A45	1	100000	30	1500	No	50.00	FZ	A1A2
A46	1	100000	30	1500	No	50.00	FZ	A1A2
A47	1	100000	30	1500	No	50.00	FZ	A1A2
A48	1	100000	30	1500	No	50.00	FZ	A1A2
A49	1	100000	30	1500	No	50.00	FZ	A1A2
A50	1	100000	30	1500	No	50.00	FZ	A1A2
