# Supplementary material for: Auditory brainstem responses in the nine-banded armadillo (Dasypus novemcinctus)
Source: PeerJ. 2023 Dec 13;11:e16602. doi: 10.7717/peerj.16602 (PMC10725177; doi:10.7717/peerj.16602)
Supplement: Supplemental Information 2 — Each raw data file shows ABR amplitude (blue line) across various stimulus intensities (indicated on y-axis) over time in milliseconds (indicated on x-axis) for a particular experiment. [file peerj-11-16602-s002.zip › Armadillo 2021/#2 Animal F14-02 Case 15-04/2000 Hz.pdf]

EVOKED POTENTIAL REPORT

UAMS CHP Speech and Hearing Clinic
Department of Audiology and Speech Pathology
4021 W. 8th Street
Little Rock, AR 72204
(501) 320-7300

Patient:	**Case 15-04 (f1402), Armadillo**
ID#:	**Armadillo f1402**
Gender:	
Birth date:	**02/09/15**

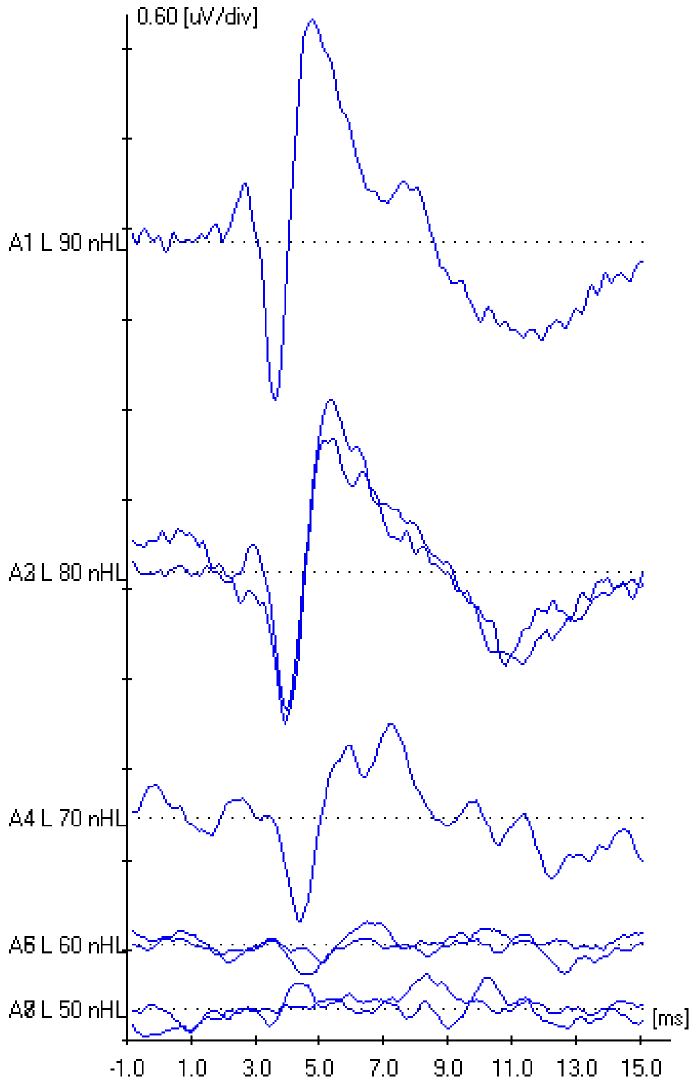

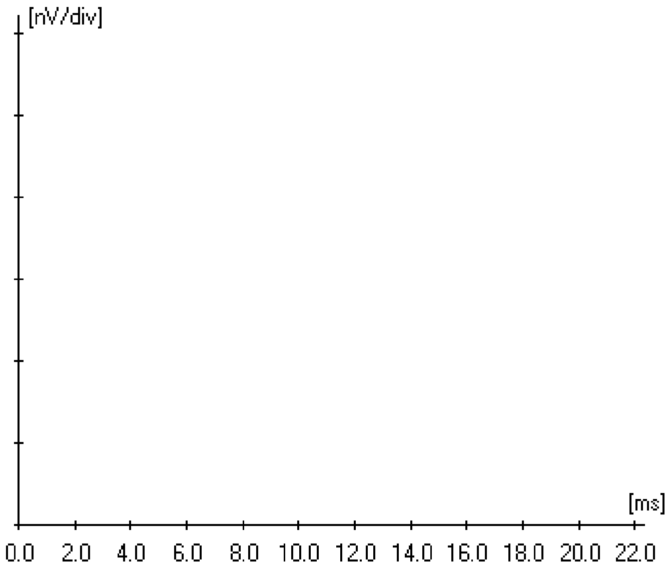

Latencies (ms)

Label Index	*I*	*II*	*III*	*IV*	*V*

Interlatencies (ms)

Label Index	*I-III*	*III-V*	*I-V*

Interaural Latency Differences

Label Index	*L1*	*L2*	*L3*	*L4*	*L5*	*L6*	*L7*	*L8*	*L9*	*L10*

Stimulus Parameters

Label Index	*Intensity*	*Ear*	*Transducer*	*Insert Delay*	*Type*	*Frequency*	*Polarity*	*Ramp*	*Rise/Fall*	*Plateau*	*Rate*
**A1**	**90dB nHL**	**Left**	**Insert Earphones**	**0.80**	**Tone Burst**	**2000**	**Alternating**	**Blackman**	**2.00**	**2.00**	**27.70**
**A2**	**80dB nHL**	**Left**	**Insert Earphones**	**0.80**	**Tone Burst**	**2000**	**Alternating**	**Blackman**	**2.00**	**2.00**	**27.70**
**A3**	**80dB nHL**	**Left**	**Insert Earphones**	**0.80**	**Tone Burst**	**2000**	**Alternating**	**Blackman**	**2.00**	**2.00**	**27.70**
**A4**	**70dB nHL**	**Left**	**Insert Earphones**	**0.80**	**Tone Burst**	**2000**	**Alternating**	**Blackman**	**2.00**	**2.00**	**27.70**
**A5**	**60dB nHL**	**Left**	**Insert Earphones**	**0.80**	**Tone Burst**	**2000**	**Alternating**	**Blackman**	**2.00**	**2.00**	**27.70**
**A6**	**60dB nHL**	**Left**	**Insert Earphones**	**0.80**	**Tone Burst**	**2000**	**Alternating**	**Blackman**	**2.00**	**2.00**	**27.70**
**A7**	**50dB nHL**	**Left**	**Insert Earphones**	**0.80**	**Tone Burst**	**2000**	**Alternating**	**Blackman**	**2.00**	**2.00**	**27.70**
**A8**	**50dB nHL**	**Left**	**Insert Earphones**	**0.80**	**Tone Burst**	**2000**	**Alternating**	**Blackman**	**2.00**	**2.00**	**27.70**

Recording Parameters

Label Index	*Epoch*	*Points*	*Pre/Post*	*Averages*	*Artifacts*
**A1**	**16.00**	**256**	**0.00**	**1067**	**14**
**A2**	**16.00**	**256**	**0.00**	**1757**	**197**
**A3**	**16.00**	**256**	**0.00**	**711**	**5**
**A4**	**16.00**	**256**	**0.00**	**727**	**4**
**A5**	**16.00**	**256**	**0.00**	**1288**	**4**
**A6**	**16.00**	**256**	**0.00**	**2285**	**4**
**A7**	**16.00**	**256**	**0.00**	**1540**	**4**
**A8**	**16.00**	**256**	**0.00**	**942**	**3**

Amplifier Parameters

Label Index	*Channel*	*Gain*	*Low Filter*	*High Filter*	*Notch Filter*	*Artifact Rejection*	*Input 1*	*Input 2*
**A1**	**1**	**100000**	**30**	**1500**	**No**	**50.00**	**FZ**	**A1A2**
**A2**	**1**	**100000**	**30**	**1500**	**No**	**50.00**	**FZ**	**A1A2**
**A3**	**1**	**100000**	**30**	**1500**	**No**	**50.00**	**FZ**	**A1A2**
**A4**	**1**	**100000**	**30**	**1500**	**No**	**50.00**	**FZ**	**A1A2**
**A5**	**1**	**100000**	**30**	**1500**	**No**	**50.00**	**FZ**	**A1A2**
**A6**	**1**	**100000**	**30**	**1500**	**No**	**50.00**	**FZ**	**A1A2**
**A7**	**1**	**100000**	**30**	**1500**	**No**	**50.00**	**FZ**	**A1A2**
**A8**	**1**	**100000**	**30**	**1500**	**No**	**50.00**	**FZ**	**A1A2**
