# Supplementary material for: Auditory brainstem responses in the nine-banded armadillo (Dasypus novemcinctus)
Source: PeerJ. 2023 Dec 13;11:e16602. doi: 10.7717/peerj.16602 (PMC10725177; doi:10.7717/peerj.16602)
Supplement: Supplemental Information 2 — Each raw data file shows ABR amplitude (blue line) across various stimulus intensities (indicated on y-axis) over time in milliseconds (indicated on x-axis) for a particular experiment. [file peerj-11-16602-s002.zip › Armadillo 2021/#2 Animal F14-02 Case 15-04/All other frequencies by record number.pdf]

EVOKED POTENTIAL REPORT

UAMS CHP Speech and Hearing Clinic
Department of Audiology and Speech Pathology
4021 W. 8th Street
Little Rock, AR 72204
(501) 320-7300

Patient: **Case 15-04 (f1402), Armadillo**

ID#: **Armadillo f1402**

Gender:

Birth date: **02/09/15**

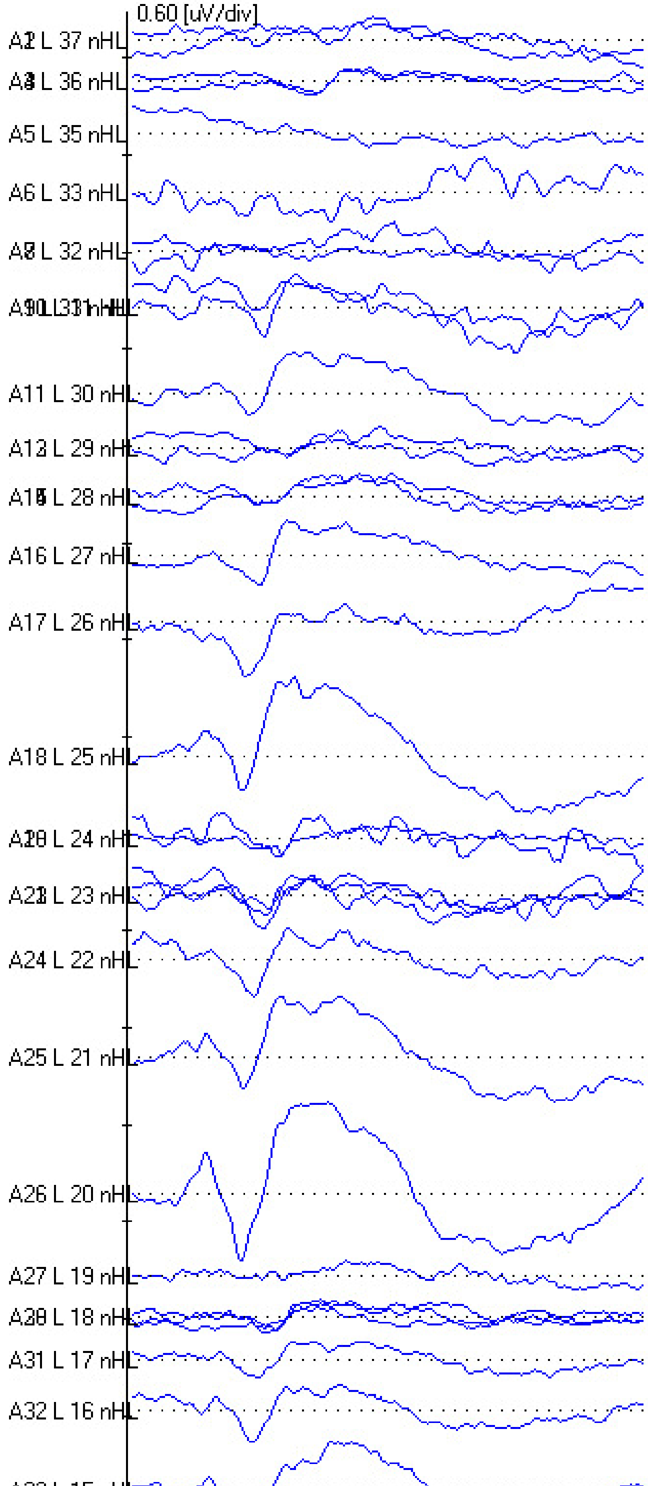

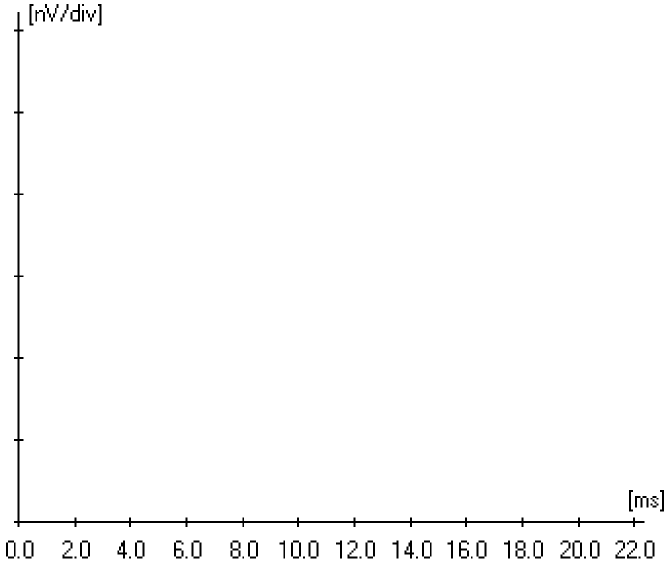

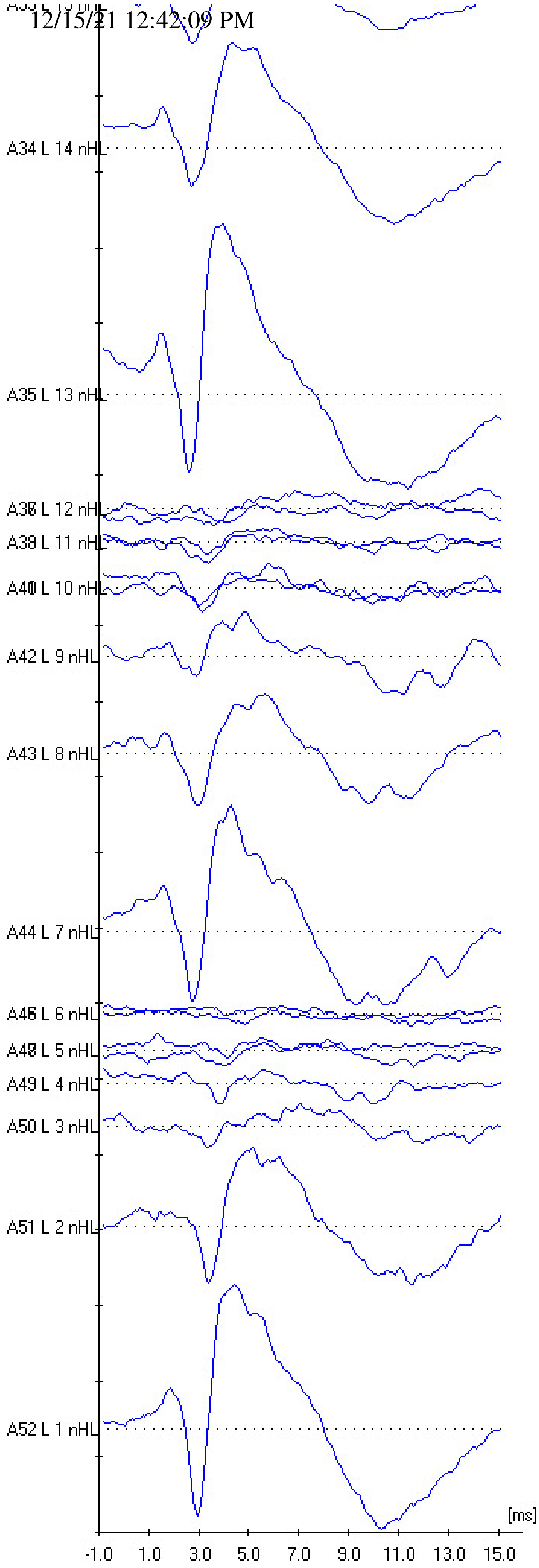
A34 L 14 nHL
A35 L 13 nHL
L 12 nHL
L 11 nHL
L 10 nHL
A42 L 9 nHL
A43 L 8 nHL
A44 L 7 nHL
L 6 nHL
L 5 nHL
A49 L 4 nHL
A50 L 3 nHL
A51 L 2 nHL
A52 L 1 nHL
[ms]
-1.0
1.0
3.0
5.0
7.0
9.0
11.0
13.0
15.0

Latencies (ms)

Label Index	*I*	*II*	*III*	*IV*	*V*

Interlatencies (ms)

Label Index	*I-III*	*III-V*	*I-V*

Interaural Latency Differences

Label Index	*L1*	*L2*	*L3*	*L4*	*L5*	*L6*	*L7*	*L8*	*L9*	*L10*

Stimulus Parameters

Label Index	*Intensity*	*Ear*	*Transducer*	*Insert Delay*	*Type*	*Frequency*	*Polarity*	*Ramp*	*Rise/Fall*	*Plateau*	*Rate*
**A1**	**37dB nHL**	**Left**	**Insert Earphones**	**0.80**	**Tone Burst**	**2000**	**Alternating**	**Blackman**	**2.00**	**2.00**	**27.70**
**A2**	**37dB nHL**	**Left**	**Insert Earphones**	**0.80**	**Tone Burst**	**2000**	**Alternating**	**Blackman**	**2.00**	**2.00**	**27.70**
**A3**	**36dB nHL**	**Left**	**Insert Earphones**	**0.80**	**Tone Burst**	**2000**	**Alternating**	**Blackman**	**2.00**	**2.00**	**27.70**
**A4**	**36dB nHL**	**Left**	**Insert Earphones**	**0.80**	**Tone Burst**	**2000**	**Alternating**	**Blackman**	**2.00**	**2.00**	**27.70**
**A5**	**35dB nHL**	**Left**	**Insert Earphones**	**0.80**	**Tone Burst**	**2000**	**Alternating**	**Blackman**	**2.00**	**2.00**	**27.70**
**A6**	**33dB nHL**	**Left**	**Insert Earphones**	**0.80**	**Tone Burst**	**2000**	**Alternating**	**Blackman**	**2.00**	**2.00**	**27.70**
**A7**	**32dB nHL**	**Left**	**Insert Earphones**	**0.80**	**Tone Burst**	**2000**	**Alternating**	**Blackman**	**2.00**	**2.00**	**27.70**
**A8**	**32dB nHL**	**Left**	**Insert Earphones**	**0.80**	**Tone Burst**	**2000**	**Alternating**	**Blackman**	**2.00**	**2.00**	**27.70**
**A9**	**31dB nHL**	**Left**	**Insert Earphones**	**0.80**	**Tone Burst**	**2000**	**Alternating**	**Blackman**	**2.00**	**2.00**	**27.70**
**A10**	**31dB nHL**	**Left**	**Insert Earphones**	**0.80**	**Tone Burst**	**2000**	**Alternating**	**Blackman**	**2.00**	**2.00**	**27.70**
**A11**	**30dB nHL**	**Left**	**Insert Earphones**	**0.80**	**Tone Burst**	**2000**	**Alternating**	**Blackman**	**2.00**	**2.00**	**27.70**
**A12**	**29dB nHL**	**Left**	**Insert Earphones**	**0.80**	**Tone Burst**	**2000**	**Alternating**	**Blackman**	**2.00**	**2.00**	**27.70**
**A13**	**29dB nHL**	**Left**	**Insert Earphones**	**0.80**	**Tone Burst**	**2000**	**Alternating**	**Blackman**	**2.00**	**2.00**	**27.70**
**A14**	**28dB nHL**	**Left**	**Insert Earphones**	**0.80**	**Tone Burst**	**2000**	**Alternating**	**Blackman**	**2.00**	**2.00**	**27.70**
**A15**	**28dB nHL**	**Left**	**Insert Earphones**	**0.80**	**Tone Burst**	**2000**	**Alternating**	**Blackman**	**2.00**	**2.00**	**27.70**
**A16**	**27dB nHL**	**Left**	**Insert Earphones**	**0.80**	**Tone Burst**	**2000**	**Alternating**	**Blackman**	**2.00**	**2.00**	**27.70**
**A17**	**26dB nHL**	**Left**	**Insert Earphones**	**0.80**	**Tone Burst**	**2000**	**Alternating**	**Blackman**	**2.00**	**2.00**	**27.70**
**A18**	**25dB nHL**	**Left**	**Insert Earphones**	**0.80**	**Tone Burst**	**2000**	**Alternating**	**Blackman**	**2.00**	**2.00**	**27.70**
**A19**	**24dB nHL**	**Left**	**Insert Earphones**	**0.80**	**Tone Burst**	**2000**	**Alternating**	**Blackman**	**2.00**	**2.00**	**27.70**
**A20**	**24dB nHL**	**Left**	**Insert Earphones**	**0.80**	**Tone Burst**	**2000**	**Alternating**	**Blackman**	**2.00**	**2.00**	**27.70**
**A21**	**23dB nHL**	**Left**	**Insert Earphones**	**0.80**	**Tone Burst**	**2000**	**Alternating**	**Blackman**	**2.00**	**2.00**	**27.70**
**A22**	**23dB nHL**	**Left**	**Insert Earphones**	**0.80**	**Tone Burst**	**2000**	**Alternating**	**Blackman**	**2.00**	**2.00**	**27.70**
**A23**	**23dB nHL**	**Left**	**Insert Earphones**	**0.80**	**Tone Burst**	**2000**	**Alternating**	**Blackman**	**2.00**	**2.00**	**27.70**
**A24**	**22dB nHL**	**Left**	**Insert Earphones**	**0.80**	**Tone Burst**	**2000**	**Alternating**	**Blackman**	**2.00**	**2.00**	**27.70**
**A25**	**21dB nHL**	**Left**	**Insert Earphones**	**0.80**	**Tone Burst**	**2000**	**Alternating**	**Blackman**	**2.00**	**2.00**	**27.70**
**A26**	**20dB nHL**	**Left**	**Insert Earphones**	**0.80**	**Tone Burst**	**2000**	**Alternating**	**Blackman**	**2.00**	**2.00**	**27.70**
**A27**	**19dB nHL**	**Left**	**Insert Earphones**	**0.80**	**Tone Burst**	**2000**	**Alternating**	**Blackman**	**2.00**	**2.00**	**27.70**
**A28**	**18dB nHL**	**Left**	**Insert Earphones**	**0.80**	**Tone Burst**	**2000**	**Alternating**	**Blackman**	**2.00**	**2.00**	**27.70**
**A29**	**18dB nHL**	**Left**	**Insert Earphones**	**0.80**	**Tone Burst**	**2000**	**Alternating**	**Blackman**	**2.00**	**2.00**	**27.70**
**A30**	**18dB nHL**	**Left**	**Insert Earphones**	**0.80**	**Tone Burst**	**2000**	**Alternating**	**Blackman**	**2.00**	**2.00**	**27.70**
**A31**	**17dB nHL**	**Left**	**Insert Earphones**	**0.80**	**Tone Burst**	**2000**	**Alternating**	**Blackman**	**2.00**	**2.00**	**27.70**

A32	16dB nHL	Left	Insert Earphones	0.80	Tone Burst	2000	Alternating	Blackman	2.00	2.00	27.70
A33	15dB nHL	Left	Insert Earphones	0.80	Tone Burst	2000	Alternating	Blackman	2.00	2.00	27.70
A34	14dB nHL	Left	Insert Earphones	0.80	Tone Burst	2000	Alternating	Blackman	2.00	2.00	27.70
A35	13dB nHL	Left	Insert Earphones	0.80	Tone Burst	2000	Alternating	Blackman	2.00	2.00	27.70
A36	12dB nHL	Left	Insert Earphones	0.80	Tone Burst	2000	Alternating	Blackman	2.00	2.00	27.70
A37	12dB nHL	Left	Insert Earphones	0.80	Tone Burst	2000	Alternating	Blackman	2.00	2.00	27.70
A38	11dB nHL	Left	Insert Earphones	0.80	Tone Burst	2000	Alternating	Blackman	2.00	2.00	27.70
A39	11dB nHL	Left	Insert Earphones	0.80	Tone Burst	2000	Alternating	Blackman	2.00	2.00	27.70
A40	10dB nHL	Left	Insert Earphones	0.80	Tone Burst	2000	Alternating	Blackman	2.00	2.00	27.70
A41	10dB nHL	Left	Insert Earphones	0.80	Tone Burst	2000	Alternating	Blackman	2.00	2.00	27.70
A42	9dB nHL	Left	Insert Earphones	0.80	Tone Burst	2000	Alternating	Blackman	2.00	2.00	27.70
A43	8dB nHL	Left	Insert Earphones	0.80	Tone Burst	2000	Alternating	Blackman	2.00	2.00	27.70
A44	7dB nHL	Left	Insert Earphones	0.80	Tone Burst	2000	Alternating	Blackman	2.00	2.00	27.70
A45	6dB nHL	Left	Insert Earphones	0.80	Tone Burst	2000	Alternating	Blackman	2.00	2.00	27.70
A46	6dB nHL	Left	Insert Earphones	0.80	Tone Burst	2000	Alternating	Blackman	2.00	2.00	27.70
A47	5dB nHL	Left	Insert Earphones	0.80	Tone Burst	2000	Alternating	Blackman	2.00	2.00	27.70
A48	5dB nHL	Left	Insert Earphones	0.80	Tone Burst	2000	Alternating	Blackman	2.00	2.00	27.70
A49	4dB nHL	Left	Insert Earphones	0.80	Tone Burst	2000	Alternating	Blackman	2.00	2.00	27.70
A50	3dB nHL	Left	Insert Earphones	0.80	Tone Burst	2000	Alternating	Blackman	2.00	2.00	27.70
A51	2dB nHL	Left	Insert Earphones	0.80	Tone Burst	2000	Alternating	Blackman	2.00	2.00	27.70
A52	1dB nHL	Left	Insert Earphones	0.80	Tone Burst	2000	Alternating	Blackman	2.00	2.00	27.70

Recording Parameters

Label Index	*Epoch*	*Points*	*Pre/Post*	*Averages*	*Artifacts*
A1	16.00	256	0.00	1520	6
A2	16.00	256	0.00	899	4
A3	16.00	256	0.00	1474	5
A4	16.00	256	0.00	2946	2
A5	16.00	256	0.00	1353	4
A6	16.00	256	0.00	668	4
A7	16.00	256	0.00	1931	3
A8	16.00	256	0.00	1340	4
A9	16.00	256	0.00	1571	3
A10	16.00	256	0.00	1985	5
A11	16.00	256	0.00	1201	2
A12	16.00	256	0.00	1578	3
A13	16.00	256	0.00	1117	2
A14	16.00	256	0.00	1726	2
A15	16.00	256	0.00	2472	5
A16	16.00	256	0.00	1424	5
A17	16.00	256	0.00	1009	4
A18	16.00	256	0.00	1106	3
A19	16.00	256	0.00	1517	4

[illegible]	16.00	256	0.00	2480	5
A21	16.00	256	0.00	1442	2
A22	16.00	256	0.00	2364	4
A23	16.00	256	0.00	1617	4
A24	16.00	256	0.00	995	6
A25	16.00	256	0.00	1020	2
A26	16.00	256	0.00	853	3
A27	16.00	256	0.00	1085	4
A28	16.00	256	0.00	1858	3
A29	16.00	256	0.00	2769	4
A30	16.00	256	0.00	2119	5
A31	16.00	256	0.00	1626	4
A32	16.00	256	0.00	1170	9
A33	16.00	256	0.00	1019	4
A34	16.00	256	0.00	1558	4
A35	16.00	256	0.00	708	3
A36	16.00	256	0.00	1708	4
A37	16.00	256	0.00	912	3
A38	16.00	256	0.00	1960	7
A39	16.00	256	0.00	2296	3
A40	16.00	256	0.00	1360	5
A41	16.00	256	0.00	1110	5
A42	16.00	256	0.00	1003	4
A43	16.00	256	0.00	1385	3
A44	16.00	256	0.00	889	1
A45	16.00	256	0.00	2543	5
A46	16.00	256	0.00	2272	5
A47	16.00	256	0.00	1464	6
A48	16.00	256	0.00	1736	5
A49	16.00	256	0.00	1651	5
A50	16.00	256	0.00	1498	3
A51	16.00	256	0.00	720	5
A52	16.00	256	0.00	1419	5

Amplifier Parameters

Label Index	*Channel*	*Gain*	*Low Filter*	*High Filter*	*Notch Filter*	*Artifact Rejection*	*Input 1*	*Input 2*
A1	1	100000	30	1500	No	50.00	FZ	A1A2
A2	1	100000	30	1500	No	50.00	FZ	A1A2
A3	1	100000	30	1500	No	50.00	FZ	A1A2
A4	1	100000	30	1500	No	50.00	FZ	A1A2
A5	1	100000	30	1500	No	50.00	FZ	A1A2
A6	1	100000	30	1500	No	50.00	FZ	A1A2
A7	1	100000	30	1500	No	50.00	FZ	A1A2

A8	1	100000	30	1500	No	50.00	FZ	A1A2
A9	1	100000	30	1500	No	50.00	FZ	A1A2
A10	1	100000	30	1500	No	50.00	FZ	A1A2
A11	1	100000	30	1500	No	50.00	FZ	A1A2
A12	1	100000	30	1500	No	50.00	FZ	A1A2
A13	1	100000	30	1500	No	50.00	FZ	A1A2
A14	1	100000	30	1500	No	50.00	FZ	A1A2
A15	1	100000	30	1500	No	50.00	FZ	A1A2
A16	1	100000	30	1500	No	50.00	FZ	A1A2
A17	1	100000	30	1500	No	50.00	FZ	A1A2
A18	1	100000	30	1500	No	50.00	FZ	A1A2
A19	1	100000	30	1500	No	50.00	FZ	A1A2
A20	1	100000	30	1500	No	50.00	FZ	A1A2
A21	1	100000	30	1500	No	50.00	FZ	A1A2
A22	1	100000	30	1500	No	50.00	FZ	A1A2
A23	1	100000	30	1500	No	50.00	FZ	A1A2
A24	1	100000	30	1500	No	50.00	FZ	A1A2
A25	1	100000	30	1500	No	50.00	FZ	A1A2
A26	1	100000	30	1500	No	50.00	FZ	A1A2
A27	1	100000	30	1500	No	50.00	FZ	A1A2
A28	1	100000	30	1500	No	50.00	FZ	A1A2
A29	1	100000	30	1500	No	50.00	FZ	A1A2
A30	1	100000	30	1500	No	50.00	FZ	A1A2
A31	1	100000	30	1500	No	50.00	FZ	A1A2
A32	1	100000	30	1500	No	50.00	FZ	A1A2
A33	1	100000	30	1500	No	50.00	FZ	A1A2
A34	1	100000	30	1500	No	50.00	FZ	A1A2
A35	1	100000	30	1500	No	50.00	FZ	A1A2
A36	1	100000	30	1500	No	50.00	FZ	A1A2
A37	1	100000	30	1500	No	50.00	FZ	A1A2
A38	1	100000	30	1500	No	50.00	FZ	A1A2
A39	1	100000	30	1500	No	50.00	FZ	A1A2
A40	1	100000	30	1500	No	50.00	FZ	A1A2
A41	1	100000	30	1500	No	50.00	FZ	A1A2
A42	1	100000	30	1500	No	50.00	FZ	A1A2
A43	1	100000	30	1500	No	50.00	FZ	A1A2
A44	1	100000	30	1500	No	50.00	FZ	A1A2
A45	1	100000	30	1500	No	50.00	FZ	A1A2
A46	1	100000	30	1500	No	50.00	FZ	A1A2
A47	1	100000	30	1500	No	50.00	FZ	A1A2
A48	1	100000	30	1500	No	50.00	FZ	A1A2
A49	1	100000	30	1500	No	50.00	FZ	A1A2

A5[illegible]	1	100000	30	1500	No	50.00	FZ	A1A2
A51	1	100000	30	1500	No	50.00	FZ	A1A2
A52	1	100000	30	1500	No	50.00	FZ	A1A2
