# Supplementary material for: Auditory brainstem responses in the nine-banded armadillo (Dasypus novemcinctus)
Source: PeerJ. 2023 Dec 13;11:e16602. doi: 10.7717/peerj.16602 (PMC10725177; doi:10.7717/peerj.16602)
Supplement: Supplemental Information 2 — Each raw data file shows ABR amplitude (blue line) across various stimulus intensities (indicated on y-axis) over time in milliseconds (indicated on x-axis) for a particular experiment. [file peerj-11-16602-s002.zip › Armadillo 2021/#1 Animal F14-03 Case 15-05/500 Hz.pdf]

# EVOKED POTENTIAL REPORT

UAMS CHP Speech and Hearing Clinic
Department of Audiology and Speech Pathology
4021 W. 8th Street
Little Rock, AR 72204
(501) 320-7300

| | |
|---|---|
| *Patient:* | **armadillo n14-03, armadillo** |
| *ID#:* | **Armadillo 1505** |
| *Gender:* | |
| *Birth date:* | **02/10/15** |

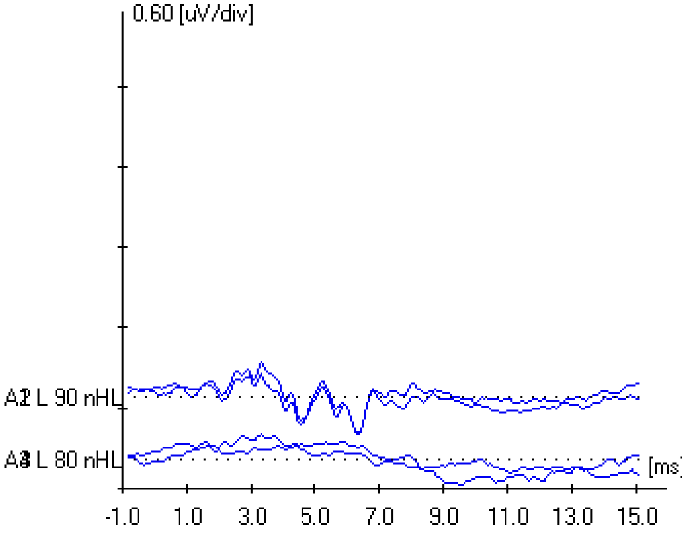


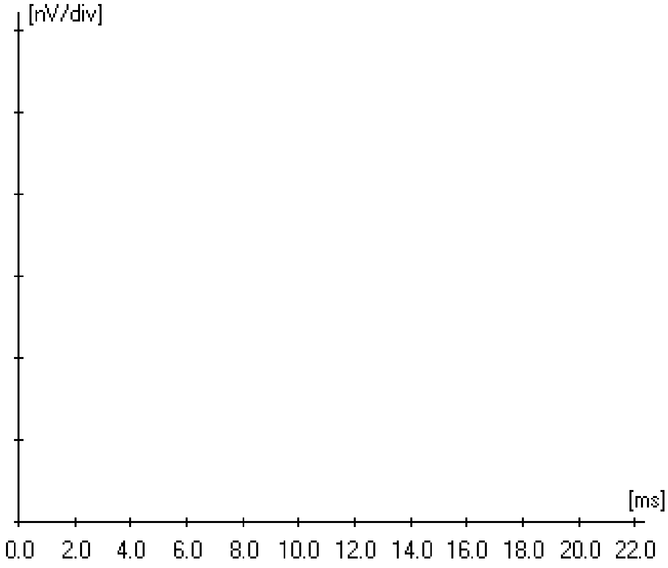


## *Latencies (ms)*

| *Label Index* | *I* | *II* | *III* | *IV* | *V* |
|---|---|---|---|---|---|

## *Interlatencies (ms)*

| *Label Index* | *I-III* | *III-V* | *I-V* |
|---|---|---|---|

## *Interaural Latency Differences*

| *Label Index* | *L1* | *L2* | *L3* | *L4* | *L5* | *L6* | *L7* | *L8* | *L9* | *L10* |
|---|---|---|---|---|---|---|---|---|---|---|

## *Stimulus Parameters*

| *Label Index* | *Intensity* | *Ear* | *Transducer* | *Insert Delay* | *Type* | *Frequency* | *Polarity* | *Ramp* | *Rise/Fall* | *Plateau* | *Rate* |
|---|---|---|---|---|---|---|---|---|---|---|---|
| **A1** | **90dB nHL** | **Left** | **Insert Earphones** | **0.80** | **Tone Burst** | **500** | **Alternating** | **Blackman** | **2.00** | **2.00** | **27.70** |
| **A2** | **90dB nHL** | **Left** | **Insert Earphones** | **0.80** | **Tone Burst** | **500** | **Alternating** | **Blackman** | **2.00** | **2.00** | **27.70** |
| **A3** | **80dB nHL** | **Left** | **Insert Earphones** | **0.80** | **Tone Burst** | **500** | **Alternating** | **Blackman** | **2.00** | **2.00** | **27.70** |
| **A4** | **80dB nHL** | **Left** | **Insert Earphones** | **0.80** | **Tone Burst** | **500** | **Alternating** | **Blackman** | **2.00** | **2.00** | **27.70** |

## *Recording Parameters*

| *Label Index* | *Epoch* | *Points* | *Pre/Post* | *Averages* | *Artifacts* |
|---|---|---|---|---|---|
| **A1** | **16.00** | **256** | **0.00** | **986** | **9** |
| **A2** | **16.00** | **256** | **0.00** | **1580** | **12** |

| | | | | | |
|---|---|---|---|---|---|
| **A3** | **16.00** | **256** | **0.00** | **775** | **7** |
| **A4** | **16.00** | **256** | **0.00** | **1139** | **7** |

## *Amplifier Parameters*

| *Label Index* | *Channel* | *Gain* | *Low Filter* | *High Filter* | *Notch Filter* | *Artifact Rejection* | *Input 1* | *Input 2* |
|---|---|---|---|---|---|---|---|---|
| **A1** | **1** | **100000** | **30** | **1500** | **No** | **50.00** | **FZ** | **A1A2** |
| **A2** | **1** | **100000** | **30** | **1500** | **No** | **50.00** | **FZ** | **A1A2** |
| **A3** | **1** | **100000** | **30** | **1500** | **No** | **50.00** | **FZ** | **A1A2** |
| **A4** | **1** | **100000** | **30** | **1500** | **No** | **50.00** | **FZ** | **A1A2** |
