# Supplementary material for: Auditory brainstem responses in the nine-banded armadillo (Dasypus novemcinctus)
Source: PeerJ. 2023 Dec 13;11:e16602. doi: 10.7717/peerj.16602 (PMC10725177; doi:10.7717/peerj.16602)
Supplement: Supplemental Information 2 — Each raw data file shows ABR amplitude (blue line) across various stimulus intensities (indicated on y-axis) over time in milliseconds (indicated on x-axis) for a particular experiment. [file peerj-11-16602-s002.zip › Armadillo 2021/#1 Animal F14-03 Case 15-05/All other frequencies by record number.pdf]

EVOKED POTENTIAL REPORT

UAMS CHP Speech and Hearing Clinic
Department of Audiology and Speech Pathology
4021 W. 8th Street
Little Rock, AR 72204
(501) 320-7300

Patient:	**armadillo n14-03, armadillo**
ID#:	**Armadillo 1505**
Gender:	
Birth date:	**02/10/15**

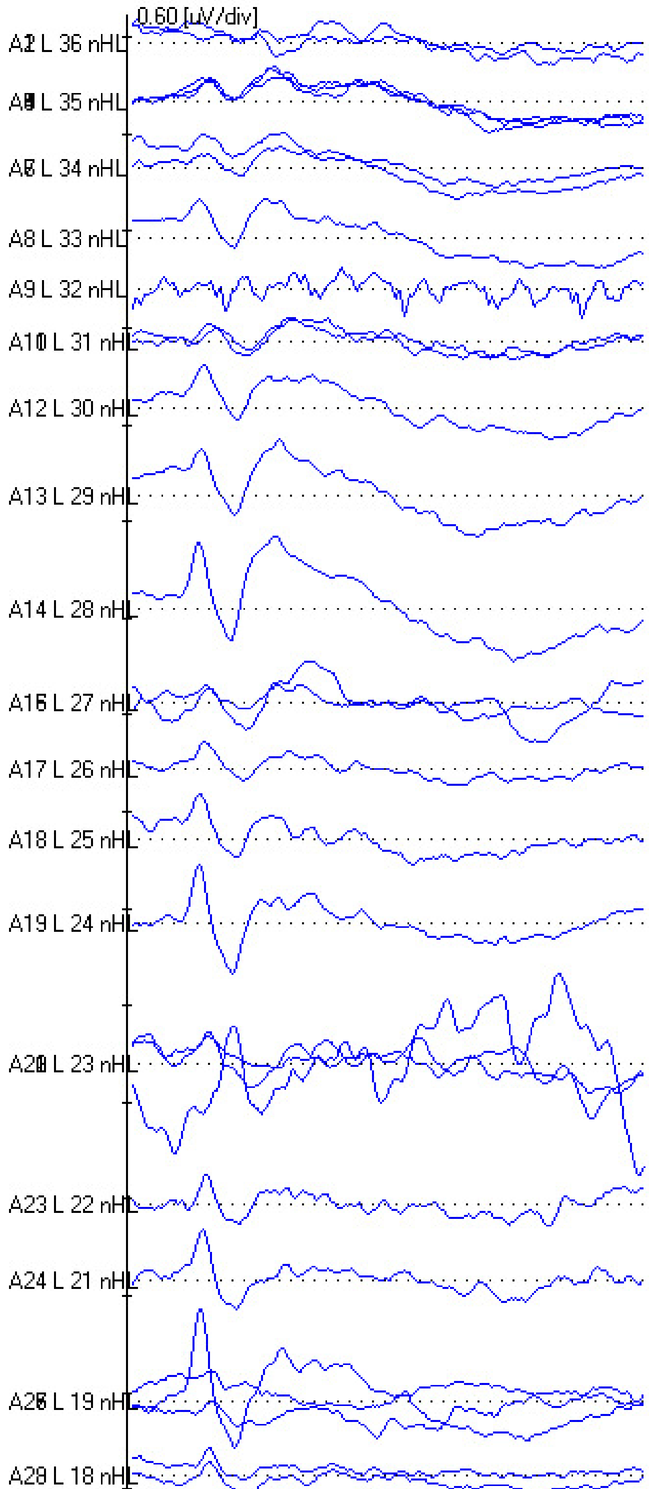

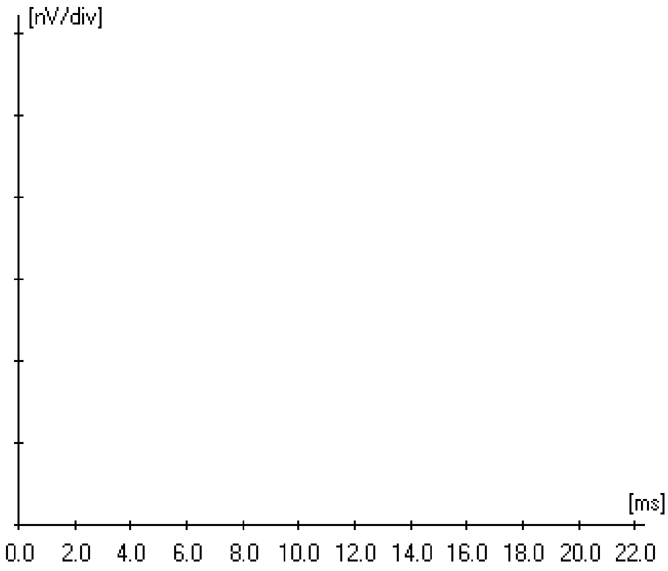

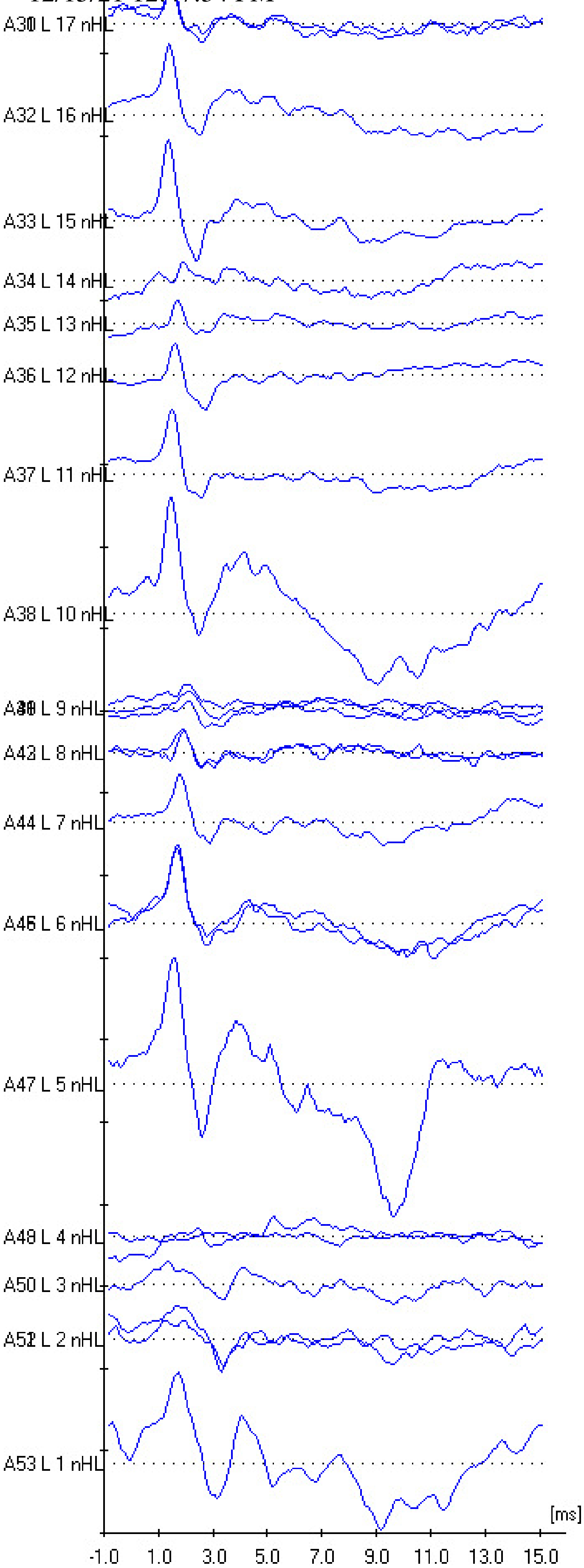
L 17 nHL
A32 L 16 nHL
A33 L 15 nHL
A34 L 14 nHL
A35 L 13 nHL
A36 L 12 nHL
A37 L 11 nHL
A38 L 10 nHL
L 9 nHL
L 8 nHL
A44 L 7 nHL
L 6 nHL
A47 L 5 nHL
L 4 nHL
A50 L 3 nHL
L 2 nHL
A53 L 1 nHL
[ms]
-1.0
1.0
3.0
5.0
7.0
9.0
11.0
13.0
15.0

Latencies (ms)

Label Index	*I*	*II*	*III*	*IV*	*V*

Interlatencies (ms)

Label Index	*I-III*	*III-V*	*I-V*

Interaural Latency Differences

Label Index	*L1*	*L2*	*L3*	*L4*	*L5*	*L6*	*L7*	*L8*	*L9*	*L10*

Stimulus Parameters

Label Index	*Intensity*	*Ear*	*Transducer*	*Insert Delay*	*Type*	*Frequency*	*Polarity*	*Ramp*	*Rise/Fall*	*Plateau*	*Rate*
**A1**	**36dB nHL**	**Left**	**Insert Earphones**	**0.80**	**Tone Burst**	**2000**	**Alternating**	**Blackman**	**2.00**	**2.00**	**27.70**
**A2**	**36dB nHL**	**Left**	**Insert Earphones**	**0.80**	**Tone Burst**	**2000**	**Alternating**	**Blackman**	**2.00**	**2.00**	**27.70**
**A3**	**35dB nHL**	**Left**	**Insert Earphones**	**0.80**	**Tone Burst**	**2000**	**Alternating**	**Blackman**	**2.00**	**2.00**	**27.70**
**A4**	**35dB nHL**	**Left**	**Insert Earphones**	**0.80**	**Tone Burst**	**2000**	**Alternating**	**Blackman**	**2.00**	**2.00**	**27.70**
**A5**	**35dB nHL**	**Left**	**Insert Earphones**	**0.80**	**Tone Burst**	**2000**	**Alternating**	**Blackman**	**2.00**	**2.00**	**27.70**
**A6**	**34dB nHL**	**Left**	**Insert Earphones**	**0.80**	**Tone Burst**	**2000**	**Alternating**	**Blackman**	**2.00**	**2.00**	**27.70**
**A7**	**34dB nHL**	**Left**	**Insert Earphones**	**0.80**	**Tone Burst**	**2000**	**Alternating**	**Blackman**	**2.00**	**2.00**	**27.70**
**A8**	**33dB nHL**	**Left**	**Insert Earphones**	**0.80**	**Tone Burst**	**2000**	**Alternating**	**Blackman**	**2.00**	**2.00**	**27.70**
**A9**	**32dB nHL**	**Left**	**Insert Earphones**	**0.80**	**Tone Burst**	**2000**	**Alternating**	**Blackman**	**2.00**	**2.00**	**27.70**
**A10**	**31dB nHL**	**Left**	**Insert Earphones**	**0.80**	**Tone Burst**	**2000**	**Alternating**	**Blackman**	**2.00**	**2.00**	**27.70**
**A11**	**31dB nHL**	**Left**	**Insert Earphones**	**0.80**	**Tone Burst**	**2000**	**Alternating**	**Blackman**	**2.00**	**2.00**	**27.70**
**A12**	**30dB nHL**	**Left**	**Insert Earphones**	**0.80**	**Tone Burst**	**2000**	**Alternating**	**Blackman**	**2.00**	**2.00**	**27.70**
**A13**	**29dB nHL**	**Left**	**Insert Earphones**	**0.80**	**Tone Burst**	**2000**	**Alternating**	**Blackman**	**2.00**	**2.00**	**27.70**
**A14**	**28dB nHL**	**Left**	**Insert Earphones**	**0.80**	**Tone Burst**	**2000**	**Alternating**	**Blackman**	**2.00**	**2.00**	**27.70**
**A15**	**27dB nHL**	**Left**	**Insert Earphones**	**0.80**	**Tone Burst**	**2000**	**Alternating**	**Blackman**	**2.00**	**2.00**	**27.70**
**A16**	**27dB nHL**	**Left**	**Insert Earphones**	**0.80**	**Tone Burst**	**2000**	**Alternating**	**Blackman**	**2.00**	**2.00**	**27.70**
**A17**	**26dB nHL**	**Left**	**Insert Earphones**	**0.80**	**Tone Burst**	**2000**	**Alternating**	**Blackman**	**2.00**	**2.00**	**27.70**
**A18**	**25dB nHL**	**Left**	**Insert Earphones**	**0.80**	**Tone Burst**	**2000**	**Alternating**	**Blackman**	**2.00**	**2.00**	**27.70**
**A19**	**24dB nHL**	**Left**	**Insert Earphones**	**0.80**	**Tone Burst**	**2000**	**Alternating**	**Blackman**	**2.00**	**2.00**	**27.70**
**A20**	**23dB nHL**	**Left**	**Insert Earphones**	**0.80**	**Tone Burst**	**2000**	**Alternating**	**Blackman**	**2.00**	**2.00**	**27.70**
**A21**	**23dB nHL**	**Left**	**Insert Earphones**	**0.80**	**Tone Burst**	**2000**	**Alternating**	**Blackman**	**2.00**	**2.00**	**27.70**
**A22**	**23dB nHL**	**Left**	**Insert Earphones**	**0.80**	**Tone Burst**	**2000**	**Alternating**	**Blackman**	**2.00**	**2.00**	**27.70**
**A23**	**22dB nHL**	**Left**	**Insert Earphones**	**0.80**	**Tone Burst**	**2000**	**Alternating**	**Blackman**	**2.00**	**2.00**	**27.70**
**A24**	**21dB nHL**	**Left**	**Insert Earphones**	**0.80**	**Tone Burst**	**2000**	**Alternating**	**Blackman**	**2.00**	**2.00**	**27.70**
**A25**	**19dB nHL**	**Left**	**Insert Earphones**	**0.80**	**Tone Burst**	**2000**	**Alternating**	**Blackman**	**2.00**	**2.00**	**27.70**
**A26**	**19dB nHL**	**Left**	**Insert Earphones**	**0.80**	**Tone Burst**	**2000**	**Alternating**	**Blackman**	**2.00**	**2.00**	**27.70**
**A27**	**19dB nHL**	**Left**	**Insert Earphones**	**0.80**	**Tone Burst**	**2000**	**Alternating**	**Blackman**	**2.00**	**2.00**	**27.70**
**A28**	**18dB nHL**	**Left**	**Insert Earphones**	**0.80**	**Tone Burst**	**2000**	**Alternating**	**Blackman**	**2.00**	**2.00**	**27.70**
**A29**	**18dB nHL**	**Left**	**Insert Earphones**	**0.80**	**Tone Burst**	**2000**	**Alternating**	**Blackman**	**2.00**	**2.00**	**27.70**
**A30**	**17dB nHL**	**Left**	**Insert Earphones**	**0.80**	**Tone Burst**	**2000**	**Alternating**	**Blackman**	**2.00**	**2.00**	**27.70**
**A31**	**17dB nHL**	**Left**	**Insert Earphones**	**0.80**	**Tone Burst**	**2000**	**Alternating**	**Blackman**	**2.00**	**2.00**	**27.70**
**A32**	**16dB nHL**	**Left**	**Insert Earphones**	**0.80**	**Tone Burst**	**2000**	**Alternating**	**Blackman**	**2.00**	**2.00**	**27.70**
**A33**	**15dB nHL**	**Left**	**Insert Earphones**	**0.80**	**Tone Burst**	**2000**	**Alternating**	**Blackman**	**2.00**	**2.00**	**27.70**

A34	14dB nHL	Left	Insert Earphones	0.80	Tone Burst	2000	Alternating	Blackman	2.00	2.00	27.70
A35	13dB nHL	Left	Insert Earphones	0.80	Tone Burst	2000	Alternating	Blackman	2.00	2.00	27.70
A36	12dB nHL	Left	Insert Earphones	0.80	Tone Burst	2000	Alternating	Blackman	2.00	2.00	27.70
A37	11dB nHL	Left	Insert Earphones	0.80	Tone Burst	2000	Alternating	Blackman	2.00	2.00	27.70
A38	10dB nHL	Left	Insert Earphones	0.80	Tone Burst	2000	Alternating	Blackman	2.00	2.00	27.70
A39	9dB nHL	Left	Insert Earphones	0.80	Tone Burst	2000	Alternating	Blackman	2.00	2.00	27.70
A40	9dB nHL	Left	Insert Earphones	0.80	Tone Burst	2000	Alternating	Blackman	2.00	2.00	27.70
A41	9dB nHL	Left	Insert Earphones	0.80	Tone Burst	2000	Alternating	Blackman	2.00	2.00	27.70
A42	8dB nHL	Left	Insert Earphones	0.80	Tone Burst	2000	Alternating	Blackman	2.00	2.00	27.70
A43	8dB nHL	Left	Insert Earphones	0.80	Tone Burst	2000	Alternating	Blackman	2.00	2.00	27.70
A44	7dB nHL	Left	Insert Earphones	0.80	Tone Burst	2000	Alternating	Blackman	2.00	2.00	27.70
A45	6dB nHL	Left	Insert Earphones	0.80	Tone Burst	2000	Alternating	Blackman	2.00	2.00	27.70
A46	6dB nHL	Left	Insert Earphones	0.80	Tone Burst	2000	Alternating	Blackman	2.00	2.00	27.70
A47	5dB nHL	Left	Insert Earphones	0.80	Tone Burst	2000	Alternating	Blackman	2.00	2.00	27.70
A48	4dB nHL	Left	Insert Earphones	0.80	Tone Burst	2000	Alternating	Blackman	2.00	2.00	27.70
A49	4dB nHL	Left	Insert Earphones	0.80	Tone Burst	2000	Alternating	Blackman	2.00	2.00	27.70
A50	3dB nHL	Left	Insert Earphones	0.80	Tone Burst	2000	Alternating	Blackman	2.00	2.00	27.70
A51	2dB nHL	Left	Insert Earphones	0.80	Tone Burst	2000	Alternating	Blackman	2.00	2.00	27.70
A52	2dB nHL	Left	Insert Earphones	0.80	Tone Burst	2000	Alternating	Blackman	2.00	2.00	27.70
A53	1dB nHL	Left	Insert Earphones	0.80	Tone Burst	2000	Alternating	Blackman	2.00	2.00	27.70

Recording Parameters

Label Index	*Epoch*	*Points*	*Pre/Post*	*Averages*	*Artifacts*
A1	16.00	256	0.00	1297	4
A2	16.00	256	0.00	910	9
A3	16.00	256	0.00	2374	8
A4	16.00	256	0.00	1966	8
A5	16.00	256	0.00	1562	5
A6	16.00	256	0.00	2359	11
A7	16.00	256	0.00	2420	14
A8	16.00	256	0.00	2184	12
A9	16.00	256	0.00	1674	11
A10	16.00	256	0.00	2217	10
A11	16.00	256	0.00	2102	8
A12	16.00	256	0.00	2086	10
A13	16.00	256	0.00	1357	7
A14	16.00	256	0.00	1217	8
A15	16.00	256	0.00	2052	13
A16	16.00	256	0.00	2370	13
A17	16.00	256	0.00	3349	8
A18	16.00	256	0.00	1143	9
A19	16.00	256	0.00	1739	9
A20	16.00	256	0.00	445	6

A21	16.00	256	0.00	1468	8
A22	16.00	256	0.00	1473	10
A23	16.00	256	0.00	1979	10
A24	16.00	256	0.00	1015	5
A25	16.00	256	0.00	1526	7
A26	16.00	256	0.00	712	7
A27	16.00	256	0.00	2066	6
A28	16.00	256	0.00	1679	8
A29	16.00	256	0.00	2151	9
A30	16.00	256	0.00	2469	5
A31	16.00	256	0.00	2007	9
A32	16.00	256	0.00	1425	7
A33	16.00	256	0.00	1546	8
A34	16.00	256	0.00	1136	8
A35	16.00	256	0.00	1626	9
A36	16.00	256	0.00	1993	9
A37	16.00	256	0.00	2612	10
A38	16.00	256	0.00	496	6
A39	16.00	256	0.00	1711	5
A40	16.00	256	0.00	2375	5
A41	16.00	256	0.00	2303	15
A42	16.00	256	0.00	2195	5
A43	16.00	256	0.00	1576	4
A44	16.00	256	0.00	1489	4
A45	16.00	256	0.00	1575	6
A46	16.00	256	0.00	1512	6
A47	16.00	256	0.00	1224	8
A48	16.00	256	0.00	1566	7
A49	16.00	256	0.00	4000	7
A50	16.00	256	0.00	1299	6
A51	16.00	256	0.00	1454	4
A52	16.00	256	0.00	1045	6
A53	16.00	256	0.00	779	3

Amplifier Parameters

Label Index	*Channel*	*Gain*	*Low Filter*	*High Filter*	*Notch Filter*	*Artifact Rejection*	*Input 1*	*Input 2*
A1	1	100000	30	1500	No	50.00	FZ	A1A2
A2	1	100000	30	1500	No	50.00	FZ	A1A2
A3	1	100000	30	1500	No	50.00	FZ	A1A2
A4	1	100000	30	1500	No	50.00	FZ	A1A2
A5	1	100000	30	1500	No	50.00	FZ	A1A2
A6	1	100000	30	1500	No	50.00	FZ	A1A2
A7	1	100000	30	1500	No	50.00	FZ	A1A2

**A8**	**1**	**100000**	**30**	**1500**	**No**	**50.00**	**FZ**	**A1A2**
**A9**	**1**	**100000**	**30**	**1500**	**No**	**50.00**	**FZ**	**A1A2**
**A10**	**1**	**100000**	**30**	**1500**	**No**	**50.00**	**FZ**	**A1A2**
**A11**	**1**	**100000**	**30**	**1500**	**No**	**50.00**	**FZ**	**A1A2**
**A12**	**1**	**100000**	**30**	**1500**	**No**	**50.00**	**FZ**	**A1A2**
**A13**	**1**	**100000**	**30**	**1500**	**No**	**50.00**	**FZ**	**A1A2**
**A14**	**1**	**100000**	**30**	**1500**	**No**	**50.00**	**FZ**	**A1A2**
**A15**	**1**	**100000**	**30**	**1500**	**No**	**50.00**	**FZ**	**A1A2**
**A16**	**1**	**100000**	**30**	**1500**	**No**	**50.00**	**FZ**	**A1A2**
**A17**	**1**	**100000**	**30**	**1500**	**No**	**50.00**	**FZ**	**A1A2**
**A18**	**1**	**100000**	**30**	**1500**	**No**	**50.00**	**FZ**	**A1A2**
**A19**	**1**	**100000**	**30**	**1500**	**No**	**50.00**	**FZ**	**A1A2**
**A20**	**1**	**100000**	**30**	**1500**	**No**	**50.00**	**FZ**	**A1A2**
**A21**	**1**	**100000**	**30**	**1500**	**No**	**50.00**	**FZ**	**A1A2**
**A22**	**1**	**100000**	**30**	**1500**	**No**	**50.00**	**FZ**	**A1A2**
**A23**	**1**	**100000**	**30**	**1500**	**No**	**50.00**	**FZ**	**A1A2**
**A24**	**1**	**100000**	**30**	**1500**	**No**	**50.00**	**FZ**	**A1A2**
**A25**	**1**	**100000**	**30**	**1500**	**No**	**50.00**	**FZ**	**A1A2**
**A26**	**1**	**100000**	**30**	**1500**	**No**	**50.00**	**FZ**	**A1A2**
**A27**	**1**	**100000**	**30**	**1500**	**No**	**50.00**	**FZ**	**A1A2**
**A28**	**1**	**100000**	**30**	**1500**	**No**	**50.00**	**FZ**	**A1A2**
**A29**	**1**	**100000**	**30**	**1500**	**No**	**50.00**	**FZ**	**A1A2**
**A30**	**1**	**100000**	**30**	**1500**	**No**	**50.00**	**FZ**	**A1A2**
**A31**	**1**	**100000**	**30**	**1500**	**No**	**50.00**	**FZ**	**A1A2**
**A32**	**1**	**100000**	**30**	**1500**	**No**	**50.00**	**FZ**	**A1A2**
**A33**	**1**	**100000**	**30**	**1500**	**No**	**50.00**	**FZ**	**A1A2**
**A34**	**1**	**100000**	**30**	**1500**	**No**	**50.00**	**FZ**	**A1A2**
**A35**	**1**	**100000**	**30**	**1500**	**No**	**50.00**	**FZ**	**A1A2**
**A36**	**1**	**100000**	**30**	**1500**	**No**	**50.00**	**FZ**	**A1A2**
**A37**	**1**	**100000**	**30**	**1500**	**No**	**50.00**	**FZ**	**A1A2**
**A38**	**1**	**100000**	**30**	**1500**	**No**	**50.00**	**FZ**	**A1A2**
**A39**	**1**	**100000**	**30**	**1500**	**No**	**50.00**	**FZ**	**A1A2**
**A40**	**1**	**100000**	**30**	**1500**	**No**	**50.00**	**FZ**	**A1A2**
**A41**	**1**	**100000**	**30**	**1500**	**No**	**50.00**	**FZ**	**A1A2**
**A42**	**1**	**100000**	**30**	**1500**	**No**	**50.00**	**FZ**	**A1A2**
**A43**	**1**	**100000**	**30**	**1500**	**No**	**50.00**	**FZ**	**A1A2**
**A44**	**1**	**100000**	**30**	**1500**	**No**	**50.00**	**FZ**	**A1A2**
**A45**	**1**	**100000**	**30**	**1500**	**No**	**50.00**	**FZ**	**A1A2**
**A46**	**1**	**100000**	**30**	**1500**	**No**	**50.00**	**FZ**	**A1A2**
**A47**	**1**	**100000**	**30**	**1500**	**No**	**50.00**	**FZ**	**A1A2**
**A48**	**1**	**100000**	**30**	**1500**	**No**	**50.00**	**FZ**	**A1A2**
**A49**	**1**	**100000**	**30**	**1500**	**No**	**50.00**	**FZ**	**A1A2**

[illegible]	1	100000	30	1500	No	50.00	FZ	A1A2
A51	1	100000	30	1500	No	50.00	FZ	A1A2
A52	1	100000	30	1500	No	50.00	FZ	A1A2
A53	1	100000	30	1500	No	50.00	FZ	A1A2
