# Supplementary material for: Auditory brainstem responses in the nine-banded armadillo (Dasypus novemcinctus)
Source: PeerJ. 2023 Dec 13;11:e16602. doi: 10.7717/peerj.16602 (PMC10725177; doi:10.7717/peerj.16602)
Supplement: Supplemental Information 2 — Each raw data file shows ABR amplitude (blue line) across various stimulus intensities (indicated on y-axis) over time in milliseconds (indicated on x-axis) for a particular experiment. [file peerj-11-16602-s002.zip › Armadillo 2021/#2 Animal F14-04 Case 15-06/500 Hz.pdf]

EVOKED POTENTIAL REPORT

UAMS CHP Speech and Hearing Clinic
Department of Audiology and Speech Pathology
4021 W. 8th Street
Little Rock, AR 72204
(501) 320-7300

Patient:	**case 1506 F14-04, Armadillo**
ID#:	**Armadillo 1506**
Gender:	
Birth date:	**02/10/15**

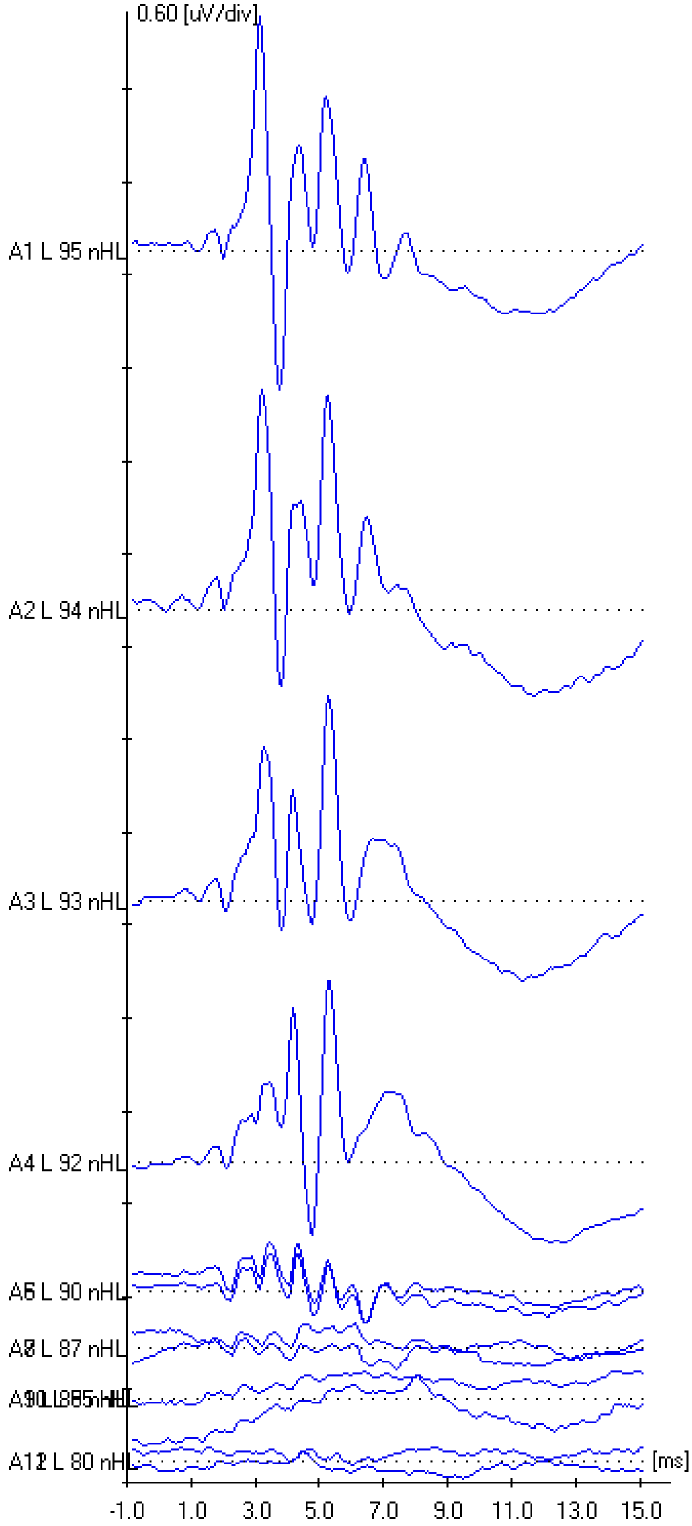

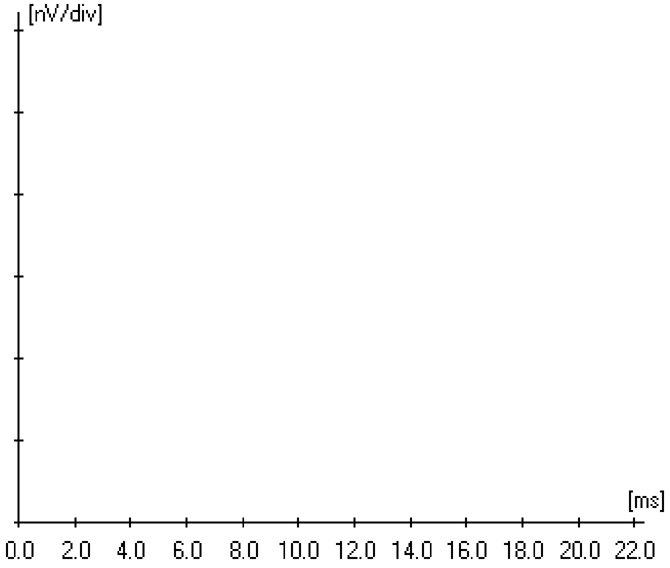

Latencies (ms)

Label Index	*I*	*II*	*III*	*IV*	*V*

Interlatencies (ms)

Label Index	*I-III*	*III-V*	*I-V*

Interaural Latency Differences

Label Index	*L1*	*L2*	*L3*	*L4*	*L5*	*L6*	*L7*	*L8*	*L9*	*L10*

Stimulus Parameters

Label Index	*Intensity*	*Ear*	*Transducer*	*Insert Delay*	*Type*	*Frequency*	*Polarity*	*Ramp*	*Rise/Fall*	*Plateau*	*Rate*

A1	95dB nHL	Left	Insert Earphones	0.80	Tone Burst	500	Alternating	Blackman	2.00	2.00	27.70
A2	94dB nHL	Left	Insert Earphones	0.80	Tone Burst	500	Alternating	Blackman	2.00	2.00	27.70
A3	93dB nHL	Left	Insert Earphones	0.80	Tone Burst	500	Alternating	Blackman	2.00	2.00	27.70
A4	92dB nHL	Left	Insert Earphones	0.80	Tone Burst	500	Alternating	Blackman	2.00	2.00	27.70
A5	90dB nHL	Left	Insert Earphones	0.80	Tone Burst	500	Alternating	Blackman	2.00	2.00	27.70
A6	90dB nHL	Left	Insert Earphones	0.80	Tone Burst	500	Alternating	Blackman	2.00	2.00	27.70
A7	87dB nHL	Left	Insert Earphones	0.80	Tone Burst	500	Alternating	Blackman	2.00	2.00	27.70
A8	87dB nHL	Left	Insert Earphones	0.80	Tone Burst	500	Alternating	Blackman	2.00	2.00	27.70
A9	85dB nHL	Left	Insert Earphones	0.80	Tone Burst	500	Alternating	Blackman	2.00	2.00	27.70
A10	85dB nHL	Left	Insert Earphones	0.80	Tone Burst	500	Alternating	Blackman	2.00	2.00	27.70
A11	80dB nHL	Left	Insert Earphones	0.80	Tone Burst	500	Alternating	Blackman	2.00	2.00	27.70
A12	80dB nHL	Left	Insert Earphones	0.80	Tone Burst	500	Alternating	Blackman	2.00	2.00	27.70

Recording Parameters

Label Index	*Epoch*	*Points*	*Pre/Post*	*Averages*	*Artifacts*
A1	16.00	256	0.00	1073	7
A2	16.00	256	0.00	626	4
A3	16.00	256	0.00	1391	7
A4	16.00	256	0.00	2307	12
A5	16.00	256	0.00	2021	4
A6	16.00	256	0.00	2550	9
A7	16.00	256	0.00	1648	8
A8	16.00	256	0.00	1528	7
A9	16.00	256	0.00	926	4
A10	16.00	256	0.00	1263	3
A11	16.00	256	0.00	1759	5
A12	16.00	256	0.00	1953	6

Amplifier Parameters

Label Index	*Channel*	*Gain*	*Low Filter*	*High Filter*	*Notch Filter*	*Artifact Rejection*	*Input 1*	*Input 2*
A1	1	100000	30	1500	No	50.00	FZ	A1A2
A2	1	100000	30	1500	No	50.00	FZ	A1A2
A3	1	100000	30	1500	No	50.00	FZ	A1A2
A4	1	100000	30	1500	No	50.00	FZ	A1A2
A5	1	100000	30	1500	No	50.00	FZ	A1A2
A6	1	100000	30	1500	No	50.00	FZ	A1A2
A7	1	100000	30	1500	No	50.00	FZ	A1A2
A8	1	100000	30	1500	No	50.00	FZ	A1A2
A9	1	100000	30	1500	No	50.00	FZ	A1A2
A10	1	100000	30	1500	No	50.00	FZ	A1A2
A11	1	100000	30	1500	No	50.00	FZ	A1A2
A12	1	100000	30	1500	No	50.00	FZ	A1A2
