# Supplementary material for: Auditory brainstem responses in the nine-banded armadillo (Dasypus novemcinctus)
Source: PeerJ. 2023 Dec 13;11:e16602. doi: 10.7717/peerj.16602 (PMC10725177; doi:10.7717/peerj.16602)
Supplement: Supplemental Information 2 — Each raw data file shows ABR amplitude (blue line) across various stimulus intensities (indicated on y-axis) over time in milliseconds (indicated on x-axis) for a particular experiment. [file peerj-11-16602-s002.zip › Armadillo 2021/#2 Animal F14-04 Case 15-06/All other frequencies by record number.pdf]

EVOKED POTENTIAL REPORT

UAMS CHP Speech and Hearing Clinic
Department of Audiology and Speech Pathology
4021 W. 8th Street
Little Rock, AR 72204
(501) 320-7300

Patient:	**case 1506 F14-04, Armadillo**
ID#:	**Armadillo 1506**
Gender:	
Birth date:	**02/10/15**

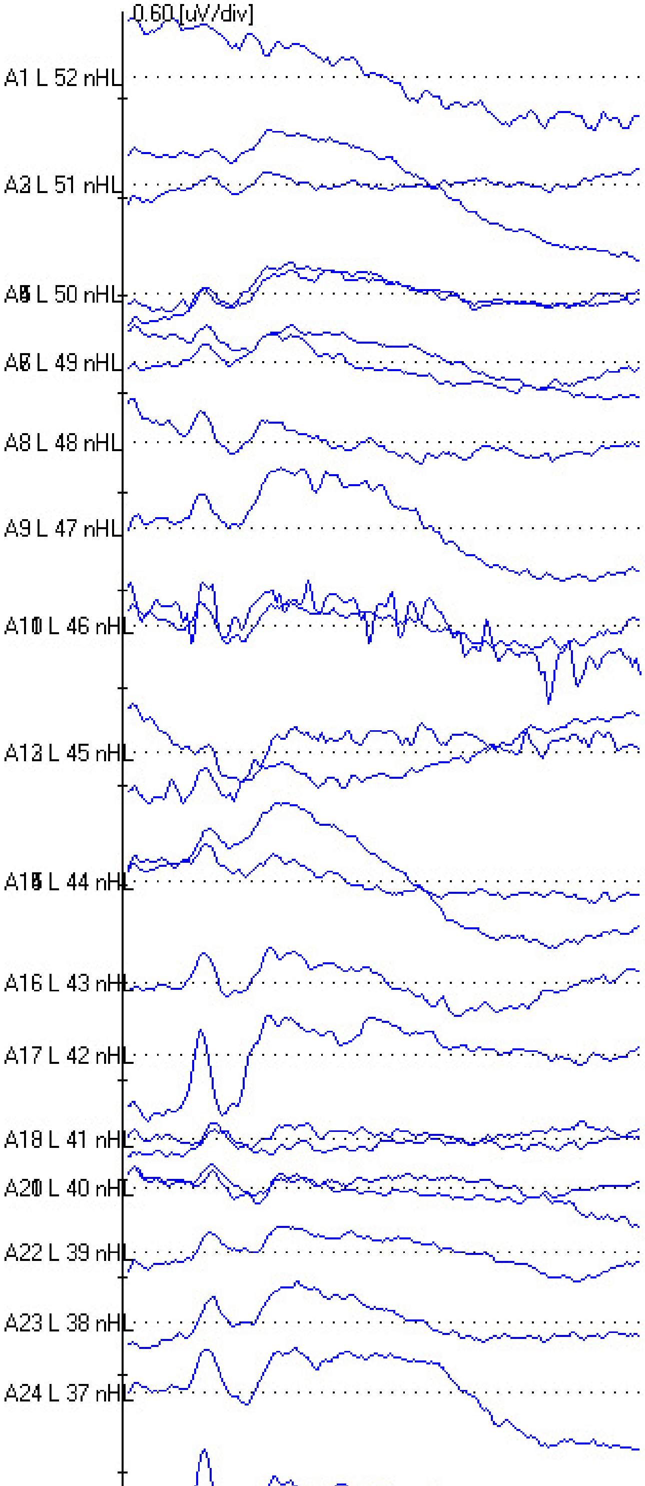

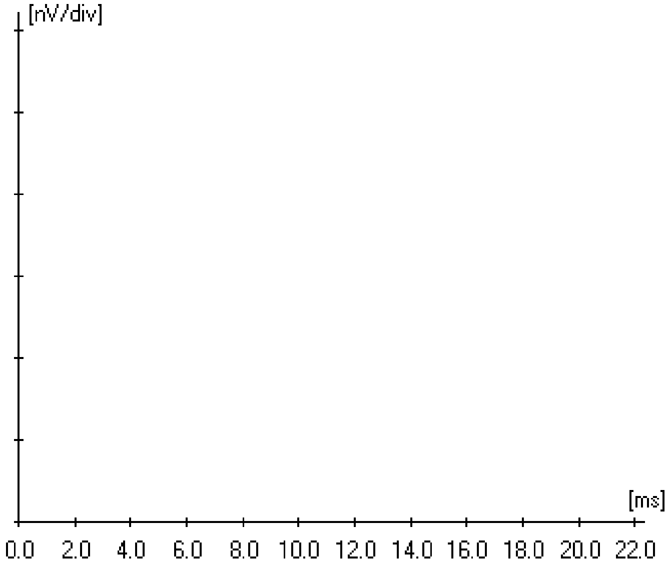

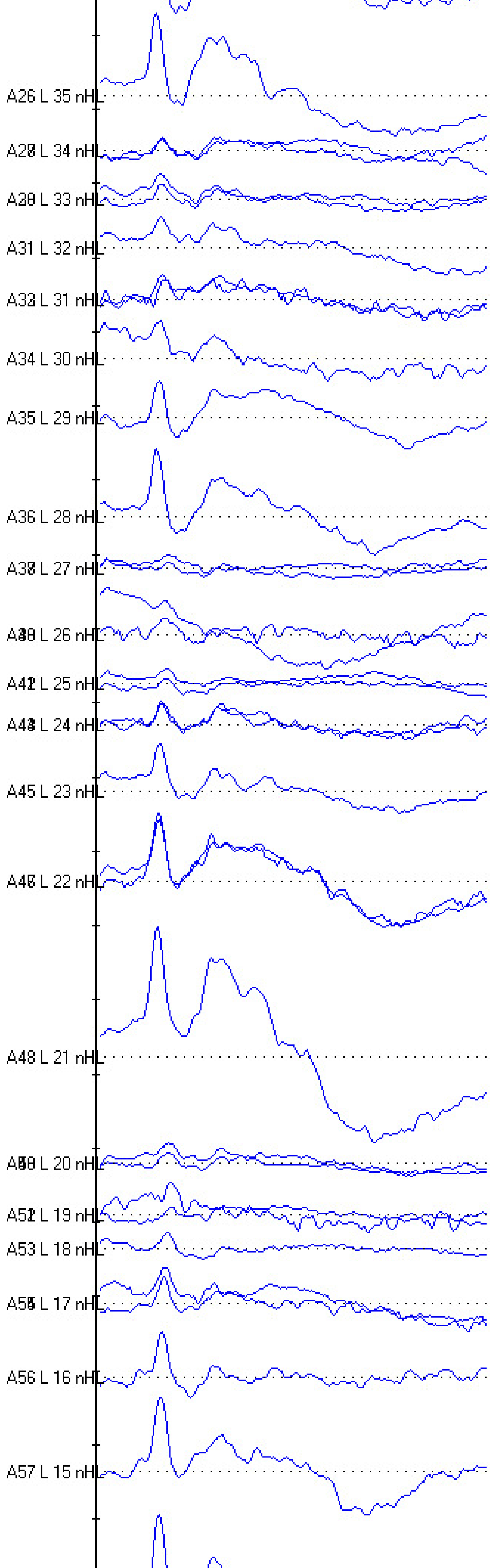
A26 L 35 nHL
L 34 nHL
L 33 nHL
A31 L 32 nHL
L 31 nHL
A34 L 30 nHL
A35 L 29 nHL
A36 L 28 nHL
L 27 nHL
L 26 nHL
L 25 nHL
L 24 nHL
A45 L 23 nHL
L 22 nHL
A48 L 21 nHL
L 20 nHL
L 19 nHL
A53 L 18 nHL
L 17 nHL
A56 L 16 nHL
A57 L 15 nHL

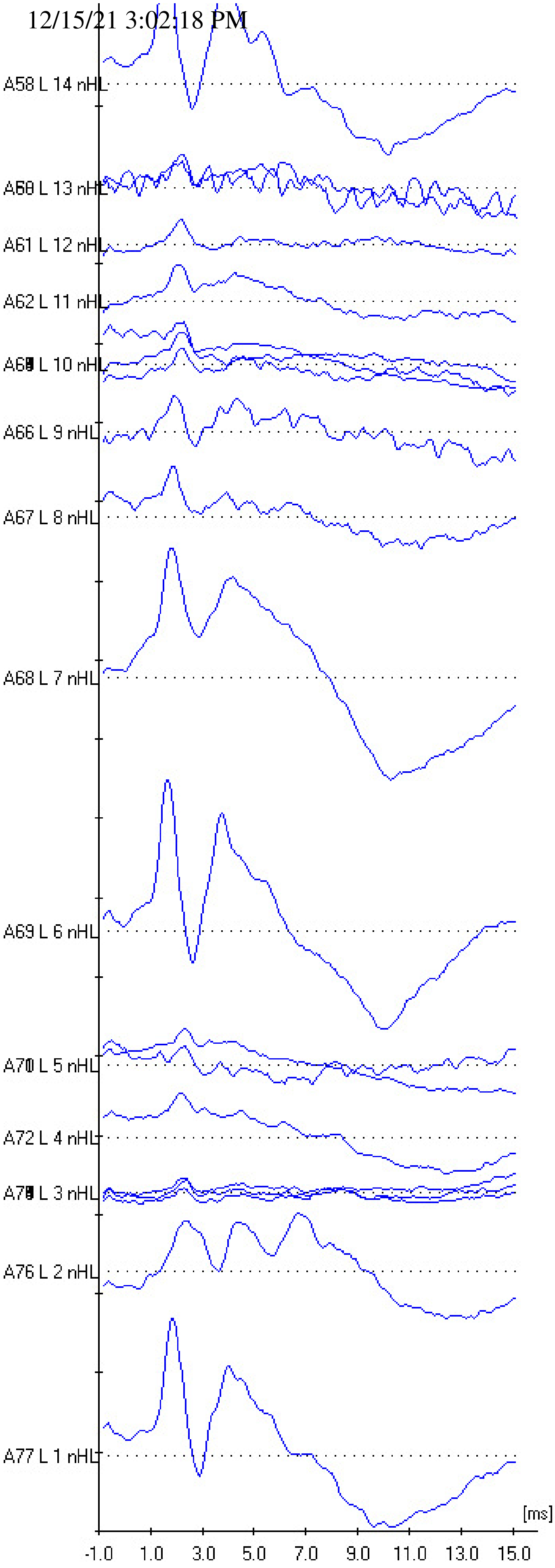
A58 L 14 nHL
L 13 nHL
A61 L 12 nHL
A62 L 11 nHL
L 10 nHL
A66 L 9 nHL
A67 L 8 nHL
A68 L 7 nHL
A69 L 6 nHL
L 5 nHL
A72 L 4 nHL
L 3 nHL
A76 L 2 nHL
A77 L 1 nHL
[ms]
-1.0
1.0
3.0
5.0
7.0
9.0
11.0
13.0
15.0

Latencies (ms)

Label Index	*I*	*II*	*III*	*IV*	*V*

Interlatencies (ms)

Label Index	*I-III*	*III-V*	*I-V*

Interaural Latency Differences

Label Index	*L1*	*L2*	*L3*	*L4*	*L5*	*L6*	*L7*	*L8*	*L9*	*L10*

Stimulus Parameters

Label Index	*Intensity*	*Ear*	*Transducer*	*Insert Delay*	*Type*	*Frequency*	*Polarity*	*Ramp*	*Rise/Fall*	*Plateau*	*Rate*
**A1**	**52dB nHL**	**Left**	**Insert Earphones**	**0.80**	**Tone Burst**	**2000**	**Alternating**	**Blackman**	**2.00**	**2.00**	**27.70**
**A2**	**51dB nHL**	**Left**	**Insert Earphones**	**0.80**	**Tone Burst**	**2000**	**Alternating**	**Blackman**	**2.00**	**2.00**	**27.70**
**A3**	**51dB nHL**	**Left**	**Insert Earphones**	**0.80**	**Tone Burst**	**2000**	**Alternating**	**Blackman**	**2.00**	**2.00**	**27.70**
**A4**	**50dB nHL**	**Left**	**Insert Earphones**	**0.80**	**Tone Burst**	**2000**	**Alternating**	**Blackman**	**2.00**	**2.00**	**27.70**
**A5**	**50dB nHL**	**Left**	**Insert Earphones**	**0.80**	**Tone Burst**	**2000**	**Alternating**	**Blackman**	**2.00**	**2.00**	**27.70**
**A6**	**49dB nHL**	**Left**	**Insert Earphones**	**0.80**	**Tone Burst**	**2000**	**Alternating**	**Blackman**	**2.00**	**2.00**	**27.70**
**A7**	**49dB nHL**	**Left**	**Insert Earphones**	**0.80**	**Tone Burst**	**2000**	**Alternating**	**Blackman**	**2.00**	**2.00**	**27.70**
**A8**	**48dB nHL**	**Left**	**Insert Earphones**	**0.80**	**Tone Burst**	**2000**	**Alternating**	**Blackman**	**2.00**	**2.00**	**27.70**
**A9**	**47dB nHL**	**Left**	**Insert Earphones**	**0.80**	**Tone Burst**	**2000**	**Alternating**	**Blackman**	**2.00**	**2.00**	**27.70**
**A10**	**46dB nHL**	**Left**	**Insert Earphones**	**0.80**	**Tone Burst**	**2000**	**Alternating**	**Blackman**	**2.00**	**2.00**	**27.70**
**A11**	**46dB nHL**	**Left**	**Insert Earphones**	**0.80**	**Tone Burst**	**2000**	**Alternating**	**Blackman**	**2.00**	**2.00**	**27.70**
**A12**	**45dB nHL**	**Left**	**Insert Earphones**	**0.80**	**Tone Burst**	**2000**	**Alternating**	**Blackman**	**2.00**	**2.00**	**27.70**
**A13**	**45dB nHL**	**Left**	**Insert Earphones**	**0.80**	**Tone Burst**	**2000**	**Alternating**	**Blackman**	**2.00**	**2.00**	**27.70**
**A14**	**44dB nHL**	**Left**	**Insert Earphones**	**0.80**	**Tone Burst**	**2000**	**Alternating**	**Blackman**	**2.00**	**2.00**	**27.70**
**A15**	**44dB nHL**	**Left**	**Insert Earphones**	**0.80**	**Tone Burst**	**2000**	**Alternating**	**Blackman**	**2.00**	**2.00**	**27.70**
**A16**	**43dB nHL**	**Left**	**Insert Earphones**	**0.80**	**Tone Burst**	**2000**	**Alternating**	**Blackman**	**2.00**	**2.00**	**27.70**
**A17**	**42dB nHL**	**Left**	**Insert Earphones**	**0.80**	**Tone Burst**	**2000**	**Alternating**	**Blackman**	**2.00**	**2.00**	**27.70**
**A18**	**41dB nHL**	**Left**	**Insert Earphones**	**0.80**	**Tone Burst**	**2000**	**Alternating**	**Blackman**	**2.00**	**2.00**	**27.70**
**A19**	**41dB nHL**	**Left**	**Insert Earphones**	**0.80**	**Tone Burst**	**2000**	**Alternating**	**Blackman**	**2.00**	**2.00**	**27.70**
**A20**	**40dB nHL**	**Left**	**Insert Earphones**	**0.80**	**Tone Burst**	**2000**	**Alternating**	**Blackman**	**2.00**	**2.00**	**27.70**
**A21**	**40dB nHL**	**Left**	**Insert Earphones**	**0.80**	**Tone Burst**	**2000**	**Alternating**	**Blackman**	**2.00**	**2.00**	**27.70**
**A22**	**39dB nHL**	**Left**	**Insert Earphones**	**0.80**	**Tone Burst**	**2000**	**Alternating**	**Blackman**	**2.00**	**2.00**	**27.70**
**A23**	**38dB nHL**	**Left**	**Insert Earphones**	**0.80**	**Tone Burst**	**2000**	**Alternating**	**Blackman**	**2.00**	**2.00**	**27.70**
**A24**	**37dB nHL**	**Left**	**Insert Earphones**	**0.80**	**Tone Burst**	**2000**	**Alternating**	**Blackman**	**2.00**	**2.00**	**27.70**
**A25**	**36dB nHL**	**Left**	**Insert Earphones**	**0.80**	**Tone Burst**	**2000**	**Alternating**	**Blackman**	**2.00**	**2.00**	**27.70**
**A26**	**35dB nHL**	**Left**	**Insert Earphones**	**0.80**	**Tone Burst**	**2000**	**Alternating**	**Blackman**	**2.00**	**2.00**	**27.70**
**A27**	**34dB nHL**	**Left**	**Insert Earphones**	**0.80**	**Tone Burst**	**2000**	**Alternating**	**Blackman**	**2.00**	**2.00**	**27.70**
**A28**	**34dB nHL**	**Left**	**Insert Earphones**	**0.80**	**Tone Burst**	**2000**	**Alternating**	**Blackman**	**2.00**	**2.00**	**27.70**
**A29**	**33dB nHL**	**Left**	**Insert Earphones**	**0.80**	**Tone Burst**	**2000**	**Alternating**	**Blackman**	**2.00**	**2.00**	**27.70**
**A30**	**33dB nHL**	**Left**	**Insert Earphones**	**0.80**	**Tone Burst**	**2000**	**Alternating**	**Blackman**	**2.00**	**2.00**	**27.70**

A31	32dB nHL	Left	Insert Earphones	0.80	Tone Burst	2000	Alternating	Blackman	2.00	2.00	27.70
A32	31dB nHL	Left	Insert Earphones	0.80	Tone Burst	2000	Alternating	Blackman	2.00	2.00	27.70
A33	31dB nHL	Left	Insert Earphones	0.80	Tone Burst	2000	Alternating	Blackman	2.00	2.00	27.70
A34	30dB nHL	Left	Insert Earphones	0.80	Tone Burst	2000	Alternating	Blackman	2.00	2.00	27.70
A35	29dB nHL	Left	Insert Earphones	0.80	Tone Burst	2000	Alternating	Blackman	2.00	2.00	27.70
A36	28dB nHL	Left	Insert Earphones	0.80	Tone Burst	2000	Alternating	Blackman	2.00	2.00	27.70
A37	27dB nHL	Left	Insert Earphones	0.80	Tone Burst	2000	Alternating	Blackman	2.00	2.00	27.70
A38	27dB nHL	Left	Insert Earphones	0.80	Tone Burst	2000	Alternating	Blackman	2.00	2.00	27.70
A39	26dB nHL	Left	Insert Earphones	0.80	Tone Burst	2000	Alternating	Blackman	2.00	2.00	27.70
A40	26dB nHL	Left	Insert Earphones	0.80	Tone Burst	2000	Alternating	Blackman	2.00	2.00	27.70
A41	25dB nHL	Left	Insert Earphones	0.80	Tone Burst	2000	Alternating	Blackman	2.00	2.00	27.70
A42	25dB nHL	Left	Insert Earphones	0.80	Tone Burst	2000	Alternating	Blackman	2.00	2.00	27.70
A43	24dB nHL	Left	Insert Earphones	0.80	Tone Burst	2000	Alternating	Blackman	2.00	2.00	27.70
A44	24dB nHL	Left	Insert Earphones	0.80	Tone Burst	2000	Alternating	Blackman	2.00	2.00	27.70
A45	23dB nHL	Left	Insert Earphones	0.80	Tone Burst	2000	Alternating	Blackman	2.00	2.00	27.70
A46	22dB nHL	Left	Insert Earphones	0.80	Tone Burst	2000	Alternating	Blackman	2.00	2.00	27.70
A47	22dB nHL	Left	Insert Earphones	0.80	Tone Burst	2000	Alternating	Blackman	2.00	2.00	27.70
A48	21dB nHL	Left	Insert Earphones	0.80	Tone Burst	2000	Alternating	Blackman	2.00	2.00	27.70
A49	20dB nHL	Left	Insert Earphones	0.80	Tone Burst	2000	Alternating	Blackman	2.00	2.00	27.70
A50	20dB nHL	Left	Insert Earphones	0.80	Tone Burst	2000	Alternating	Blackman	2.00	2.00	27.70
A51	19dB nHL	Left	Insert Earphones	0.80	Tone Burst	2000	Alternating	Blackman	2.00	2.00	27.70
A52	19dB nHL	Left	Insert Earphones	0.80	Tone Burst	2000	Alternating	Blackman	2.00	2.00	27.70
A53	18dB nHL	Left	Insert Earphones	0.80	Tone Burst	2000	Alternating	Blackman	2.00	2.00	27.70
A54	17dB nHL	Left	Insert Earphones	0.80	Tone Burst	2000	Alternating	Blackman	2.00	2.00	27.70
A55	17dB nHL	Left	Insert Earphones	0.80	Tone Burst	2000	Alternating	Blackman	2.00	2.00	27.70
A56	16dB nHL	Left	Insert Earphones	0.80	Tone Burst	2000	Alternating	Blackman	2.00	2.00	27.70
A57	15dB nHL	Left	Insert Earphones	0.80	Tone Burst	2000	Alternating	Blackman	2.00	2.00	27.70
A58	14dB nHL	Left	Insert Earphones	0.80	Tone Burst	2000	Alternating	Blackman	2.00	2.00	27.70
A59	13dB nHL	Left	Insert Earphones	0.80	Tone Burst	2000	Alternating	Blackman	2.00	2.00	27.70
A60	13dB nHL	Left	Insert Earphones	0.80	Tone Burst	2000	Alternating	Blackman	2.00	2.00	27.70
A61	12dB nHL	Left	Insert Earphones	0.80	Tone Burst	2000	Alternating	Blackman	2.00	2.00	27.70
A62	11dB nHL	Left	Insert Earphones	0.80	Tone Burst	2000	Alternating	Blackman	2.00	2.00	27.70
A63	10dB nHL	Left	Insert Earphones	0.80	Tone Burst	2000	Alternating	Blackman	2.00	2.00	27.70
A64	10dB nHL	Left	Insert Earphones	0.80	Tone Burst	2000	Alternating	Blackman	2.00	2.00	27.70
A65	10dB nHL	Left	Insert Earphones	0.80	Tone Burst	2000	Alternating	Blackman	2.00	2.00	27.70
A66	9dB nHL	Left	Insert Earphones	0.80	Tone Burst	2000	Alternating	Blackman	2.00	2.00	27.70
A67	8dB nHL	Left	Insert Earphones	0.80	Tone Burst	2000	Alternating	Blackman	2.00	2.00	27.70
A68	7dB nHL	Left	Insert Earphones	0.80	Tone Burst	2000	Alternating	Blackman	2.00	2.00	27.70
A69	6dB nHL	Left	Insert Earphones	0.80	Tone Burst	2000	Alternating	Blackman	2.00	2.00	27.70
A70	5dB nHL	Left	Insert Earphones	0.80	Tone Burst	2000	Alternating	Blackman	2.00	2.00	27.70
A71	5dB nHL	Left	Insert Earphones	0.80	Tone Burst	2000	Alternating	Blackman	2.00	2.00	27.70
A72	4dB nHL	Left	Insert Earphones	0.80	Tone Burst	2000	Alternating	Blackman	2.00	2.00	27.70

A7[illegible]	[illegible]dB nHL	Left	Insert Earphones	0.80	Tone Burst	2000	Alternating	Blackman	2.00	2.00	27.70
A74	3dB nHL	Left	Insert Earphones	0.80	Tone Burst	2000	Alternating	Blackman	2.00	2.00	27.70
A75	3dB nHL	Left	Insert Earphones	0.80	Tone Burst	2000	Alternating	Blackman	2.00	2.00	27.70
A76	2dB nHL	Left	Insert Earphones	0.80	Tone Burst	2000	Alternating	Blackman	2.00	2.00	27.70
A77	1dB nHL	Left	Insert Earphones	0.80	Tone Burst	2000	Alternating	Blackman	2.00	2.00	27.70

Recording Parameters

Label Index	*Epoch*	*Points*	*Pre/Post*	*Averages*	*Artifacts*
A1	16.00	256	0.00	1182	5
A2	16.00	256	0.00	1501	5
A3	16.00	256	0.00	1297	7
A4	16.00	256	0.00	1537	4
A5	16.00	256	0.00	1163	9
A6	16.00	256	0.00	920	8
A7	16.00	256	0.00	2269	9
A8	16.00	256	0.00	691	4
A9	16.00	256	0.00	523	3
A10	16.00	256	0.00	1034	7
A11	16.00	256	0.00	612	4
A12	16.00	256	0.00	705	6
A13	16.00	256	0.00	732	6
A14	16.00	256	0.00	1014	7
A15	16.00	256	0.00	750	5
A16	16.00	256	0.00	623	5
A17	16.00	256	0.00	627	7
A18	16.00	256	0.00	1008	5
A19	16.00	256	0.00	1023	7
A20	16.00	256	0.00	1002	3
A21	16.00	256	0.00	2031	7
A22	16.00	256	0.00	1277	8
A23	16.00	256	0.00	1016	8
A24	16.00	256	0.00	663	5
A25	16.00	256	0.00	550	5
A26	16.00	256	0.00	696	2
A27	16.00	256	0.00	1044	6
A28	16.00	256	0.00	1392	5
A29	16.00	256	0.00	1304	5
A30	16.00	256	0.00	2016	5
A31	16.00	256	0.00	886	4
A32	16.00	256	0.00	1520	8
A33	16.00	256	0.00	1396	5
A34	16.00	256	0.00	1054	9
A35	16.00	256	0.00	1104	8

A36	16.00	256	0.00	692	7
A37	16.00	256	0.00	1460	7
A38	16.00	256	0.00	3314	8
A39	16.00	256	0.00	2280	6
A40	16.00	256	0.00	961	5
A41	16.00	256	0.00	1752	9
A42	16.00	256	0.00	2958	8
A43	16.00	256	0.00	2075	6
A44	16.00	256	0.00	2112	7
A45	16.00	256	0.00	1044	7
A46	16.00	256	0.00	1600	9
A47	16.00	256	0.00	1294	7
A48	16.00	256	0.00	1508	3
A49	16.00	256	0.00	1515	6
A50	16.00	256	0.00	1860	10
A51	16.00	256	0.00	1849	6
A52	16.00	256	0.00	1977	9
A53	16.00	256	0.00	1819	7
A54	16.00	256	0.00	1325	9
A55	16.00	256	0.00	1556	6
A56	16.00	256	0.00	1276	9
A57	16.00	256	0.00	601	6
A58	16.00	256	0.00	856	4
A59	16.00	256	0.00	1980	6
A60	16.00	256	0.00	829	4
A61	16.00	256	0.00	1154	7
A62	16.00	256	0.00	1345	9
A63	16.00	256	0.00	799	5
A64	16.00	256	0.00	2624	9
A65	16.00	256	0.00	1630	7
A66	16.00	256	0.00	1950	8
A67	16.00	256	0.00	2438	10
A68	16.00	256	0.00	1111	4
A69	16.00	256	0.00	759	5
A70	16.00	256	0.00	2262	12
A71	16.00	256	0.00	1873	7
A72	16.00	256	0.00	1696	9
A73	16.00	256	0.00	3007	9
A74	16.00	256	0.00	3928	10
A75	16.00	256	0.00	3783	9
A76	16.00	256	0.00	915	3
A77	16.00	256	0.00	1518	6

Amplifier Parameters

Label Index	*Channel*	*Gain*	*Low Filter*	*High Filter*	*Notch Filter*	*Artifact Rejection*	*Input 1*	*Input 2*
**A1**	**1**	**100000**	**30**	**1500**	**No**	**50.00**	**FZ**	**A1A2**
**A2**	**1**	**100000**	**30**	**1500**	**No**	**50.00**	**FZ**	**A1A2**
**A3**	**1**	**100000**	**30**	**1500**	**No**	**50.00**	**FZ**	**A1A2**
**A4**	**1**	**100000**	**30**	**1500**	**No**	**50.00**	**FZ**	**A1A2**
**A5**	**1**	**100000**	**30**	**1500**	**No**	**50.00**	**FZ**	**A1A2**
**A6**	**1**	**100000**	**30**	**1500**	**No**	**50.00**	**FZ**	**A1A2**
**A7**	**1**	**100000**	**30**	**1500**	**No**	**50.00**	**FZ**	**A1A2**
**A8**	**1**	**100000**	**30**	**1500**	**No**	**50.00**	**FZ**	**A1A2**
**A9**	**1**	**100000**	**30**	**1500**	**No**	**50.00**	**FZ**	**A1A2**
**A10**	**1**	**100000**	**30**	**1500**	**No**	**50.00**	**FZ**	**A1A2**
**A11**	**1**	**100000**	**30**	**1500**	**No**	**50.00**	**FZ**	**A1A2**
**A12**	**1**	**100000**	**30**	**1500**	**No**	**50.00**	**FZ**	**A1A2**
**A13**	**1**	**100000**	**30**	**1500**	**No**	**50.00**	**FZ**	**A1A2**
**A14**	**1**	**100000**	**30**	**1500**	**No**	**50.00**	**FZ**	**A1A2**
**A15**	**1**	**100000**	**30**	**1500**	**No**	**50.00**	**FZ**	**A1A2**
**A16**	**1**	**100000**	**30**	**1500**	**No**	**50.00**	**FZ**	**A1A2**
**A17**	**1**	**100000**	**30**	**1500**	**No**	**50.00**	**FZ**	**A1A2**
**A18**	**1**	**100000**	**30**	**1500**	**No**	**50.00**	**FZ**	**A1A2**
**A19**	**1**	**100000**	**30**	**1500**	**No**	**50.00**	**FZ**	**A1A2**
**A20**	**1**	**100000**	**30**	**1500**	**No**	**50.00**	**FZ**	**A1A2**
**A21**	**1**	**100000**	**30**	**1500**	**No**	**50.00**	**FZ**	**A1A2**
**A22**	**1**	**100000**	**30**	**1500**	**No**	**50.00**	**FZ**	**A1A2**
**A23**	**1**	**100000**	**30**	**1500**	**No**	**50.00**	**FZ**	**A1A2**
**A24**	**1**	**100000**	**30**	**1500**	**No**	**50.00**	**FZ**	**A1A2**
**A25**	**1**	**100000**	**30**	**1500**	**No**	**50.00**	**FZ**	**A1A2**
**A26**	**1**	**100000**	**30**	**1500**	**No**	**50.00**	**FZ**	**A1A2**
**A27**	**1**	**100000**	**30**	**1500**	**No**	**50.00**	**FZ**	**A1A2**
**A28**	**1**	**100000**	**30**	**1500**	**No**	**50.00**	**FZ**	**A1A2**
**A29**	**1**	**100000**	**30**	**1500**	**No**	**50.00**	**FZ**	**A1A2**
**A30**	**1**	**100000**	**30**	**1500**	**No**	**50.00**	**FZ**	**A1A2**
**A31**	**1**	**100000**	**30**	**1500**	**No**	**50.00**	**FZ**	**A1A2**
**A32**	**1**	**100000**	**30**	**1500**	**No**	**50.00**	**FZ**	**A1A2**
**A33**	**1**	**100000**	**30**	**1500**	**No**	**50.00**	**FZ**	**A1A2**
**A34**	**1**	**100000**	**30**	**1500**	**No**	**50.00**	**FZ**	**A1A2**
**A35**	**1**	**100000**	**30**	**1500**	**No**	**50.00**	**FZ**	**A1A2**
**A36**	**1**	**100000**	**30**	**1500**	**No**	**50.00**	**FZ**	**A1A2**
**A37**	**1**	**100000**	**30**	**1500**	**No**	**50.00**	**FZ**	**A1A2**
**A38**	**1**	**100000**	**30**	**1500**	**No**	**50.00**	**FZ**	**A1A2**
**A39**	**1**	**100000**	**30**	**1500**	**No**	**50.00**	**FZ**	**A1A2**
**A40**	**1**	**100000**	**30**	**1500**	**No**	**50.00**	**FZ**	**A1A2**

A41	1	100000	30	1500	No	50.00	FZ	A1A2
A42	1	100000	30	1500	No	50.00	FZ	A1A2
A43	1	100000	30	1500	No	50.00	FZ	A1A2
A44	1	100000	30	1500	No	50.00	FZ	A1A2
A45	1	100000	30	1500	No	50.00	FZ	A1A2
A46	1	100000	30	1500	No	50.00	FZ	A1A2
A47	1	100000	30	1500	No	50.00	FZ	A1A2
A48	1	100000	30	1500	No	50.00	FZ	A1A2
A49	1	100000	30	1500	No	50.00	FZ	A1A2
A50	1	100000	30	1500	No	50.00	FZ	A1A2
A51	1	100000	30	1500	No	50.00	FZ	A1A2
A52	1	100000	30	1500	No	50.00	FZ	A1A2
A53	1	100000	30	1500	No	50.00	FZ	A1A2
A54	1	100000	30	1500	No	50.00	FZ	A1A2
A55	1	100000	30	1500	No	50.00	FZ	A1A2
A56	1	100000	30	1500	No	50.00	FZ	A1A2
A57	1	100000	30	1500	No	50.00	FZ	A1A2
A58	1	100000	30	1500	No	50.00	FZ	A1A2
A59	1	100000	30	1500	No	50.00	FZ	A1A2
A60	1	100000	30	1500	No	50.00	FZ	A1A2
A61	1	100000	30	1500	No	50.00	FZ	A1A2
A62	1	100000	30	1500	No	50.00	FZ	A1A2
A63	1	100000	30	1500	No	50.00	FZ	A1A2
A64	1	100000	30	1500	No	50.00	FZ	A1A2
A65	1	100000	30	1500	No	50.00	FZ	A1A2
A66	1	100000	30	1500	No	50.00	FZ	A1A2
A67	1	100000	30	1500	No	50.00	FZ	A1A2
A68	1	100000	30	1500	No	50.00	FZ	A1A2
A69	1	100000	30	1500	No	50.00	FZ	A1A2
A70	1	100000	30	1500	No	50.00	FZ	A1A2
A71	1	100000	30	1500	No	50.00	FZ	A1A2
A72	1	100000	30	1500	No	50.00	FZ	A1A2
A73	1	100000	30	1500	No	50.00	FZ	A1A2
A74	1	100000	30	1500	No	50.00	FZ	A1A2
A75	1	100000	30	1500	No	50.00	FZ	A1A2
A76	1	100000	30	1500	No	50.00	FZ	A1A2
A77	1	100000	30	1500	No	50.00	FZ	A1A2
