# Supplementary material for: Auditory brainstem responses in the nine-banded armadillo (Dasypus novemcinctus)
Source: PeerJ. 2023 Dec 13;11:e16602. doi: 10.7717/peerj.16602 (PMC10725177; doi:10.7717/peerj.16602)
Supplement: Supplemental Information 2 — Each raw data file shows ABR amplitude (blue line) across various stimulus intensities (indicated on y-axis) over time in milliseconds (indicated on x-axis) for a particular experiment. [file peerj-11-16602-s002.zip › Armadillo 2021/Animal 15-01 Case 15-07/2000 Hz.pdf]

EVOKED POTENTIAL REPORT

UAMS CHP Speech and Hearing Clinic
Department of Audiology and Speech Pathology
4021 W. 8th Street
Little Rock, AR 72204
(501) 320-7300

Patient:	**Case 1507 animal 15-01, Armadillo**
ID#:	**Armadillo 1507**
Gender:	
Birth date:	**02/11/15**

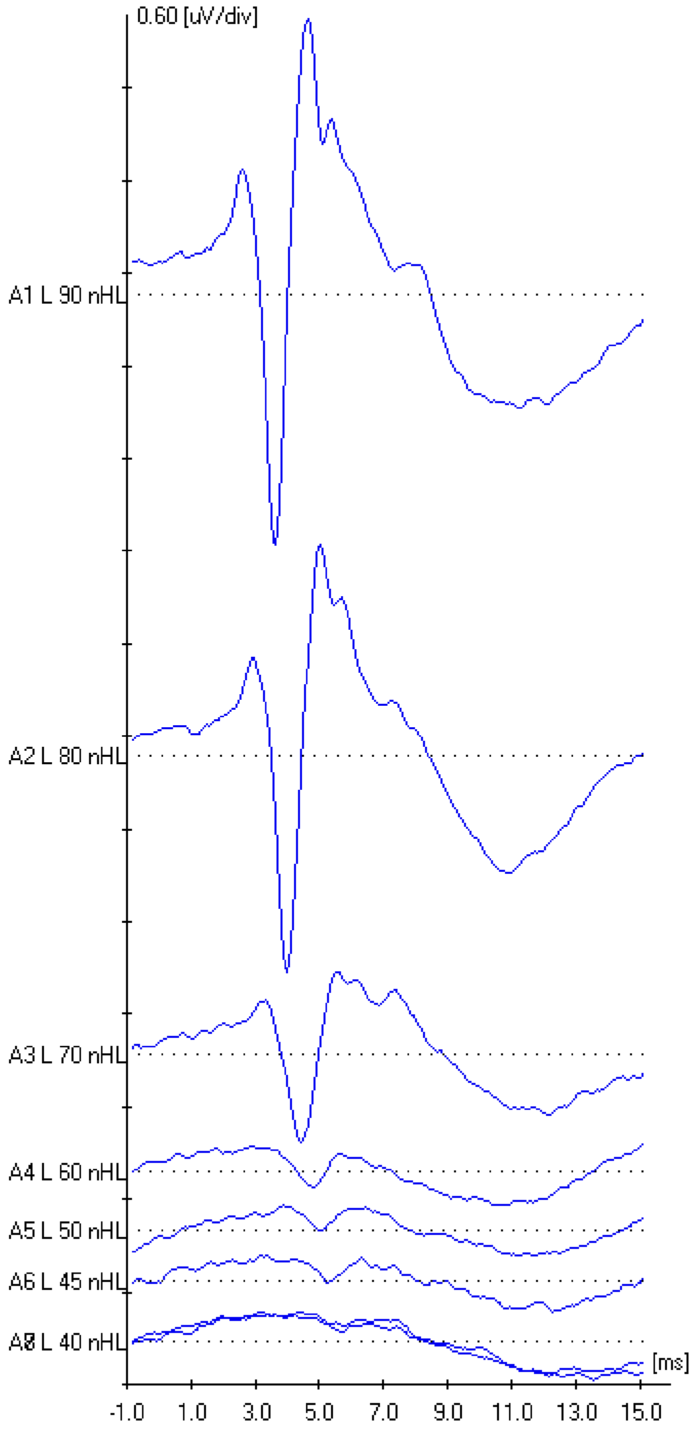

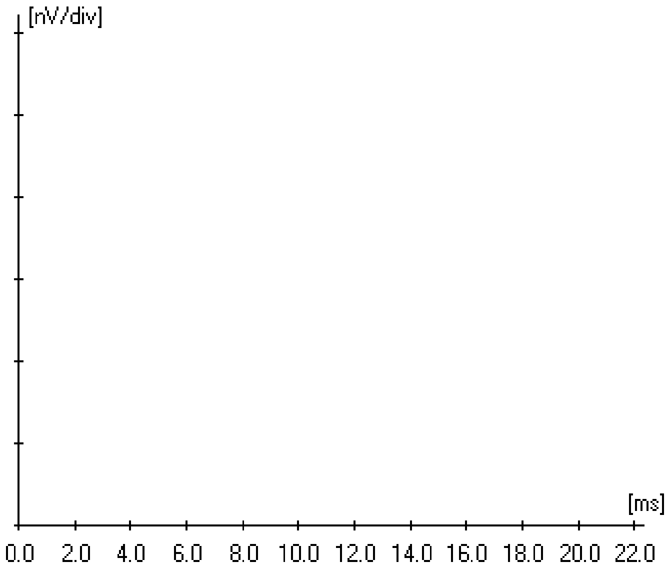

Latencies (ms)

Label Index	*I*	*II*	*III*	*IV*	*V*

Interlatencies (ms)

Label Index	*I-III*	*III-V*	*I-V*

Interaural Latency Differences

Label Index	*L1*	*L2*	*L3*	*L4*	*L5*	*L6*	*L7*	*L8*	*L9*	*L10*

Stimulus Parameters

Label Index	*Intensity*	*Ear*	*Transducer*	*Insert Delay*	*Type*	*Frequency*	*Polarity*	*Ramp*	*Rise/Fall*	*Plateau*	*Rate*
**A1**	**90dB nHL**	**Left**	**Insert Earphones**	**0.80**	**Tone Burst**	**2000**	**Alternating**	**Blackman**	**2.00**	**2.00**	**27.70**
**A2**	**80dB nHL**	**Left**	**Insert Earphones**	**0.80**	**Tone Burst**	**2000**	**Alternating**	**Blackman**	**2.00**	**2.00**	**27.70**

A3	70dB nHL	Left	Insert Earphones	0.80	Tone Burst	2000	Alternating	Blackman	2.00	2.00	27.70
A4	60dB nHL	Left	Insert Earphones	0.80	Tone Burst	2000	Alternating	Blackman	2.00	2.00	27.70
A5	50dB nHL	Left	Insert Earphones	0.80	Tone Burst	2000	Alternating	Blackman	2.00	2.00	27.70
A6	45dB nHL	Left	Insert Earphones	0.80	Tone Burst	2000	Alternating	Blackman	2.00	2.00	27.70
A7	40dB nHL	Left	Insert Earphones	0.80	Tone Burst	2000	Alternating	Blackman	2.00	2.00	27.70
A8	40dB nHL	Left	Insert Earphones	0.80	Tone Burst	2000	Alternating	Blackman	2.00	2.00	27.70

Recording Parameters

Label Index	*Epoch*	*Points*	*Pre/Post*	*Averages*	*Artifacts*
A1	16.00	256	0.00	909	48
A2	16.00	256	0.00	1100	53
A3	16.00	256	0.00	902	43
A4	16.00	256	0.00	2612	116
A5	16.00	256	0.00	1903	83
A6	16.00	256	0.00	986	50
A7	16.00	256	0.00	3297	164
A8	16.00	256	0.00	2200	114

Amplifier Parameters

Label Index	*Channel*	*Gain*	*Low Filter*	*High Filter*	*Notch Filter*	*Artifact Rejection*	*Input 1*	*Input 2*
A1	1	100000	30	1500	No	50.00	FZ	A1A2
A2	1	100000	30	1500	No	50.00	FZ	A1A2
A3	1	100000	30	1500	No	50.00	FZ	A1A2
A4	1	100000	30	1500	No	50.00	FZ	A1A2
A5	1	100000	30	1500	No	50.00	FZ	A1A2
A6	1	100000	30	1500	No	50.00	FZ	A1A2
A7	1	100000	30	1500	No	50.00	FZ	A1A2
A8	1	100000	30	1500	No	50.00	FZ	A1A2
