# Supplementary material for: Auditory brainstem responses in the nine-banded armadillo (Dasypus novemcinctus)
Source: PeerJ. 2023 Dec 13;11:e16602. doi: 10.7717/peerj.16602 (PMC10725177; doi:10.7717/peerj.16602)
Supplement: Supplemental Information 2 — Each raw data file shows ABR amplitude (blue line) across various stimulus intensities (indicated on y-axis) over time in milliseconds (indicated on x-axis) for a particular experiment. [file peerj-11-16602-s002.zip › Armadillo 2021/Animal 15-01 Case 15-07/All other frequencies by record number.pdf]

# EVOKED POTENTIAL REPORT

UAMS CHP Speech and Hearing Clinic
Department of Audiology and Speech Pathology
4021 W. 8th Street
Little Rock, AR 72204
(501) 320-7300

*Patient:* **Case 1507 animal 15-01, Armadillo**

*ID#:* **Armadillo 1507**

*Gender:*

*Birth date:* **02/11/15**

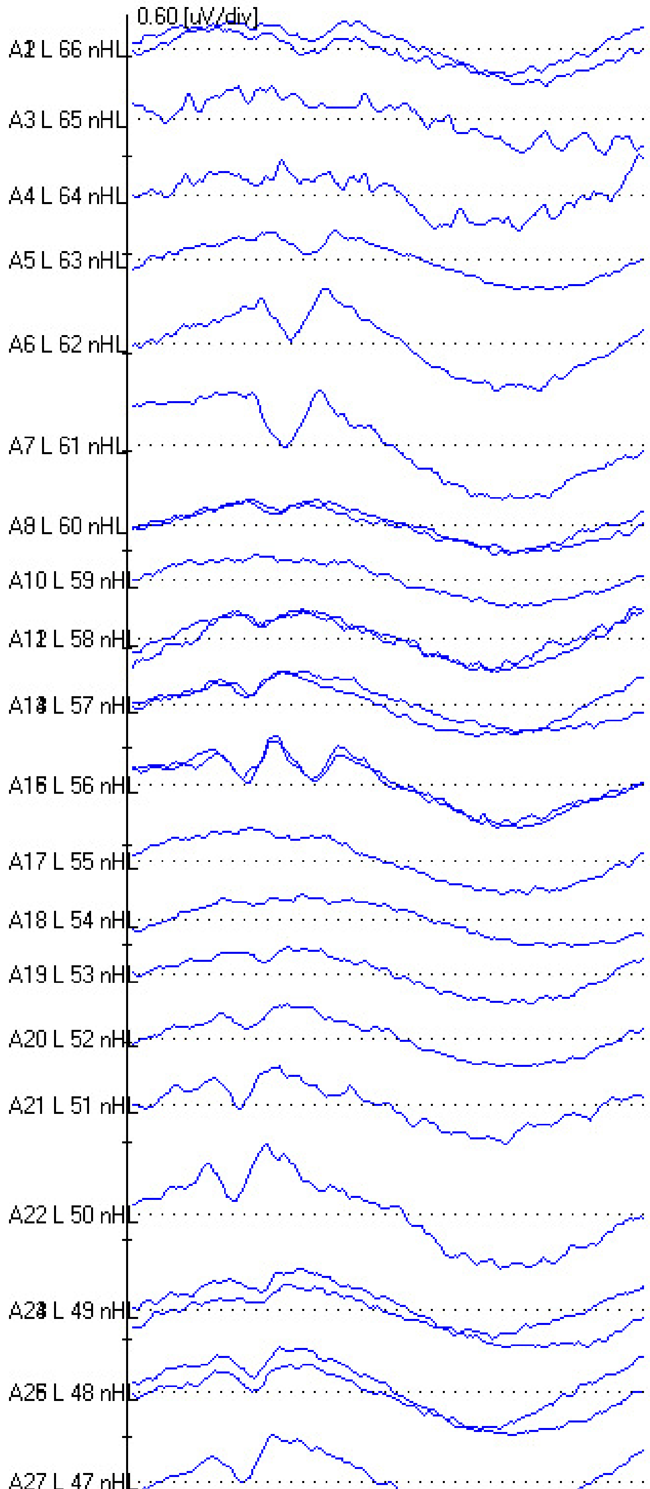


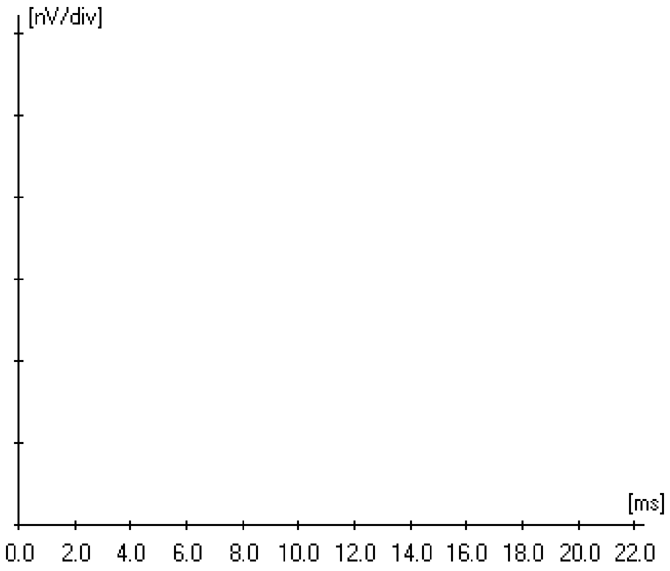

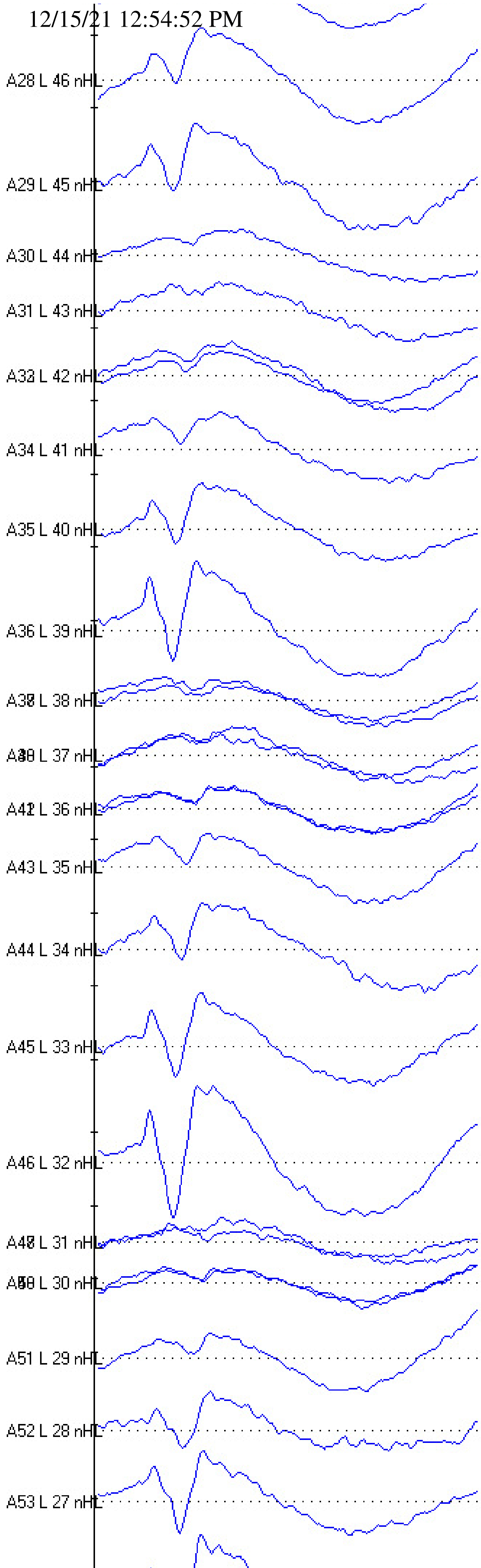
A28 L 46 nHL
A29 L 45 nHL
A30 L 44 nHL
A31 L 43 nHL
A34 L 41 nHL
A35 L 40 nHL
A36 L 39 nHL
A43 L 35 nHL
A44 L 34 nHL
A45 L 33 nHL
A46 L 32 nHL
A51 L 29 nHL
A52 L 28 nHL
A53 L 27 nHL

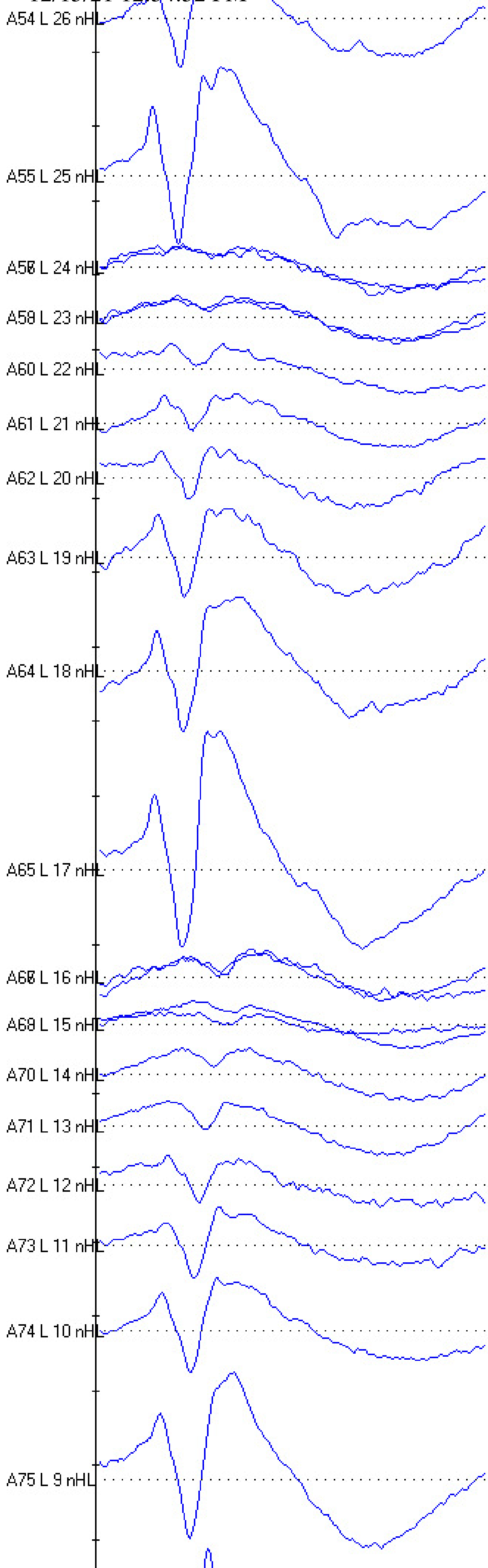
A54 L 26 nHL
A55 L 25 nHL
A58 L 23 nHL
A60 L 22 nHL
A61 L 21 nHL
A62 L 20 nHL
A63 L 19 nHL
A64 L 18 nHL
A65 L 17 nHL
A70 L 14 nHL
A71 L 13 nHL
A72 L 12 nHL
A73 L 11 nHL
A74 L 10 nHL
A75 L 9 nHL

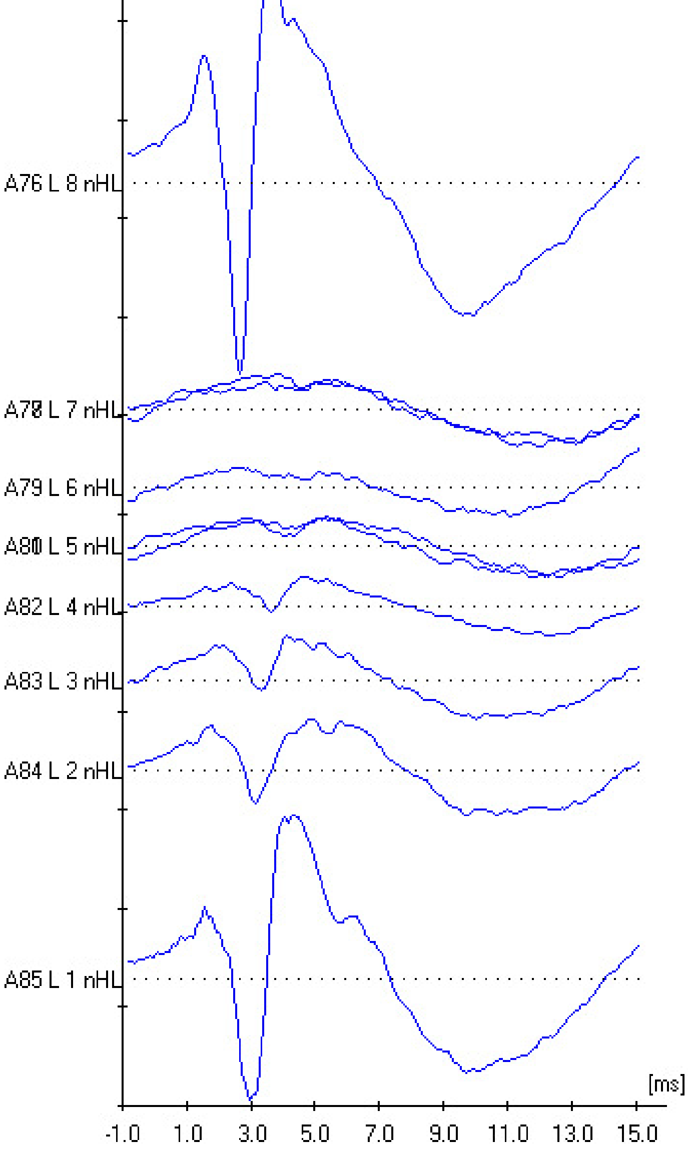


## *Latencies (ms)*

| *Label Index* | *I* | *II* | *III* | *IV* | *V* |
|---|---|---|---|---|---|

## *Interlatencies (ms)*

| *Label Index* | *I-III* | *III-V* | *I-V* |
|---|---|---|---|

## *Interaural Latency Differences*

| *Label Index* | *L1* | *L2* | *L3* | *L4* | *L5* | *L6* | *L7* | *L8* | *L9* | *L10* |
|---|---|---|---|---|---|---|---|---|---|---|

## *Stimulus Parameters*

| *Label Index* | *Intensity* | *Ear* | *Transducer* | *Insert Delay* | *Type* | *Frequency* | *Polarity* | *Ramp* | *Rise/Fall* | *Plateau* | *Rate* |
|---|---|---|---|---|---|---|---|---|---|---|---|
| **A1** | **66dB nHL** | **Left** | **Insert Earphones** | **0.80** | **Tone Burst** | **2000** | **Alternating** | **Blackman** | **2.00** | **2.00** | **27.70** |
| **A2** | **66dB nHL** | **Left** | **Insert Earphones** | **0.80** | **Tone Burst** | **2000** | **Alternating** | **Blackman** | **2.00** | **2.00** | **27.70** |

| | | | | | | | | | | | |
|---|---|---|---|---|---|---|---|---|---|---|---|
| A3 | 65dB nHL | Left | Insert Earphones | 0.80 | Tone Burst | 2000 | Alternating | Blackman | 2.00 | 2.00 | 27.70 |
| A4 | 64dB nHL | Left | Insert Earphones | 0.80 | Tone Burst | 2000 | Alternating | Blackman | 2.00 | 2.00 | 27.70 |
| A5 | 63dB nHL | Left | Insert Earphones | 0.80 | Tone Burst | 2000 | Alternating | Blackman | 2.00 | 2.00 | 27.70 |
| A6 | 62dB nHL | Left | Insert Earphones | 0.80 | Tone Burst | 2000 | Alternating | Blackman | 2.00 | 2.00 | 27.70 |
| A7 | 61dB nHL | Left | Insert Earphones | 0.80 | Tone Burst | 2000 | Alternating | Blackman | 2.00 | 2.00 | 27.70 |
| A8 | 60dB nHL | Left | Insert Earphones | 0.80 | Tone Burst | 2000 | Alternating | Blackman | 2.00 | 2.00 | 27.70 |
| A9 | 60dB nHL | Left | Insert Earphones | 0.80 | Tone Burst | 2000 | Alternating | Blackman | 2.00 | 2.00 | 27.70 |
| A10 | 59dB nHL | Left | Insert Earphones | 0.80 | Tone Burst | 2000 | Alternating | Blackman | 2.00 | 2.00 | 27.70 |
| A11 | 58dB nHL | Left | Insert Earphones | 0.80 | Tone Burst | 2000 | Alternating | Blackman | 2.00 | 2.00 | 27.70 |
| A12 | 58dB nHL | Left | Insert Earphones | 0.80 | Tone Burst | 2000 | Alternating | Blackman | 2.00 | 2.00 | 27.70 |
| A13 | 57dB nHL | Left | Insert Earphones | 0.80 | Tone Burst | 2000 | Alternating | Blackman | 2.00 | 2.00 | 27.70 |
| A14 | 57dB nHL | Left | Insert Earphones | 0.80 | Tone Burst | 2000 | Alternating | Blackman | 2.00 | 2.00 | 27.70 |
| A15 | 56dB nHL | Left | Insert Earphones | 0.80 | Tone Burst | 2000 | Alternating | Blackman | 2.00 | 2.00 | 27.70 |
| A16 | 56dB nHL | Left | Insert Earphones | 0.80 | Tone Burst | 2000 | Alternating | Blackman | 2.00 | 2.00 | 27.70 |
| A17 | 55dB nHL | Left | Insert Earphones | 0.80 | Tone Burst | 2000 | Alternating | Blackman | 2.00 | 2.00 | 27.70 |
| A18 | 54dB nHL | Left | Insert Earphones | 0.80 | Tone Burst | 2000 | Alternating | Blackman | 2.00 | 2.00 | 27.70 |
| A19 | 53dB nHL | Left | Insert Earphones | 0.80 | Tone Burst | 2000 | Alternating | Blackman | 2.00 | 2.00 | 27.70 |
| A20 | 52dB nHL | Left | Insert Earphones | 0.80 | Tone Burst | 2000 | Alternating | Blackman | 2.00 | 2.00 | 27.70 |
| A21 | 51dB nHL | Left | Insert Earphones | 0.80 | Tone Burst | 2000 | Alternating | Blackman | 2.00 | 2.00 | 27.70 |
| A22 | 50dB nHL | Left | Insert Earphones | 0.80 | Tone Burst | 2000 | Alternating | Blackman | 2.00 | 2.00 | 27.70 |
| A23 | 49dB nHL | Left | Insert Earphones | 0.80 | Tone Burst | 2000 | Alternating | Blackman | 2.00 | 2.00 | 27.70 |
| A24 | 49dB nHL | Left | Insert Earphones | 0.80 | Tone Burst | 2000 | Alternating | Blackman | 2.00 | 2.00 | 27.70 |
| A25 | 48dB nHL | Left | Insert Earphones | 0.80 | Tone Burst | 2000 | Alternating | Blackman | 2.00 | 2.00 | 27.70 |
| A26 | 48dB nHL | Left | Insert Earphones | 0.80 | Tone Burst | 2000 | Alternating | Blackman | 2.00 | 2.00 | 27.70 |
| A27 | 47dB nHL | Left | Insert Earphones | 0.80 | Tone Burst | 2000 | Alternating | Blackman | 2.00 | 2.00 | 27.70 |
| A28 | 46dB nHL | Left | Insert Earphones | 0.80 | Tone Burst | 2000 | Alternating | Blackman | 2.00 | 2.00 | 27.70 |
| A29 | 45dB nHL | Left | Insert Earphones | 0.80 | Tone Burst | 2000 | Alternating | Blackman | 2.00 | 2.00 | 27.70 |
| A30 | 44dB nHL | Left | Insert Earphones | 0.80 | Tone Burst | 2000 | Alternating | Blackman | 2.00 | 2.00 | 27.70 |
| A31 | 43dB nHL | Left | Insert Earphones | 0.80 | Tone Burst | 2000 | Alternating | Blackman | 2.00 | 2.00 | 27.70 |
| A32 | 42dB nHL | Left | Insert Earphones | 0.80 | Tone Burst | 2000 | Alternating | Blackman | 2.00 | 2.00 | 27.70 |
| A33 | 42dB nHL | Left | Insert Earphones | 0.80 | Tone Burst | 2000 | Alternating | Blackman | 2.00 | 2.00 | 27.70 |
| A34 | 41dB nHL | Left | Insert Earphones | 0.80 | Tone Burst | 2000 | Alternating | Blackman | 2.00 | 2.00 | 27.70 |
| A35 | 40dB nHL | Left | Insert Earphones | 0.80 | Tone Burst | 2000 | Alternating | Blackman | 2.00 | 2.00 | 27.70 |
| A36 | 39dB nHL | Left | Insert Earphones | 0.80 | Tone Burst | 2000 | Alternating | Blackman | 2.00 | 2.00 | 27.70 |
| A37 | 38dB nHL | Left | Insert Earphones | 0.80 | Tone Burst | 2000 | Alternating | Blackman | 2.00 | 2.00 | 27.70 |
| A38 | 38dB nHL | Left | Insert Earphones | 0.80 | Tone Burst | 2000 | Alternating | Blackman | 2.00 | 2.00 | 27.70 |
| A39 | 37dB nHL | Left | Insert Earphones | 0.80 | Tone Burst | 2000 | Alternating | Blackman | 2.00 | 2.00 | 27.70 |
| A40 | 37dB nHL | Left | Insert Earphones | 0.80 | Tone Burst | 2000 | Alternating | Blackman | 2.00 | 2.00 | 27.70 |
| A41 | 36dB nHL | Left | Insert Earphones | 0.80 | Tone Burst | 2000 | Alternating | Blackman | 2.00 | 2.00 | 27.70 |
| A42 | 36dB nHL | Left | Insert Earphones | 0.80 | Tone Burst | 2000 | Alternating | Blackman | 2.00 | 2.00 | 27.70 |
| A43 | 35dB nHL | Left | Insert Earphones | 0.80 | Tone Burst | 2000 | Alternating | Blackman | 2.00 | 2.00 | 27.70 |
| A44 | 34dB nHL | Left | Insert Earphones | 0.80 | Tone Burst | 2000 | Alternating | Blackman | 2.00 | 2.00 | 27.70 |

| | | | | | | | | | | | |
|---|---|---|---|---|---|---|---|---|---|---|---|
| A45 | 33dB nHL | Left | Insert Earphones | 0.80 | Tone Burst | 2000 | Alternating | Blackman | 2.00 | 2.00 | 27.70 |
| A46 | 32dB nHL | Left | Insert Earphones | 0.80 | Tone Burst | 2000 | Alternating | Blackman | 2.00 | 2.00 | 27.70 |
| A47 | 31dB nHL | Left | Insert Earphones | 0.80 | Tone Burst | 2000 | Alternating | Blackman | 2.00 | 2.00 | 27.70 |
| A48 | 31dB nHL | Left | Insert Earphones | 0.80 | Tone Burst | 2000 | Alternating | Blackman | 2.00 | 2.00 | 27.70 |
| A49 | 30dB nHL | Left | Insert Earphones | 0.80 | Tone Burst | 2000 | Alternating | Blackman | 2.00 | 2.00 | 27.70 |
| A50 | 30dB nHL | Left | Insert Earphones | 0.80 | Tone Burst | 2000 | Alternating | Blackman | 2.00 | 2.00 | 27.70 |
| A51 | 29dB nHL | Left | Insert Earphones | 0.80 | Tone Burst | 2000 | Alternating | Blackman | 2.00 | 2.00 | 27.70 |
| A52 | 28dB nHL | Left | Insert Earphones | 0.80 | Tone Burst | 2000 | Alternating | Blackman | 2.00 | 2.00 | 27.70 |
| A53 | 27dB nHL | Left | Insert Earphones | 0.80 | Tone Burst | 2000 | Alternating | Blackman | 2.00 | 2.00 | 27.70 |
| A54 | 26dB nHL | Left | Insert Earphones | 0.80 | Tone Burst | 2000 | Alternating | Blackman | 2.00 | 2.00 | 27.70 |
| A55 | 25dB nHL | Left | Insert Earphones | 0.80 | Tone Burst | 2000 | Alternating | Blackman | 2.00 | 2.00 | 27.70 |
| A56 | 24dB nHL | Left | Insert Earphones | 0.80 | Tone Burst | 2000 | Alternating | Blackman | 2.00 | 2.00 | 27.70 |
| A57 | 24dB nHL | Left | Insert Earphones | 0.80 | Tone Burst | 2000 | Alternating | Blackman | 2.00 | 2.00 | 27.70 |
| A58 | 23dB nHL | Left | Insert Earphones | 0.80 | Tone Burst | 2000 | Alternating | Blackman | 2.00 | 2.00 | 27.70 |
| A59 | 23dB nHL | Left | Insert Earphones | 0.80 | Tone Burst | 2000 | Alternating | Blackman | 2.00 | 2.00 | 27.70 |
| A60 | 22dB nHL | Left | Insert Earphones | 0.80 | Tone Burst | 2000 | Alternating | Blackman | 2.00 | 2.00 | 27.70 |
| A61 | 21dB nHL | Left | Insert Earphones | 0.80 | Tone Burst | 2000 | Alternating | Blackman | 2.00 | 2.00 | 27.70 |
| A62 | 20dB nHL | Left | Insert Earphones | 0.80 | Tone Burst | 2000 | Alternating | Blackman | 2.00 | 2.00 | 27.70 |
| A63 | 19dB nHL | Left | Insert Earphones | 0.80 | Tone Burst | 2000 | Alternating | Blackman | 2.00 | 2.00 | 27.70 |
| A64 | 18dB nHL | Left | Insert Earphones | 0.80 | Tone Burst | 2000 | Alternating | Blackman | 2.00 | 2.00 | 27.70 |
| A65 | 17dB nHL | Left | Insert Earphones | 0.80 | Tone Burst | 2000 | Alternating | Blackman | 2.00 | 2.00 | 27.70 |
| A66 | 16dB nHL | Left | Insert Earphones | 0.80 | Tone Burst | 2000 | Alternating | Blackman | 2.00 | 2.00 | 27.70 |
| A67 | 16dB nHL | Left | Insert Earphones | 0.80 | Tone Burst | 2000 | Alternating | Blackman | 2.00 | 2.00 | 27.70 |
| A68 | 15dB nHL | Left | Insert Earphones | 0.80 | Tone Burst | 2000 | Alternating | Blackman | 2.00 | 2.00 | 27.70 |
| A69 | 15dB nHL | Left | Insert Earphones | 0.80 | Tone Burst | 2000 | Alternating | Blackman | 2.00 | 2.00 | 27.70 |
| A70 | 14dB nHL | Left | Insert Earphones | 0.80 | Tone Burst | 2000 | Alternating | Blackman | 2.00 | 2.00 | 27.70 |
| A71 | 13dB nHL | Left | Insert Earphones | 0.80 | Tone Burst | 2000 | Alternating | Blackman | 2.00 | 2.00 | 27.70 |
| A72 | 12dB nHL | Left | Insert Earphones | 0.80 | Tone Burst | 2000 | Alternating | Blackman | 2.00 | 2.00 | 27.70 |
| A73 | 11dB nHL | Left | Insert Earphones | 0.80 | Tone Burst | 2000 | Alternating | Blackman | 2.00 | 2.00 | 27.70 |
| A74 | 10dB nHL | Left | Insert Earphones | 0.80 | Tone Burst | 2000 | Alternating | Blackman | 2.00 | 2.00 | 27.70 |
| A75 | 9dB nHL | Left | Insert Earphones | 0.80 | Tone Burst | 2000 | Alternating | Blackman | 2.00 | 2.00 | 27.70 |
| A76 | 8dB nHL | Left | Insert Earphones | 0.80 | Tone Burst | 2000 | Alternating | Blackman | 2.00 | 2.00 | 27.70 |
| A77 | 7dB nHL | Left | Insert Earphones | 0.80 | Tone Burst | 2000 | Alternating | Blackman | 2.00 | 2.00 | 27.70 |
| A78 | 7dB nHL | Left | Insert Earphones | 0.80 | Tone Burst | 2000 | Alternating | Blackman | 2.00 | 2.00 | 27.70 |
| A79 | 6dB nHL | Left | Insert Earphones | 0.80 | Tone Burst | 2000 | Alternating | Blackman | 2.00 | 2.00 | 27.70 |
| A80 | 5dB nHL | Left | Insert Earphones | 0.80 | Tone Burst | 2000 | Alternating | Blackman | 2.00 | 2.00 | 27.70 |
| A81 | 5dB nHL | Left | Insert Earphones | 0.80 | Tone Burst | 2000 | Alternating | Blackman | 2.00 | 2.00 | 27.70 |
| A82 | 4dB nHL | Left | Insert Earphones | 0.80 | Tone Burst | 2000 | Alternating | Blackman | 2.00 | 2.00 | 27.70 |
| A83 | 3dB nHL | Left | Insert Earphones | 0.80 | Tone Burst | 2000 | Alternating | Blackman | 2.00 | 2.00 | 27.70 |
| A84 | 2dB nHL | Left | Insert Earphones | 0.80 | Tone Burst | 2000 | Alternating | Blackman | 2.00 | 2.00 | 27.70 |
| A85 | 1dB nHL | Left | Insert Earphones | 0.80 | Tone Burst | 2000 | Alternating | Blackman | 2.00 | 2.00 | 27.70 |

# Recording Parameters



| *Label Index* | *Epoch* | *Points* | *Pre/Post* | *Averages* | *Artifacts* |
|---|---|---|---|---|---|
| **A1** | **16.00** | **256** | **0.00** | **1016** | **56** |
| **A2** | **16.00** | **256** | **0.00** | **880** | **49** |
| **A3** | **16.00** | **256** | **0.00** | **1127** | **65** |
| **A4** | **16.00** | **256** | **0.00** | **705** | **39** |
| **A5** | **16.00** | **256** | **0.00** | **1588** | **87** |
| **A6** | **16.00** | **256** | **0.00** | **1002** | **55** |
| **A7** | **16.00** | **256** | **0.00** | **913** | **50** |
| **A8** | **16.00** | **256** | **0.00** | **1516** | **80** |
| **A9** | **16.00** | **256** | **0.00** | **1773** | **94** |
| **A10** | **16.00** | **256** | **0.00** | **1270** | **66** |
| **A11** | **16.00** | **256** | **0.00** | **1435** | **73** |
| **A12** | **16.00** | **256** | **0.00** | **494** | **26** |
| **A13** | **16.00** | **256** | **0.00** | **1568** | **87** |
| **A14** | **16.00** | **256** | **0.00** | **2115** | **113** |
| **A15** | **16.00** | **256** | **0.00** | **1019** | **50** |
| **A16** | **16.00** | **256** | **0.00** | **1037** | **59** |
| **A17** | **16.00** | **256** | **0.00** | **1647** | **84** |
| **A18** | **16.00** | **256** | **0.00** | **1619** | **88** |
| **A19** | **16.00** | **256** | **0.00** | **1905** | **99** |
| **A20** | **16.00** | **256** | **0.00** | **2542** | **133** |
| **A21** | **16.00** | **256** | **0.00** | **1665** | **91** |
| **A22** | **16.00** | **256** | **0.00** | **663** | **38** |
| **A23** | **16.00** | **256** | **0.00** | **1035** | **55** |
| **A24** | **16.00** | **256** | **0.00** | **1382** | **73** |
| **A25** | **16.00** | **256** | **0.00** | **1352** | **71** |
| **A26** | **16.00** | **256** | **0.00** | **2949** | **160** |
| **A27** | **16.00** | **256** | **0.00** | **1521** | **80** |
| **A28** | **16.00** | **256** | **0.00** | **940** | **43** |
| **A29** | **16.00** | **256** | **0.00** | **593** | **31** |
| **A30** | **16.00** | **256** | **0.00** | **1656** | **88** |
| **A31** | **16.00** | **256** | **0.00** | **926** | **54** |
| **A32** | **16.00** | **256** | **0.00** | **1287** | **66** |
| **A33** | **16.00** | **256** | **0.00** | **3198** | **161** |
| **A34** | **16.00** | **256** | **0.00** | **1123** | **67** |
| **A35** | **16.00** | **256** | **0.00** | **912** | **55** |
| **A36** | **16.00** | **256** | **0.00** | **1267** | **69** |
| **A37** | **16.00** | **256** | **0.00** | **1528** | **81** |
| **A38** | **16.00** | **256** | **0.00** | **1612** | **84** |
| **A39** | **16.00** | **256** | **0.00** | **1053** | **55** |
| **A40** | **16.00** | **256** | **0.00** | **1140** | **63** |

| | | | | | |
|---|---|---|---|---|---|
| [illegible] | 16.00 | 256 | 0.00 | 1581 | 79 |
| A42 | 16.00 | 256 | 0.00 | 1650 | 86 |
| A43 | 16.00 | 256 | 0.00 | 1237 | 61 |
| A44 | 16.00 | 256 | 0.00 | 664 | 38 |
| A45 | 16.00 | 256 | 0.00 | 848 | 47 |
| A46 | 16.00 | 256 | 0.00 | 886 | 43 |
| A47 | 16.00 | 256 | 0.00 | 1478 | 78 |
| A48 | 16.00 | 256 | 0.00 | 1031 | 53 |
| A49 | 16.00 | 256 | 0.00 | 2077 | 99 |
| A50 | 16.00 | 256 | 0.00 | 2106 | 100 |
| A51 | 16.00 | 256 | 0.00 | 1040 | 48 |
| A52 | 16.00 | 256 | 0.00 | 1523 | 80 |
| A53 | 16.00 | 256 | 0.00 | 1015 | 54 |
| A54 | 16.00 | 256 | 0.00 | 614 | 33 |
| A55 | 16.00 | 256 | 0.00 | 881 | 49 |
| A56 | 16.00 | 256 | 0.00 | 2025 | 102 |
| A57 | 16.00 | 256 | 0.00 | 1458 | 78 |
| A58 | 16.00 | 256 | 0.00 | 2559 | 119 |
| A59 | 16.00 | 256 | 0.00 | 2722 | 133 |
| A60 | 16.00 | 256 | 0.00 | 1743 | 94 |
| A61 | 16.00 | 256 | 0.00 | 1549 | 80 |
| A62 | 16.00 | 256 | 0.00 | 696 | 35 |
| A63 | 16.00 | 256 | 0.00 | 1061 | 49 |
| A64 | 16.00 | 256 | 0.00 | 672 | 33 |
| A65 | 16.00 | 256 | 0.00 | 1023 | 53 |
| A66 | 16.00 | 256 | 0.00 | 2032 | 100 |
| A67 | 16.00 | 256 | 0.00 | 2469 | 118 |
| A68 | 16.00 | 256 | 0.00 | 2500 | 135 |
| A69 | 16.00 | 256 | 0.00 | 3037 | 159 |
| A70 | 16.00 | 256 | 0.00 | 1550 | 80 |
| A71 | 16.00 | 256 | 0.00 | 1576 | 76 |
| A72 | 16.00 | 256 | 0.00 | 1287 | 75 |
| A73 | 16.00 | 256 | 0.00 | 918 | 54 |
| A74 | 16.00 | 256 | 0.00 | 1032 | 58 |
| A75 | 16.00 | 256 | 0.00 | 895 | 48 |
| A76 | 16.00 | 256 | 0.00 | 1014 | 52 |
| A77 | 16.00 | 256 | 0.00 | 1602 | 82 |
| A78 | 16.00 | 256 | 0.00 | 1206 | 64 |
| A79 | 16.00 | 256 | 0.00 | 1789 | 84 |
| A80 | 16.00 | 256 | 0.00 | 2010 | 103 |
| A81 | 16.00 | 256 | 0.00 | 2513 | 126 |
| A82 | 16.00 | 256 | 0.00 | 2455 | 127 |

| | | | | | |
|---|---|---|---|---|---|
| [illegible] | 16.00 | 256 | 0.00 | 1520 | 78 |
| A84 | 16.00 | 256 | 0.00 | 903 | 47 |
| A85 | 16.00 | 256 | 0.00 | 1288 | 65 |

## *Amplifier Parameters*

| *Label Index* | *Channel* | *Gain* | *Low Filter* | *High Filter* | *Notch Filter* | *Artifact Rejection* | *Input 1* | *Input 2* |
|---|---|---|---|---|---|---|---|---|
| A1 | 1 | 100000 | 30 | 1500 | No | 50.00 | FZ | A1A2 |
| A2 | 1 | 100000 | 30 | 1500 | No | 50.00 | FZ | A1A2 |
| A3 | 1 | 100000 | 30 | 1500 | No | 50.00 | FZ | A1A2 |
| A4 | 1 | 100000 | 30 | 1500 | No | 50.00 | FZ | A1A2 |
| A5 | 1 | 100000 | 30 | 1500 | No | 50.00 | FZ | A1A2 |
| A6 | 1 | 100000 | 30 | 1500 | No | 50.00 | FZ | A1A2 |
| A7 | 1 | 100000 | 30 | 1500 | No | 50.00 | FZ | A1A2 |
| A8 | 1 | 100000 | 30 | 1500 | No | 50.00 | FZ | A1A2 |
| A9 | 1 | 100000 | 30 | 1500 | No | 50.00 | FZ | A1A2 |
| A10 | 1 | 100000 | 30 | 1500 | No | 50.00 | FZ | A1A2 |
| A11 | 1 | 100000 | 30 | 1500 | No | 50.00 | FZ | A1A2 |
| A12 | 1 | 100000 | 30 | 1500 | No | 50.00 | FZ | A1A2 |
| A13 | 1 | 100000 | 30 | 1500 | No | 50.00 | FZ | A1A2 |
| A14 | 1 | 100000 | 30 | 1500 | No | 50.00 | FZ | A1A2 |
| A15 | 1 | 100000 | 30 | 1500 | No | 50.00 | FZ | A1A2 |
| A16 | 1 | 100000 | 30 | 1500 | No | 50.00 | FZ | A1A2 |
| A17 | 1 | 100000 | 30 | 1500 | No | 50.00 | FZ | A1A2 |
| A18 | 1 | 100000 | 30 | 1500 | No | 50.00 | FZ | A1A2 |
| A19 | 1 | 100000 | 30 | 1500 | No | 50.00 | FZ | A1A2 |
| A20 | 1 | 100000 | 30 | 1500 | No | 50.00 | FZ | A1A2 |
| A21 | 1 | 100000 | 30 | 1500 | No | 50.00 | FZ | A1A2 |
| A22 | 1 | 100000 | 30 | 1500 | No | 50.00 | FZ | A1A2 |
| A23 | 1 | 100000 | 30 | 1500 | No | 50.00 | FZ | A1A2 |
| A24 | 1 | 100000 | 30 | 1500 | No | 50.00 | FZ | A1A2 |
| A25 | 1 | 100000 | 30 | 1500 | No | 50.00 | FZ | A1A2 |
| A26 | 1 | 100000 | 30 | 1500 | No | 50.00 | FZ | A1A2 |
| A27 | 1 | 100000 | 30 | 1500 | No | 50.00 | FZ | A1A2 |
| A28 | 1 | 100000 | 30 | 1500 | No | 50.00 | FZ | A1A2 |
| A29 | 1 | 100000 | 30 | 1500 | No | 50.00 | FZ | A1A2 |
| A30 | 1 | 100000 | 30 | 1500 | No | 50.00 | FZ | A1A2 |
| A31 | 1 | 100000 | 30 | 1500 | No | 50.00 | FZ | A1A2 |
| A32 | 1 | 100000 | 30 | 1500 | No | 50.00 | FZ | A1A2 |
| A33 | 1 | 100000 | 30 | 1500 | No | 50.00 | FZ | A1A2 |
| A34 | 1 | 100000 | 30 | 1500 | No | 50.00 | FZ | A1A2 |
| A35 | 1 | 100000 | 30 | 1500 | No | 50.00 | FZ | A1A2 |
| A36 | 1 | 100000 | 30 | 1500 | No | 50.00 | FZ | A1A2 |
| A37 | 1 | 100000 | 30 | 1500 | No | 50.00 | FZ | A1A2 |

| | | | | | | | | |
|---|---|---|---|---|---|---|---|---|
| A38 | 1 | 100000 | 30 | 1500 | No | 50.00 | FZ | A1A2 |
| A39 | 1 | 100000 | 30 | 1500 | No | 50.00 | FZ | A1A2 |
| A40 | 1 | 100000 | 30 | 1500 | No | 50.00 | FZ | A1A2 |
| A41 | 1 | 100000 | 30 | 1500 | No | 50.00 | FZ | A1A2 |
| A42 | 1 | 100000 | 30 | 1500 | No | 50.00 | FZ | A1A2 |
| A43 | 1 | 100000 | 30 | 1500 | No | 50.00 | FZ | A1A2 |
| A44 | 1 | 100000 | 30 | 1500 | No | 50.00 | FZ | A1A2 |
| A45 | 1 | 100000 | 30 | 1500 | No | 50.00 | FZ | A1A2 |
| A46 | 1 | 100000 | 30 | 1500 | No | 50.00 | FZ | A1A2 |
| A47 | 1 | 100000 | 30 | 1500 | No | 50.00 | FZ | A1A2 |
| A48 | 1 | 100000 | 30 | 1500 | No | 50.00 | FZ | A1A2 |
| A49 | 1 | 100000 | 30 | 1500 | No | 50.00 | FZ | A1A2 |
| A50 | 1 | 100000 | 30 | 1500 | No | 50.00 | FZ | A1A2 |
| A51 | 1 | 100000 | 30 | 1500 | No | 50.00 | FZ | A1A2 |
| A52 | 1 | 100000 | 30 | 1500 | No | 50.00 | FZ | A1A2 |
| A53 | 1 | 100000 | 30 | 1500 | No | 50.00 | FZ | A1A2 |
| A54 | 1 | 100000 | 30 | 1500 | No | 50.00 | FZ | A1A2 |
| A55 | 1 | 100000 | 30 | 1500 | No | 50.00 | FZ | A1A2 |
| A56 | 1 | 100000 | 30 | 1500 | No | 50.00 | FZ | A1A2 |
| A57 | 1 | 100000 | 30 | 1500 | No | 50.00 | FZ | A1A2 |
| A58 | 1 | 100000 | 30 | 1500 | No | 50.00 | FZ | A1A2 |
| A59 | 1 | 100000 | 30 | 1500 | No | 50.00 | FZ | A1A2 |
| A60 | 1 | 100000 | 30 | 1500 | No | 50.00 | FZ | A1A2 |
| A61 | 1 | 100000 | 30 | 1500 | No | 50.00 | FZ | A1A2 |
| A62 | 1 | 100000 | 30 | 1500 | No | 50.00 | FZ | A1A2 |
| A63 | 1 | 100000 | 30 | 1500 | No | 50.00 | FZ | A1A2 |
| A64 | 1 | 100000 | 30 | 1500 | No | 50.00 | FZ | A1A2 |
| A65 | 1 | 100000 | 30 | 1500 | No | 50.00 | FZ | A1A2 |
| A66 | 1 | 100000 | 30 | 1500 | No | 50.00 | FZ | A1A2 |
| A67 | 1 | 100000 | 30 | 1500 | No | 50.00 | FZ | A1A2 |
| A68 | 1 | 100000 | 30 | 1500 | No | 50.00 | FZ | A1A2 |
| A69 | 1 | 100000 | 30 | 1500 | No | 50.00 | FZ | A1A2 |
| A70 | 1 | 100000 | 30 | 1500 | No | 50.00 | FZ | A1A2 |
| A71 | 1 | 100000 | 30 | 1500 | No | 50.00 | FZ | A1A2 |
| A72 | 1 | 100000 | 30 | 1500 | No | 50.00 | FZ | A1A2 |
| A73 | 1 | 100000 | 30 | 1500 | No | 50.00 | FZ | A1A2 |
| A74 | 1 | 100000 | 30 | 1500 | No | 50.00 | FZ | A1A2 |
| A75 | 1 | 100000 | 30 | 1500 | No | 50.00 | FZ | A1A2 |
| A76 | 1 | 100000 | 30 | 1500 | No | 50.00 | FZ | A1A2 |
| A77 | 1 | 100000 | 30 | 1500 | No | 50.00 | FZ | A1A2 |
| A78 | 1 | 100000 | 30 | 1500 | No | 50.00 | FZ | A1A2 |
| A79 | 1 | 100000 | 30 | 1500 | No | 50.00 | FZ | A1A2 |

| | | | | | | | | |
|---|---|---|---|---|---|---|---|---|
| A80 | 1 | 100000 | 30 | 1500 | No | 50.00 | FZ | A1A2 |
| A81 | 1 | 100000 | 30 | 1500 | No | 50.00 | FZ | A1A2 |
| A82 | 1 | 100000 | 30 | 1500 | No | 50.00 | FZ | A1A2 |
| A83 | 1 | 100000 | 30 | 1500 | No | 50.00 | FZ | A1A2 |
| A84 | 1 | 100000 | 30 | 1500 | No | 50.00 | FZ | A1A2 |
| A85 | 1 | 100000 | 30 | 1500 | No | 50.00 | FZ | A1A2 |
