# Supplementary material for: Auditory brainstem responses in the nine-banded armadillo (Dasypus novemcinctus)
Source: PeerJ. 2023 Dec 13;11:e16602. doi: 10.7717/peerj.16602 (PMC10725177; doi:10.7717/peerj.16602)
Supplement: Supplemental Information 2 — Each raw data file shows ABR amplitude (blue line) across various stimulus intensities (indicated on y-axis) over time in milliseconds (indicated on x-axis) for a particular experiment. [file peerj-11-16602-s002.zip › Armadillo 2021/#1 Animal F14-05 Case 15-08/All other frequencies by record number.pdf]

EVOKED POTENTIAL REPORT

UAMS CHP Speech and Hearing Clinic
Department of Audiology and Speech Pathology
4021 W. 8th Street
Little Rock, AR 72204
(501) 320-7300

Patient:	**Armadillo 1508, Armadillo 1508**
ID#:	**Armadillo 1508**
Gender:	
Birth date:	**03/02/15**

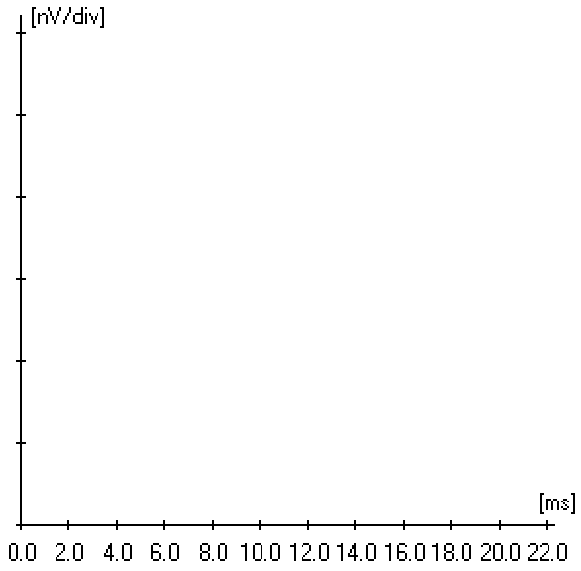

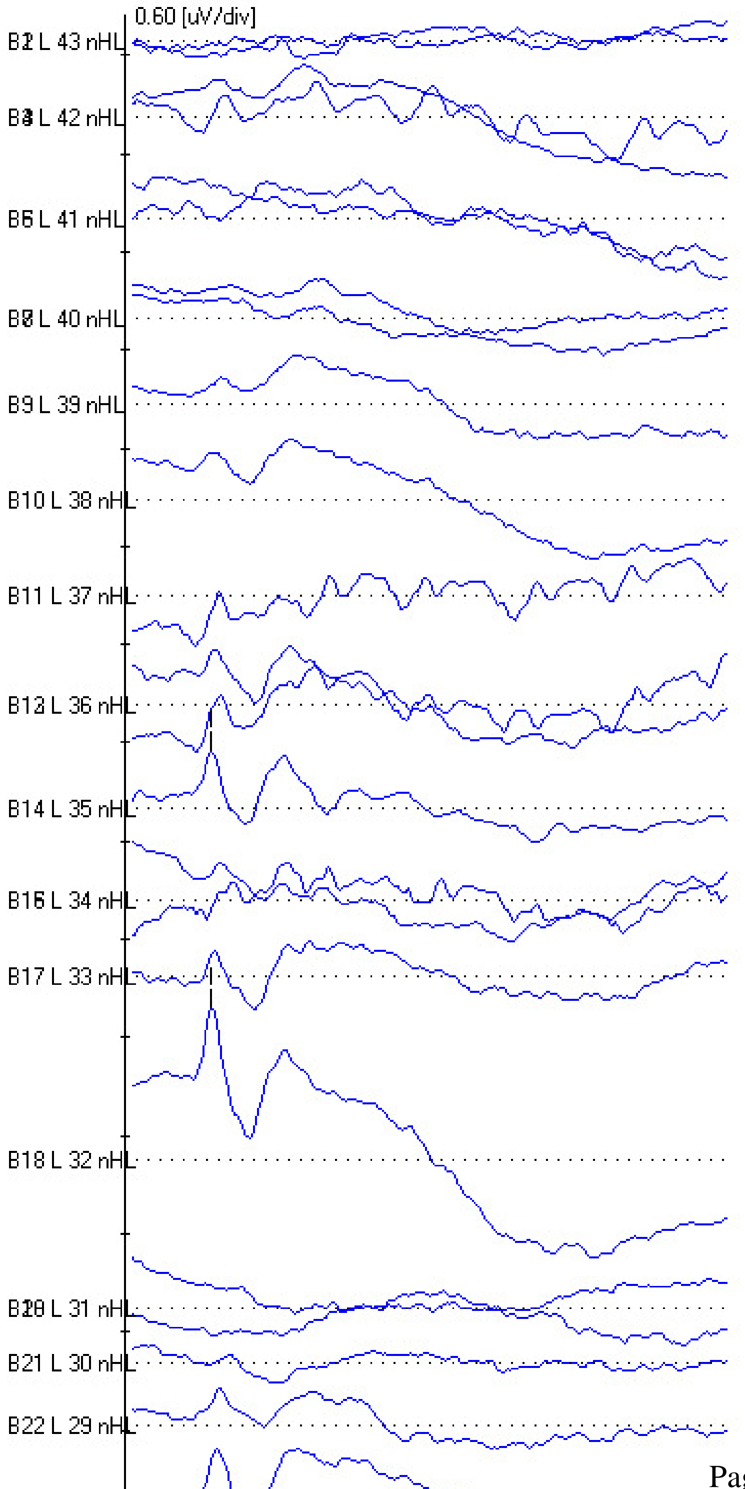

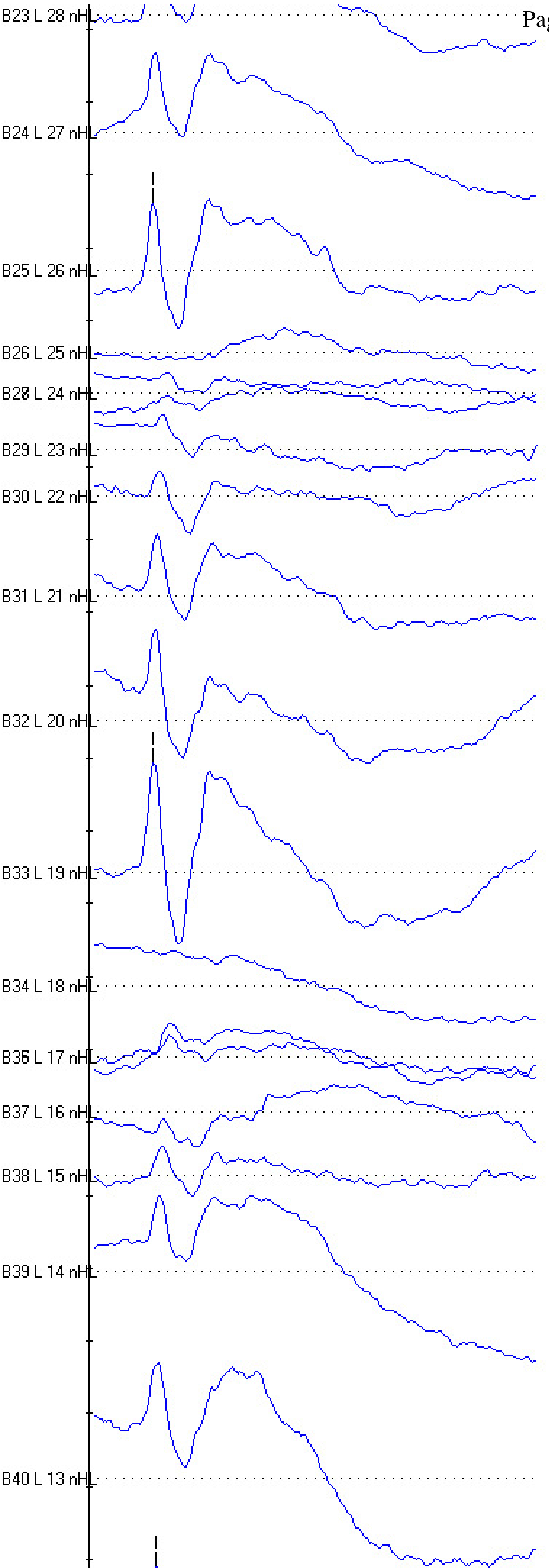
B23 L 28 nHL
B24 L 27 nHL
B25 L 26 nHL
B26 L 25 nHL
B28 L 24 nHL
B29 L 23 nHL
B30 L 22 nHL
B31 L 21 nHL
B32 L 20 nHL
B33 L 19 nHL
B34 L 18 nHL
B36 L 17 nHL
B37 L 16 nHL
B38 L 15 nHL
B39 L 14 nHL
B40 L 13 nHL

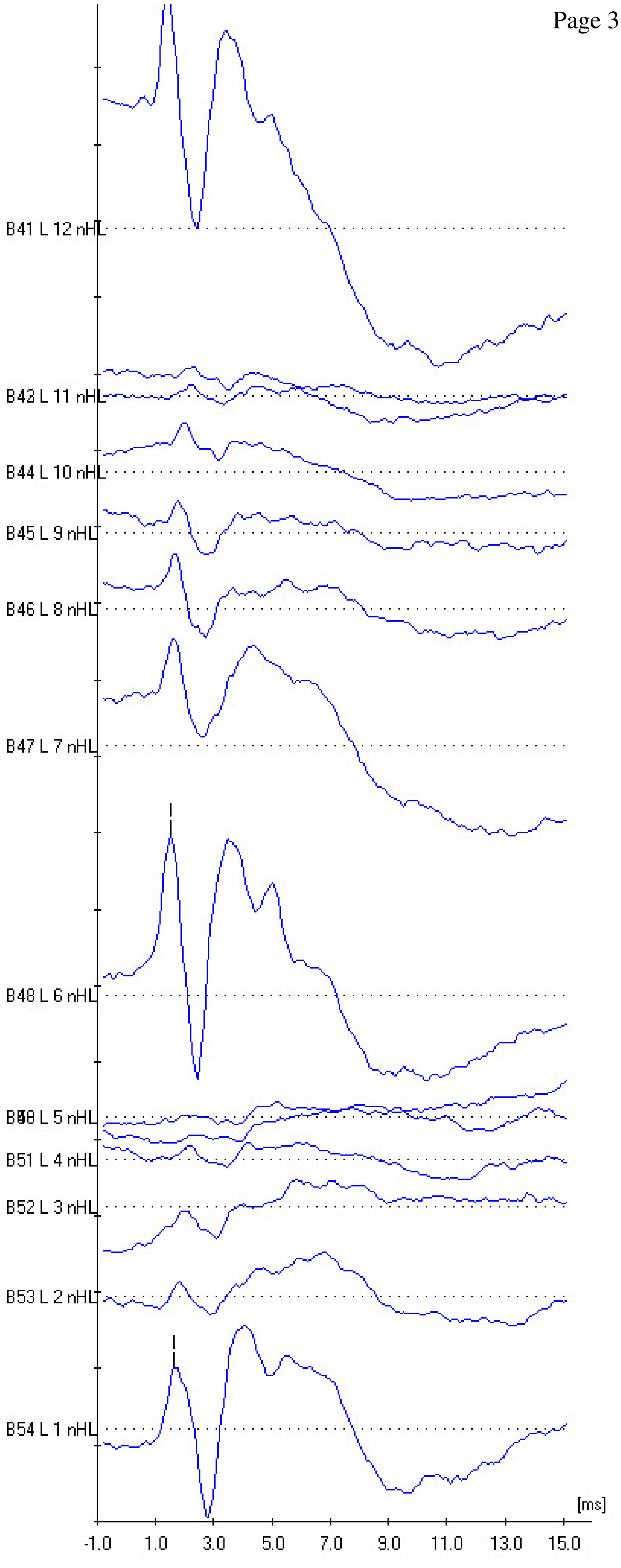
B41 L 12 nHL
B42 L 11 nHL
B43 L 11 nHL
B44 L 10 nHL
B45 L 9 nHL
B46 L 8 nHL
B47 L 7 nHL
B48 L 6 nHL
B50 L 5 nHL
B51 L 4 nHL
B52 L 3 nHL
B53 L 2 nHL
B54 L 1 nHL
[ms]
-1.0
1.0
3.0
5.0
7.0
9.0
11.0
13.0
15.0

Latencies (ms)

Label Index	*I*	*II*	*III*	*IV*	*V*
**B14**	**1.33**				
**B18**	**1.33**				
**B25**	**1.33**				
**B33**	**1.33**				
**B41**	**1.39**				
**B48**	**1.51**				
**B54**	**1.64**				

Interlatencies (ms)

Label Index	*I-III*	*III-V*	*I-V*

Interaural Latency Differences

Label Index	*L1*	*L2*	*L3*	*L4*	*L5*	*L6*	*L7*	*L8*	*L9*	*L10*

Stimulus Parameters

Label Index	*Intensity*	*Ear*	*Transducer*	*Insert Delay*	*Type*	*Frequency*	*Polarity*	*Ramp*	*Rise/Fall*	*Plateau*	*Rate*
**B1**	**43dB nHL**	**Left**	**Insert Earphones**	**0.80**	**Tone Burst**	**2000**	**Alternating**	**Blackman**	**2.00**	**2.00**	**27.70**
**B2**	**43dB nHL**	**Left**	**Insert Earphones**	**0.80**	**Tone Burst**	**2000**	**Alternating**	**Blackman**	**2.00**	**2.00**	**27.70**
**B3**	**42dB nHL**	**Left**	**Insert Earphones**	**0.80**	**Tone Burst**	**2000**	**Alternating**	**Blackman**	**2.00**	**2.00**	**27.70**
**B4**	**42dB nHL**	**Left**	**Insert Earphones**	**0.80**	**Tone Burst**	**2000**	**Alternating**	**Blackman**	**2.00**	**2.00**	**27.70**
**B5**	**41dB nHL**	**Left**	**Insert Earphones**	**0.80**	**Tone Burst**	**2000**	**Alternating**	**Blackman**	**2.00**	**2.00**	**27.70**
**B6**	**41dB nHL**	**Left**	**Insert Earphones**	**0.80**	**Tone Burst**	**2000**	**Alternating**	**Blackman**	**2.00**	**2.00**	**27.70**
**B7**	**40dB nHL**	**Left**	**Insert Earphones**	**0.80**	**Tone Burst**	**2000**	**Alternating**	**Blackman**	**2.00**	**2.00**	**27.70**
**B8**	**40dB nHL**	**Left**	**Insert Earphones**	**0.80**	**Tone Burst**	**2000**	**Alternating**	**Blackman**	**2.00**	**2.00**	**27.70**
**B9**	**39dB nHL**	**Left**	**Insert Earphones**	**0.80**	**Tone Burst**	**2000**	**Alternating**	**Blackman**	**2.00**	**2.00**	**27.70**
**B10**	**38dB nHL**	**Left**	**Insert Earphones**	**0.80**	**Tone Burst**	**2000**	**Alternating**	**Blackman**	**2.00**	**2.00**	**27.70**
**B11**	**37dB nHL**	**Left**	**Insert Earphones**	**0.80**	**Tone Burst**	**2000**	**Alternating**	**Blackman**	**2.00**	**2.00**	**27.70**
**B12**	**36dB nHL**	**Left**	**Insert Earphones**	**0.80**	**Tone Burst**	**2000**	**Alternating**	**Blackman**	**2.00**	**2.00**	**27.70**
**B13**	**36dB nHL**	**Left**	**Insert Earphones**	**0.80**	**Tone Burst**	**2000**	**Alternating**	**Blackman**	**2.00**	**2.00**	**27.70**
**B14**	**35dB nHL**	**Left**	**Insert Earphones**	**0.80**	**Tone Burst**	**2000**	**Alternating**	**Blackman**	**2.00**	**2.00**	**27.70**
**B15**	**34dB nHL**	**Left**	**Insert Earphones**	**0.80**	**Tone Burst**	**2000**	**Alternating**	**Blackman**	**2.00**	**2.00**	**27.70**
**B16**	**34dB nHL**	**Left**	**Insert Earphones**	**0.80**	**Tone Burst**	**2000**	**Alternating**	**Blackman**	**2.00**	**2.00**	**27.70**
**B17**	**33dB nHL**	**Left**	**Insert Earphones**	**0.80**	**Tone Burst**	**2000**	**Alternating**	**Blackman**	**2.00**	**2.00**	**27.70**
**B18**	**32dB nHL**	**Left**	**Insert Earphones**	**0.80**	**Tone Burst**	**2000**	**Alternating**	**Blackman**	**2.00**	**2.00**	**27.70**
**B19**	**31dB nHL**	**Left**	**Insert Earphones**	**0.80**	**Tone Burst**	**2000**	**Alternating**	**Blackman**	**2.00**	**2.00**	**27.70**
**B20**	**31dB nHL**	**Left**	**Insert Earphones**	**0.80**	**Tone Burst**	**2000**	**Alternating**	**Blackman**	**2.00**	**2.00**	**27.70**
**B21**	**30dB nHL**	**Left**	**Insert Earphones**	**0.80**	**Tone Burst**	**2000**	**Alternating**	**Blackman**	**2.00**	**2.00**	**27.70**
**B22**	**29dB nHL**	**Left**	**Insert Earphones**	**0.80**	**Tone Burst**	**2000**	**Alternating**	**Blackman**	**2.00**	**2.00**	**27.70**

**B23**	**28dB nHL**	**Left**	**Insert Earphones**	**0.80**	**Tone Burst**	**2000**	**Alternating**	**Blackman**	**2.00**	**2.00**	**27.70**
**B24**	**27dB nHL**	**Left**	**Insert Earphones**	**0.80**	**Tone Burst**	**2000**	**Alternating**	**Blackman**	**2.00**	**2.00**	**27.70**
**B25**	**26dB nHL**	**Left**	**Insert Earphones**	**0.80**	**Tone Burst**	**2000**	**Alternating**	**Blackman**	**2.00**	**2.00**	**27.70**
**B26**	**25dB nHL**	**Left**	**Insert Earphones**	**0.80**	**Tone Burst**	**2000**	**Alternating**	**Blackman**	**2.00**	**2.00**	**27.70**
**B27**	**24dB nHL**	**Left**	**Insert Earphones**	**0.80**	**Tone Burst**	**2000**	**Alternating**	**Blackman**	**2.00**	**2.00**	**27.70**
**B28**	**24dB nHL**	**Left**	**Insert Earphones**	**0.80**	**Tone Burst**	**2000**	**Alternating**	**Blackman**	**2.00**	**2.00**	**27.70**
**B29**	**23dB nHL**	**Left**	**Insert Earphones**	**0.80**	**Tone Burst**	**2000**	**Alternating**	**Blackman**	**2.00**	**2.00**	**27.70**
**B30**	**22dB nHL**	**Left**	**Insert Earphones**	**0.80**	**Tone Burst**	**2000**	**Alternating**	**Blackman**	**2.00**	**2.00**	**27.70**
**B31**	**21dB nHL**	**Left**	**Insert Earphones**	**0.80**	**Tone Burst**	**2000**	**Alternating**	**Blackman**	**2.00**	**2.00**	**27.70**
**B32**	**20dB nHL**	**Left**	**Insert Earphones**	**0.80**	**Tone Burst**	**2000**	**Alternating**	**Blackman**	**2.00**	**2.00**	**27.70**
**B33**	**19dB nHL**	**Left**	**Insert Earphones**	**0.80**	**Tone Burst**	**2000**	**Alternating**	**Blackman**	**2.00**	**2.00**	**27.70**
**B34**	**18dB nHL**	**Left**	**Insert Earphones**	**0.80**	**Tone Burst**	**2000**	**Alternating**	**Blackman**	**2.00**	**2.00**	**27.70**
**B35**	**17dB nHL**	**Left**	**Insert Earphones**	**0.80**	**Tone Burst**	**2000**	**Alternating**	**Blackman**	**2.00**	**2.00**	**27.70**
**B36**	**17dB nHL**	**Left**	**Insert Earphones**	**0.80**	**Tone Burst**	**2000**	**Alternating**	**Blackman**	**2.00**	**2.00**	**27.70**
**B37**	**16dB nHL**	**Left**	**Insert Earphones**	**0.80**	**Tone Burst**	**2000**	**Alternating**	**Blackman**	**2.00**	**2.00**	**27.70**
**B38**	**15dB nHL**	**Left**	**Insert Earphones**	**0.80**	**Tone Burst**	**2000**	**Alternating**	**Blackman**	**2.00**	**2.00**	**27.70**
**B39**	**14dB nHL**	**Left**	**Insert Earphones**	**0.80**	**Tone Burst**	**2000**	**Alternating**	**Blackman**	**2.00**	**2.00**	**27.70**
**B40**	**13dB nHL**	**Left**	**Insert Earphones**	**0.80**	**Tone Burst**	**2000**	**Alternating**	**Blackman**	**2.00**	**2.00**	**27.70**
**B41**	**12dB nHL**	**Left**	**Insert Earphones**	**0.80**	**Tone Burst**	**2000**	**Alternating**	**Blackman**	**2.00**	**2.00**	**27.70**
**B42**	**11dB nHL**	**Left**	**Insert Earphones**	**0.80**	**Tone Burst**	**2000**	**Alternating**	**Blackman**	**2.00**	**2.00**	**27.70**
**B43**	**11dB nHL**	**Left**	**Insert Earphones**	**0.80**	**Tone Burst**	**2000**	**Alternating**	**Blackman**	**2.00**	**2.00**	**27.70**
**B44**	**10dB nHL**	**Left**	**Insert Earphones**	**0.80**	**Tone Burst**	**2000**	**Alternating**	**Blackman**	**2.00**	**2.00**	**27.70**
**B45**	**9dB nHL**	**Left**	**Insert Earphones**	**0.80**	**Tone Burst**	**2000**	**Alternating**	**Blackman**	**2.00**	**2.00**	**27.70**
**B46**	**8dB nHL**	**Left**	**Insert Earphones**	**0.80**	**Tone Burst**	**2000**	**Alternating**	**Blackman**	**2.00**	**2.00**	**27.70**
**B47**	**7dB nHL**	**Left**	**Insert Earphones**	**0.80**	**Tone Burst**	**2000**	**Alternating**	**Blackman**	**2.00**	**2.00**	**27.70**
**B48**	**6dB nHL**	**Left**	**Insert Earphones**	**0.80**	**Tone Burst**	**2000**	**Alternating**	**Blackman**	**2.00**	**2.00**	**27.70**
**B49**	**5dB nHL**	**Left**	**Insert Earphones**	**0.80**	**Tone Burst**	**2000**	**Alternating**	**Blackman**	**2.00**	**2.00**	**27.70**
**B50**	**5dB nHL**	**Left**	**Insert Earphones**	**0.80**	**Tone Burst**	**2000**	**Alternating**	**Blackman**	**2.00**	**2.00**	**27.70**
**B51**	**4dB nHL**	**Left**	**Insert Earphones**	**0.80**	**Tone Burst**	**2000**	**Alternating**	**Blackman**	**2.00**	**2.00**	**27.70**
**B52**	**3dB nHL**	**Left**	**Insert Earphones**	**0.80**	**Tone Burst**	**2000**	**Alternating**	**Blackman**	**2.00**	**2.00**	**27.70**
**B53**	**2dB nHL**	**Left**	**Insert Earphones**	**0.80**	**Tone Burst**	**2000**	**Alternating**	**Blackman**	**2.00**	**2.00**	**27.70**
**B54**	**1dB nHL**	**Left**	**Insert Earphones**	**0.80**	**Tone Burst**	**2000**	**Alternating**	**Blackman**	**2.00**	**2.00**	**27.70**

Recording Parameters

Label Index	*Epoch*	*Points*	*Pre/Post*	*Averages*	*Artifacts*
**B1**	**16.00**	**256**	**0.00**	**956**	**9**
**B2**	**16.00**	**256**	**0.00**	**1019**	**7**
**B3**	**16.00**	**256**	**0.00**	**1239**	**8**
**B4**	**16.00**	**256**	**0.00**	**1418**	**11**
**B5**	**16.00**	**256**	**0.00**	**864**	**5**
**B6**	**16.00**	**256**	**0.00**	**806**	**9**
**B7**	**16.00**	**256**	**0.00**	**1382**	**7**
**B8**	**16.00**	**256**	**0.00**	**1624**	**10**

B9	16.00	256	0.00	761	6
B10	16.00	256	0.00	1250	7
B11	16.00	256	0.00	1279	11
B12	16.00	256	0.00	1153	7
B13	16.00	256	0.00	807	9
B14	16.00	256	0.00	1436	7
B15	16.00	256	0.00	1832	7
B16	16.00	256	0.00	1047	6
B17	16.00	256	0.00	824	5
B18	16.00	256	0.00	1020	6
B19	16.00	256	0.00	1161	6
B20	16.00	256	0.00	635	5
B21	16.00	256	0.00	1317	6
B22	16.00	256	0.00	1150	7
B23	16.00	256	0.00	857	6
B24	16.00	256	0.00	896	5
B25	16.00	256	0.00	644	6
B26	16.00	256	0.00	879	5
B27	16.00	256	0.00	1570	9
B28	16.00	256	0.00	1220	6
B29	16.00	256	0.00	830	7
B30	16.00	256	0.00	716	5
B31	16.00	256	0.00	677	7
B32	16.00	256	0.00	626	7
B33	16.00	256	0.00	630	6
B34	16.00	256	0.00	1107	8
B35	16.00	256	0.00	1060	5
B36	16.00	256	0.00	1020	9
B37	16.00	256	0.00	1212	10
B38	16.00	256	0.00	998	8
B39	16.00	256	0.00	835	8
B40	16.00	256	0.00	494	4
B41	16.00	256	0.00	385	4
B42	16.00	256	0.00	1299	6
B43	16.00	256	0.00	1085	7
B44	16.00	256	0.00	960	7
B45	16.00	256	0.00	706	6
B46	16.00	256	0.00	800	7
B47	16.00	256	0.00	779	8
B48	16.00	256	0.00	533	6
B49	16.00	256	0.00	1163	8
B50	16.00	256	0.00	1323	8

[illegible]	16.00	256	0.00	1819	8
B52	16.00	256	0.00	1050	7
B53	16.00	256	0.00	1125	7
B54	16.00	256	0.00	677	6

Amplifier Parameters

Label Index	*Channel*	*Gain*	*Low Filter*	*High Filter*	*Notch Filter*	*Artifact Rejection*	*Input 1*	*Input 2*
B1	1	100000	30	1500	No	50.00	FZ	A1A2
B2	1	100000	30	1500	No	50.00	FZ	A1A2
B3	1	100000	30	1500	No	50.00	FZ	A1A2
B4	1	100000	30	1500	No	50.00	FZ	A1A2
B5	1	100000	30	1500	No	50.00	FZ	A1A2
B6	1	100000	30	1500	No	50.00	FZ	A1A2
B7	1	100000	30	1500	No	50.00	FZ	A1A2
B8	1	100000	30	1500	No	50.00	FZ	A1A2
B9	1	100000	30	1500	No	50.00	FZ	A1A2
B10	1	100000	30	1500	No	50.00	FZ	A1A2
B11	1	100000	30	1500	No	50.00	FZ	A1A2
B12	1	100000	30	1500	No	50.00	FZ	A1A2
B13	1	100000	30	1500	No	50.00	FZ	A1A2
B14	1	100000	30	1500	No	50.00	FZ	A1A2
B15	1	100000	30	1500	No	50.00	FZ	A1A2
B16	1	100000	30	1500	No	50.00	FZ	A1A2
B17	1	100000	30	1500	No	50.00	FZ	A1A2
B18	1	100000	30	1500	No	50.00	FZ	A1A2
B19	1	100000	30	1500	No	50.00	FZ	A1A2
B20	1	100000	30	1500	No	50.00	FZ	A1A2
B21	1	100000	30	1500	No	50.00	FZ	A1A2
B22	1	100000	30	1500	No	50.00	FZ	A1A2
B23	1	100000	30	1500	No	50.00	FZ	A1A2
B24	1	100000	30	1500	No	50.00	FZ	A1A2
B25	1	100000	30	1500	No	50.00	FZ	A1A2
B26	1	100000	30	1500	No	50.00	FZ	A1A2
B27	1	100000	30	1500	No	50.00	FZ	A1A2
B28	1	100000	30	1500	No	50.00	FZ	A1A2
B29	1	100000	30	1500	No	50.00	FZ	A1A2
B30	1	100000	30	1500	No	50.00	FZ	A1A2
B31	1	100000	30	1500	No	50.00	FZ	A1A2
B32	1	100000	30	1500	No	50.00	FZ	A1A2
B33	1	100000	30	1500	No	50.00	FZ	A1A2
B34	1	100000	30	1500	No	50.00	FZ	A1A2
B35	1	100000	30	1500	No	50.00	FZ	A1A2
B36	1	100000	30	1500	No	50.00	FZ	A1A2

B37	1	100000	30	1500	No	50.00	FZ	A1A2
B38	1	100000	30	1500	No	50.00	FZ	A1A2
B39	1	100000	30	1500	No	50.00	FZ	A1A2
B40	1	100000	30	1500	No	50.00	FZ	A1A2
B41	1	100000	30	1500	No	50.00	FZ	A1A2
B42	1	100000	30	1500	No	50.00	FZ	A1A2
B43	1	100000	30	1500	No	50.00	FZ	A1A2
B44	1	100000	30	1500	No	50.00	FZ	A1A2
B45	1	100000	30	1500	No	50.00	FZ	A1A2
B46	1	100000	30	1500	No	50.00	FZ	A1A2
B47	1	100000	30	1500	No	50.00	FZ	A1A2
B48	1	100000	30	1500	No	50.00	FZ	A1A2
B49	1	100000	30	1500	No	50.00	FZ	A1A2
B50	1	100000	30	1500	No	50.00	FZ	A1A2
B51	1	100000	30	1500	No	50.00	FZ	A1A2
B52	1	100000	30	1500	No	50.00	FZ	A1A2
B53	1	100000	30	1500	No	50.00	FZ	A1A2
B54	1	100000	30	1500	No	50.00	FZ	A1A2
