# Supplementary material for: Auditory brainstem responses in the nine-banded armadillo (Dasypus novemcinctus)
Source: PeerJ. 2023 Dec 13;11:e16602. doi: 10.7717/peerj.16602 (PMC10725177; doi:10.7717/peerj.16602)

General Linear Model

[DataSet3]

Warnings

Box's Test of Equality of Covariance Matrices is not computed because there are fewer than two nonsingular cell covariance matrices.

Within-Subjects Factors

Measure: Thresh

Freq	Dependent Variable
1	@4KHz
2	@8kHz
3	@12kHz
4	@16kHz
5	@20kHz

Between-Subjects Factors

		N
Sex	1.00	5
	2.00	3

Descriptive Statistics

	Sex	Mean	Std. Deviation	N
4 KHz	1.00	39.2000	8.21584	5
	2.00	34.3667	5.77350	3
	Total	37.3875	7.37243	8
8 kHz	1.00	21.4800	11.51790	5
	2.00	34.3000	10.00000	3
	Total	26.2875	12.18201	8
12 kHz	1.00	21.2000	12.32376	5
	2.00	35.5333	15.27525	3
	Total	26.5750	14.43891	8
16 kHz	1.00	29.4000	16.11521	5
	2.00	36.4333	5.77350	3
	Total	32.0375	13.08335	8
20 kHz	1.00	36.3000	13.03840	5
	2.00	46.8000	10.00000	3
	Total	40.2375	12.45976	8

Multivariate Tests[a]

Effect		Value	F	Hypothesis df	Error df	Sig.
Freq	Pillai's Trace	.777	2.617[b]	4.000	3.000	.228
	Wilks' Lambda	.223	2.617[b]	4.000	3.000	.228
	Hotelling's Trace	3.489	2.617[b]	4.000	3.000	.228
	Roy's Largest Root	3.489	2.617[b]	4.000	3.000	.228
Freq * Sex	Pillai's Trace	.806	3.125[b]	4.000	3.000	.188
	Wilks' Lambda	.194	3.125[b]	4.000	3.000	.188
	Hotelling's Trace	4.167	3.125[b]	4.000	3.000	.188
	Roy's Largest Root	4.167	3.125[b]	4.000	3.000	.188

Multivariate Tests[a]

Effect		Partial Eta Squared	Noncent. Parameter	Observed Power[c]
Freq	Pillai's Trace	.777	10.468	.241
	Wilks' Lambda	.777	10.468	.241
	Hotelling's Trace	.777	10.468	.241
	Roy's Largest Root	.777	10.468	.241
Freq * Sex	Pillai's Trace	.806	12.500	.279
	Wilks' Lambda	.806	12.500	.279
	Hotelling's Trace	.806	12.500	.279
	Roy's Largest Root	.806	12.500	.279

a. Design: Intercept + Sex
Within Subjects Design: Freq

b. Exact statistic

c. Computed using alpha = .05

Mauchly's Test of Sphericity[a]

Measure: Thresh

Within Subjects Effect	Mauchly's W	Approx. Chi-Square	df	Sig.	Epsilon[b] Greenhouse-Geisser
Freq	.067	11.909	9	.249	.612

Mauchly's Test of Sphericity[a]

Measure: Thresh

Within Subjects Effect	Epsilon[b] Huynh-Feldt	Lower-bound
Freq	1.000	.250

Tests the null hypothesis that the error covariance matrix of the orthonormalized transformed dependent variables is proportional to an identity matrix.

a. Design: Intercept + Sex
Within Subjects Design: Freq

b. May be used to adjust the degrees of freedom for the averaged tests of significance. Corrected tests are displayed in the Tests of Within-Subjects Effects table.

Tests of Within-Subjects Effects

Measure: Thresh

Source		Type III Sum of Squares	df	Mean Square	F	Sig.
Freq	Sphericity Assumed	1003.097	4	250.774	3.893	.014
	Greenhouse-Geisser	1003.097	2.450	409.453	3.893	.037
	Huynh-Feldt	1003.097	4.000	250.774	3.893	.014
	Lower-bound	1003.097	1.000	1003.097	3.893	.096
Freq * Sex	Sphericity Assumed	441.034	4	110.258	1.712	.180
	Greenhouse-Geisser	441.034	2.450	180.025	1.712	.212
	Huynh-Feldt	441.034	4.000	110.258	1.712	.180
	Lower-bound	441.034	1.000	441.034	1.712	.239
Error(Freq)	Sphericity Assumed	1545.993	24	64.416		
	Greenhouse-Geisser	1545.993	14.699	105.176		
	Huynh-Feldt	1545.993	24.000	64.416		
	Lower-bound	1545.993	6.000	257.666		

Tests of Within-Subjects Effects

Measure: Thresh

Source		Partial Eta Squared	Noncent. Parameter	Observed Power[a]
Freq	Sphericity Assumed	.394	15.572	.830
	Greenhouse-Geisser	.394	9.537	.658
	Huynh-Feldt	.394	15.572	.830
	Lower-bound	.394	3.893	.383
Freq * Sex	Sphericity Assumed	.222	6.847	.445
	Greenhouse-Geisser	.222	4.193	.328
	Huynh-Feldt	.222	6.847	.445
	Lower-bound	.222	1.712	.198
Error(Freq)	Sphericity Assumed			
	Greenhouse-Geisser			
	Huynh-Feldt			
	Lower-bound			

a. Computed using alpha = .05

Tests of Within-Subjects Contrasts

Measure: Thresh

Source	Freq	Type III Sum of Squares	df	Mean Square	F	Sig.
Freq	Linear	158.995	1	158.995	2.318	.179
	Quadratic	820.123	1	820.123	9.177	.023
	Cubic	20.962	1	20.962	.282	.615
	Order 4	3.017	1	3.017	.119	.742
Freq * Sex	Linear	116.065	1	116.065	1.692	.241
	Quadratic	185.203	1	185.203	2.072	.200
	Cubic	135.744	1	135.744	1.825	.225
	Order 4	4.022	1	4.022	.159	.704
Error(Freq)	Linear	411.509	6	68.585		
	Quadratic	536.177	6	89.363		
	Cubic	446.302	6	74.384		
	Order 4	152.006	6	25.334		

Tests of Within-Subjects Contrasts

Measure: Thresh

Source	Freq	Partial Eta Squared	Noncent. Parameter	Observed Power[a]
Freq	Linear	.279	2.318	.251
	Quadratic	.605	9.177	.715
	Cubic	.045	.282	.074
	Order 4	.019	.119	.060
Freq * Sex	Linear	.220	1.692	.196
	Quadratic	.257	2.072	.230
	Cubic	.233	1.825	.208
	Order 4	.026	.159	.063
Error(Freq)	Linear			
	Quadratic			
	Cubic			
	Order 4			

a. Computed using alpha = .05

Levene's Test of Equality of Error Variances[a]

		Levene Statistic	df1	df2	Sig.
4 KHz	Based on Mean	.078	1	6	.789
	Based on Median	.062	1	6	.812
	Based on Median and with adjusted df	.062	1	5.895	.812
	Based on trimmed mean	.060	1	6	.815
8 kHz	Based on Mean	.057	1	6	.819
	Based on Median	.006	1	6	.941
	Based on Median and with adjusted df	.006	1	5.208	.941
	Based on trimmed mean	.030	1	6	.868
12 kHz	Based on Mean	.051	1	6	.829
	Based on Median	.007	1	6	.937
	Based on Median and with adjusted df	.007	1	5.477	.938
	Based on trimmed mean	.048	1	6	.834
16 kHz	Based on Mean	2.019	1	6	.205
	Based on Median	1.561	1	6	.258
	Based on Median and with adjusted df	1.561	1	5.126	.266
	Based on trimmed mean	2.146	1	6	.193
20 kHz	Based on Mean	.762	1	6	.416
	Based on Median	.104	1	6	.758
	Based on Median and with adjusted df	.104	1	4.929	.760
	Based on trimmed mean	.666	1	6	.446

Tests the null hypothesis that the error variance of the dependent variable is equal across groups.

a. Design: Intercept + Sex
Within Subjects Design: Freq

Tests of Between-Subjects Effects

Measure: Thresh

Transformed Variable: Average

Source	Type III Sum of Squares	df	Mean Square	F	Sig.	Partial Eta Squared
Intercept	42087.725	1	42087.725	97.842	.000	.942
Sex	595.608	1	595.608	1.385	.284	.188
Error	2580.955	6	430.159			

Tests of Between-Subjects Effects

Measure: Thresh

Transformed Variable: Average

Source	Noncent. Parameter	Observed Power[a]
Intercept	97.842	1.000
Sex	1.385	.169
Error		

a. Computed using alpha = .05

Profile Plots

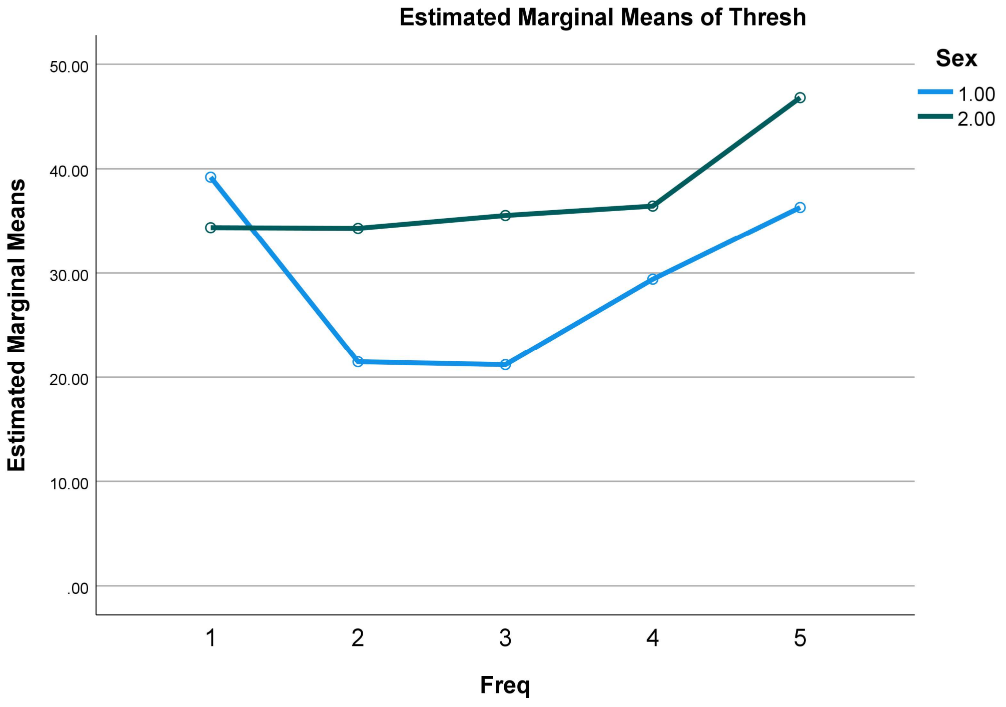

Supplement: Supplemental Information 4 — A 2-way mixed model ANOVA with frequency as a within-cases variable and sex as a between-groups variable was conducted (IBM SPSS Statistics for Windows, version 27 (IBM Corp., Armonk, N.Y., USA)). No effect of sex (F(1,6) = 1.385, p = 0.284, ηp2 = 0.188) nor any interaction (F(4,24) = 1.71, p = 0.180, ηp2 = 0.222) between sex and the significant effect of frequency ((F(4,24) = 3.89, p = 0.014, ηp2 = 0.394) was observed. [file peerj-11-16602-s004.pdf]
